# Frentizole, a Nontoxic Immunosuppressive Drug, and Its Analogs Display Antitumor Activity via Tubulin Inhibition

**DOI:** 10.3390/ijms242417474

**Published:** 2023-12-14

**Authors:** Sergio Ramos, Alba Vicente-Blázquez, Marta López-Rubio, Laura Gallego-Yerga, Raquel Álvarez, Rafael Peláez

**Affiliations:** 1 Laboratorio de Química Orgánica y Farmacéutica, Departamento de Ciencias Farmacéuticas, Campus Miguel de Unamuno, Universidad de Salamanca, 37008 Salamanca, Spain; sergio.rvarela@usal.es (S.R.); m_lopez@usal.es (M.L.-R.); gallego@usal.es (L.G.-Y.); raquelalvarez@usal.es (R.Á.); 2 Instituto de Investigación Biomédica de Salamanca (IBSAL), Facultad de Farmacia, Campus Miguel de Unamuno, Universidad de Salamanca, 37008 Salamanca, Spain; 3 Centro de Investigación de Enfermedades Tropicales de la Universidad de Salamanca (CIETUS), Facultad de Farmacia, Campus Miguel de Unamuno, Universidad de Salamanca, 37008 Salamanca, Spain

**Keywords:** drug repurposing, frentizole, tubulin, antimitotic, colchicine site, antitumor, drug design, synthesis, benzothiazole

## Abstract

Antimitotic agents are one of the more successful types of anticancer drugs, but they suffer from toxicity and resistance. The application of approved drugs to new indications (i.e., drug repurposing) is a promising strategy for the development of new drugs. It relies on finding pattern similarities: drug effects to other drugs or conditions, similar toxicities, or structural similarity. Here, we recursively searched a database of approved drugs for structural similarity to several antimitotic agents binding to a specific site of tubulin, with the expectation of finding structures that could fit in it. These searches repeatedly retrieved frentizole, an approved nontoxic anti-inflammatory drug, thus indicating that it might behave as an antimitotic drug devoid of the undesired toxic effects. We also show that the usual repurposing approach to searching for targets of frentizole failed in most cases to find such a relationship. We synthesized frentizole and a series of analogs to assay them as antimitotic agents and found antiproliferative activity against HeLa tumor cells, inhibition of microtubule formation within cells, and arrest at the G_2_/M phases of the cell cycle, phenotypes that agree with binding to tubulin as the mechanism of action. The docking studies suggest binding at the colchicine site in different modes. These results support the repurposing of frentizole for cancer treatment, especially for glioblastoma.

## 1. Introduction

Cancer is considered responsible for approximately one in six deaths worldwide, taking its toll on up to 10 million people in 2020 and being a leading cause of mortality worldwide [1]. Cancer is a complex group of diseases characterized by the progressive transformation of normal human cells into neoplastic by the multistep acquisition of biological capabilities called the hallmarks of cancer [2]. Cancer therapeutics have targeted these traits to achieve outstanding advances over the last decades, with new therapeutic strategies, such as the monoclonal antibodies, immunotherapies, and cell-based immunotherapy (e.g., chimeric antigen receptor T therapy (CAR-T)), joining the traditional treatments of surgery, radiation, chemotherapy, targeted therapy, hormonal therapy, and immunotherapy [3]. Although the combined advances in cancer therapy, diagnosis, and genomic technologies are improving patients’ survival and quality of life [4,5], developing new drugs that overcome the main limitations of existing ones, that is, toxicity, limited effectiveness, and drug resistance, is essential. Despite the groundbreaking advances achieved in cancer biology, the discovery of new drugs is a lengthy (12 years, on average) and costly (estimated cost between USD 2 and 3 billion) process with a high intrinsic attrition rate (only around 13% of drugs that enter clinical trials achieve approval) [6]. The recognition that patients suffering from lethal cancers cannot wait the length of time required by the most time-demanding standard randomized phase III clinical trials has recently led to new regulatory frameworks. Priority reviews, accelerated approvals, fast-track approval, and breakthrough status have shortened the approval time of certain oncology drugs to a median of 7 years or 4.8 years for the fastest pathways [7,8]. For oncologic drugs not eligible for these accelerated tracks, as is the case for the more established chemotherapy options (e.g., cytotoxic, antimitotic, or targeted drugs), the repurposing (also called repositioning) of approved drugs for nononcologic indications is a promising strategy for accelerating drug discovery. Approved drugs that have already cleared the preclinical and clinical phases are safe and nontoxic (especially compared to oncology drugs) [9]. Recently, this approach has been applied even to the assay of multiple drug combinations, such as the combination of nine approved drugs with “well-tolerated” profiles that hypothetically target pathways associated with the recurrence of glioblastoma, an unmet clinical need, in the CUSP9 and the CUSP9* treatment protocols [10,11,12].

Drug repurposing is a drug discovery strategy based on the identification of new therapeutic indications for approved or investigational drugs. Drug repurposing usually begins with the identification of a candidate for the pursued indication (i.e., hypothesis generation), followed by the preclinical assessment of the expected effect, and finally the confirmation of efficacy in clinical trials [13]. The first step is the most critical, and both experimental and computational strategies have been applied to accomplish it. The experimental approaches usually rely on high throughput assays, with exemplary successes being the approval of the anticancer drug zidovudine as the first HIV drug [14] and thalidomide for refractory multiple myeloma based on its antiangiogenic properties [15]. The computational approaches rely on signature matching procedures, where unique characteristics (i.e., signatures) of a drug are compared to those of another drug or phenotype to find similar patterns and suggest drug-repurposing options. The signatures can be related to -omic data (i.e., proteomic, transcriptomic, or metabolomic) similarities between drug treatments and diseases or other drugs, chemical structures (i.e., structural similarity), adverse event profiles, molecular docking, retrospective clinical analysis, genetic associations, or pathway mappings. Signature matching based on chemical structures is grounded on the hypothesis that chemical similarities underlie shared biological activities. The process involves the comparison of a drug’s chemical features with those of drug networks binding to a common biological target to find new drug–target associations. The chemical features can be statistics-based cheminformatic parameters [16] or 2D structural similarities [17]. The pitfalls of these structural-similarity-based approaches are errors in chemical structures and biological effects beyond a structural relationship (for example, drugs binding to different sites or biological effects due to modified structures).

Tubulin inhibitors are antimitotic cytotoxic compounds with good clinical efficacy but with severe on-target toxicity causing peripheral neuropathy, neurotoxicity, stomatitis, bone marrow suppression, thromboembolic events, and weakness that altogether with poor pharmacokinetic profiles and the development of resistances limit their clinical application [18,19]. Tubulin is a heterodimer of α- and β-tubulin subunits that engages in highly regulated polymerization-depolymerization equilibria to assemble the dynamic microtubules of the cytoskeleton of eukaryotic cells. Microtubules and their dynamic behavior play essential roles in cell division through the formation and functioning of the mitotic spindle; the maintenance of cell shape; the intracellular trafficking of organelles, vesicles, and many other cellular cargoes; and more nonmitotic functions. Many antimitotic drugs interfere with the tubulin dynamic equilibria by binding to the many different drug-binding sites known. There are more than seven structurally characterized drug-binding sites in tubulin: the colchicine site, the Vinca alkaloids site, the taxanes site, the laulimalide/peloruside site, the eribulin site, and the recently discovered gatorbulin-1 site [20] in the β subunit and the pironetin-binding site in the α subunit [21,22,23]. Additional potential drug-binding sites in tubulin have recently been uncovered [24,25]. The most clinically successful antimitotic agents, the taxanes and the Vinca minor alkaloid drugs, are natural product derivatives of very complex structures that render them susceptible to multidrug resistance (MDR) efflux proteins and result in unfavorable pharmacokinetic properties and high toxicity profiles. Therefore, finding nontoxic inhibitors of tubulin is a promising strategy for the discovery of new cancer treatments. Colchicine-site ligands are structurally simpler compounds, quite often nonsusceptible to MDR, but still suffering from pharmacokinetic and toxicity liabilities. Their drug-like size makes them more favorable options for the search of structurally similar approved drugs that would, therefore, be expected to surmount the pharmacokinetic and toxicity liabilities and show the antimitotic activity that has rendered them one of the more successful anticancer therapies. This combination of a colchicine-site antimitotic agent with a low toxicity profile has recently led to the approval of tirbanibulin [26], the first colchicine-site ligand for the treatment of actinic keratosis and psoriasis [27]. The colchicine site of tubulin has three subpockets, called I, II, and III (or alternatively as their binding-partner ligand moieties B, A, and C) [28,29]. Very few ligands bind simultaneously to the three of them (BAC ligands), and those that do only accomplish a partial occupation. We, therefore, chose colchicine-site ligands that bind to only two of the subpockets. This reduction constrained the search to a more limited combination of structural features that, thus, enhanced the likelihood of finding positives within a library of drugs that had advanced through the first phases of clinical trials, considered to represent good nontoxic candidates for repurposing efforts. Recently, drug repurposing strategies have been applied to find potential drugs against COVID-19 and other viruses, finding ligands binding to the colchicine A and C subzones (AC) that showed a lower toxicity profile while affecting microtubule dynamics. This favorable profile led us to start our search by selecting approved drugs with structural similarity to structurally ascertained AC drugs, such as the benzimidazoles nocodazole and mebendazole, TN16, the pyridopyrazines N2G and N2K, plinabulin, the cyclohexanediones TUB015 and TUB075, and the benzothiazole (BZT) MI-181 (Supplementary Figure S2) [22,30].

**Figure 1:**
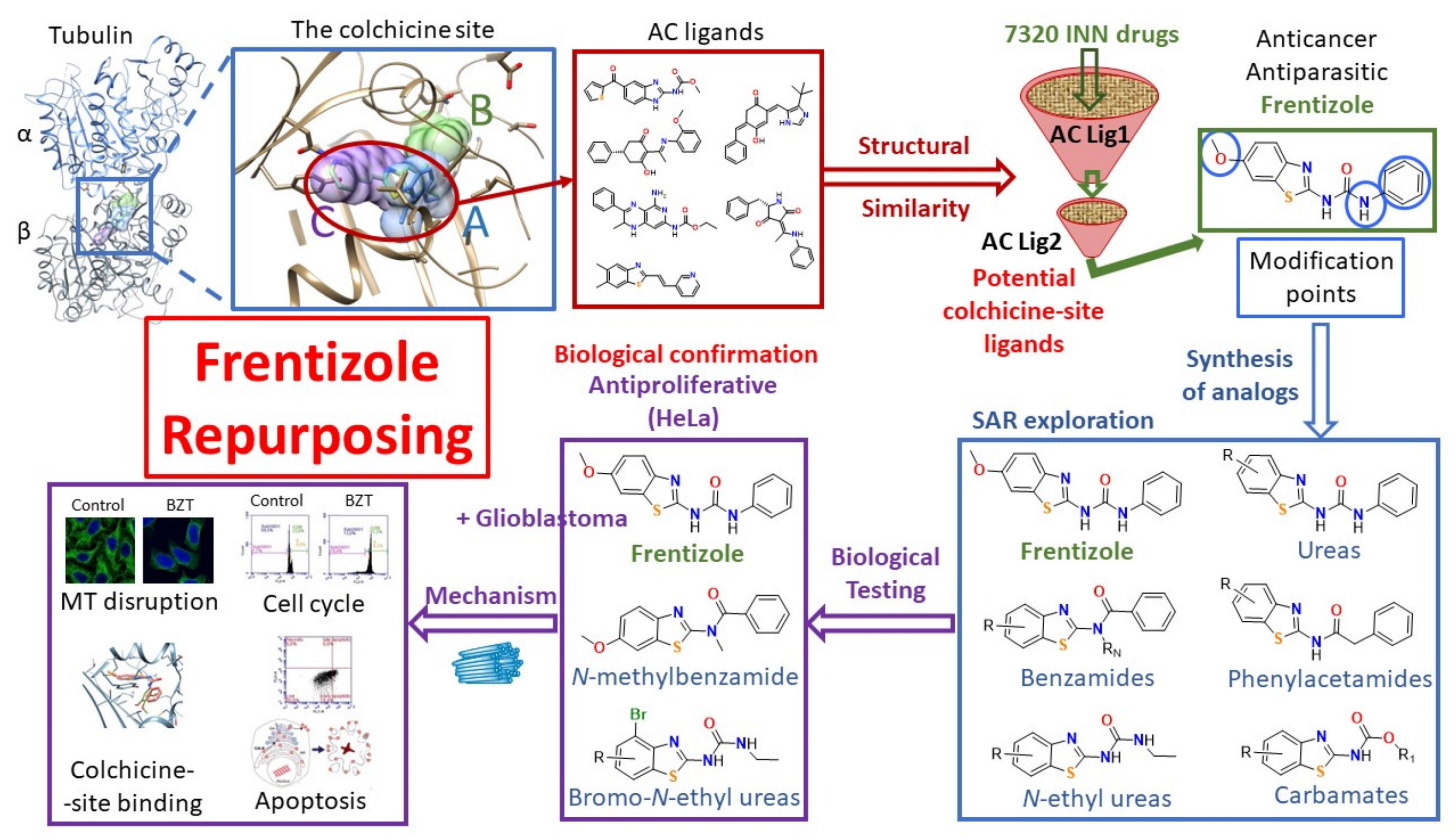
The pathway to the repurposing of the nontoxic immunomodulatory drug frentizole and its analogs as antimitotic drugs for cancer therapy.

Here (Figure 1), we describe our results in the search for approved drugs with clinical applications unrelated to the usual antitubulin drugs (i.e., nononcologic or antiparasitic) that might act as tubulin inhibitors, as they could show antitumor activity in the absence of the usual pharmacokinetic and toxicity liabilities of antitubulin drugs. We followed an unprecedented strategy, searching for the structural similarity of the approved drugs to several antimitotic agents binding to a specific site (i.e., the colchicine site) and subpocket in tubulin. Using known ligands with different scaffolds that bind to the same subsites and the application of diffuse structural associations, we had the expectation of finding structures divergent from the initial ones that might fit this particular binding site by satisfying its structural requirements in an unpredicted way. Our searches repeatedly retrieved frentizole, an approved nontoxic anti-inflammatory drug, thus suggesting that it might serve the aimed purpose. We then applied the usual inverse approach to drug repurposing to the search for targets of frentizole but, in most cases, they failed to find such a relationship, thus indicating that this strategy would have failed to find frentizole. We then synthesized frentizole and a series of analogs to explore the structure–activity relationships in this series and assayed them as potential cytotoxic agents. Frentizole and a handful of its derivatives showed antiproliferative activity against the HeLa tumor cell line, and frentizole also showed antiproliferative activity against the glioblastoma cell line U87 MG. We studied the mechanism of action of frentizole and the active derivatives: they inhibit microtubule formation within cells, arrest cells in the G_2_/M phases of the cell cycle, and induce apoptotic cell death, phenotypes that agree with an antitubulin mechanism of action. Docking studies suggest binding at the colchicine site in different binding modes. These results support the repurposing of frentizole for cancer treatment, especially for glioblastoma.

## 2. Results

### 2.1. Structural Search for Approved Drugs Similar to Colchicine—Site Antimitotics

**Figure 2:**
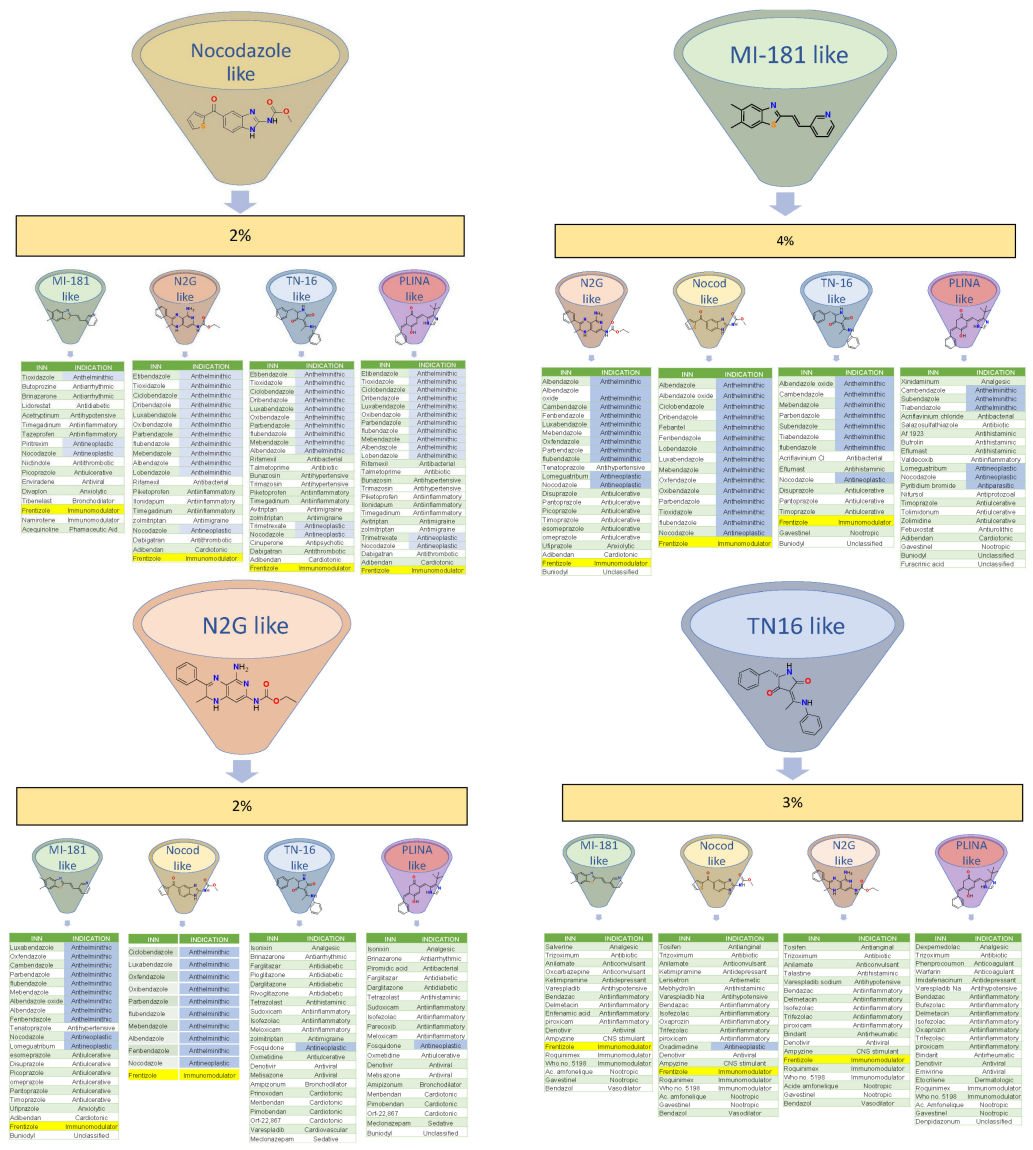
Graphical summary of the screening process using similarity to known A–C colchicine-site binding agents nocodazole, MI-181, N2G, and TN-16. The upper filter reduces the initial database, and the secondary filters are shown beneath. The table shows the names and indications of the filtered compounds ordered by indication. Antiparasitic and antineoplastic agents are highlighted with a blue background, and frentizole is highlighted with a yellow background.

Starting with a library of 7320 drugs with assigned International Nonproprietary Names (INN), we performed a recursive search for compounds similar to the AC colchicine-site ligands (Figure 2). To this end, an evaluation of the similarity to the reference compounds was performed using DataWarrior’s default descriptor FragFp [31], a substructure based binary fingerprint that relies on a dictionary of 512 predefined structure fragments, where heteroatoms have often been replaced by wildcards, thus allowing for heteroatom replacements. In the first similarity evaluation, an upper limit for the retrieved compounds was set to 6% of the original library. This limit allows for the retrieval of compounds like the first reference without compromising too much the search for similarities to the second one. Then, a similarity search is performed again on the first selected set with a different ligand, applying a cutoff of 10%, thus selecting a final set of fewer than 30 drugs. This two-step procedure allowed us to select compounds that match different AC ligands, as the colchicine AC site seems to be quite tolerant to binding different scaffolds. The two-step process, applying two different references, was expected to select the structural features underlying binding to the AC site but without restricting the search to compounds with too similar scaffolds. After performing the two-step selections for several reference combinations, the retrieved compounds of each were compared. The compounds selected using different searches would be more likely to satisfy the requirements for an AC ligand (Figure 2, Supplementary Figures S1–S4, and Supplementary Table S1) [32,33,34,35,36,37,38].

After performing the sequential similarity searches, frentizole, a nontoxic immunomodulatory drug that has been used to treat rheumatoid arthritis and systemic lupus erythematosus, appeared as a common result, revealing a structural similarity to structurally diverse colchicine-site ligands and, thus, suggesting that it could satisfy the structural requirements for binding to the AC subsite of the colchicine site and, therefore, have antimitotic activity. In an attempt to strengthen our hypothesis, we then proceeded to perform the inverse search by predicting potential targets for frentizole [39]. To this end, we used several target prediction tools (Supplementary Tables S2–S6): SuperPred [40], TargetHunter [41], ChemicalChecker [42], SwissTargetPrediction, and SwissSimilarity tools [43,44], and the Similarity ensemble approach (SEA) [16]. Only the last one retrieved tubulin as a target for frentizole, and, therefore, the relationship would have been missed using this reverse approach. Analyzing the results, one possible explanation is that the annotated tubulin ligands in the databases used by the above tools have colchicine-site ligands with mixed binding-site subpocket occupation and, therefore, the similarity to the AC ligands stays unnoticed. 

### 2.2. Synthesis of Frentizole and Analogs

**Scheme 1:**
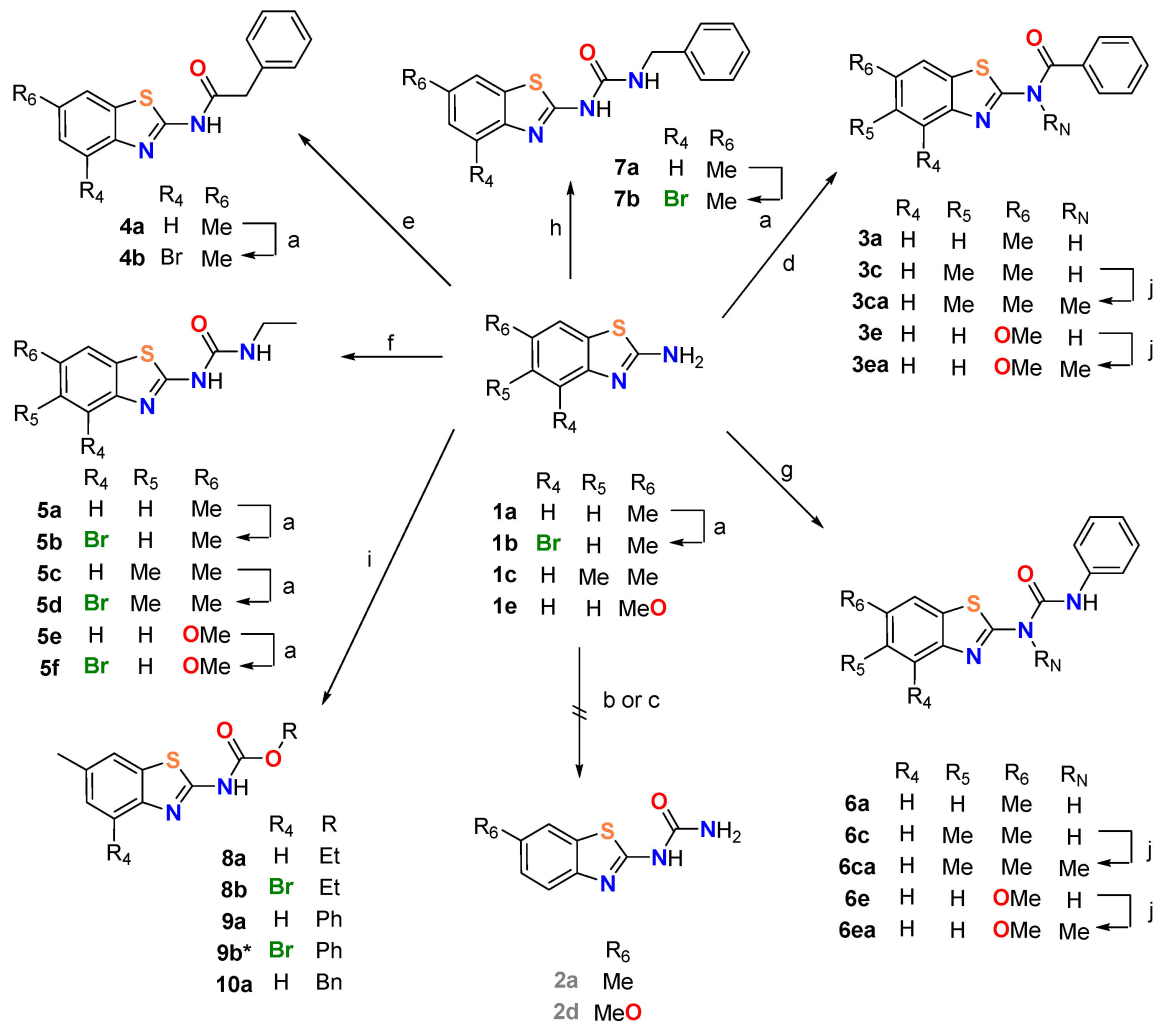
Synthesis of frentizole analogs. Reagents and conditions: (**a**) NBS, CH_2_Cl_2_, rt, 24 h; (**b**) **1a**, ClSO_2_NCO, CH_2_Cl_2_, rt, 24 h; (**c**) **1e**, KOCN, 1:1 H_2_O:AcOH, 40 °C, 24 h; (**d**) PhCOCl, Et_3_N, CH_2_Cl_2_, rt, 24 h; (**e**) PhCH_2_COCl, Et_3_N, CH_2_Cl_2_, rt, 24 h; (**f**) EtNCO, CH_2_Cl_2_, rt to reflux, 24–72 h; (**g**) PhNCO, CH_2_Cl_2_, rt to reflux, 24–72 h; (**h**) PhCH_2_NCO, CH_2_Cl_2_, rt to reflux, 24–72 h; (**i**) EtOCOCl (**8**), PhOCOCl (**9**), or PhCH_2_OCOCl (**10**), Et_3_N, CH_2_Cl_2_, rt, 24 h; (**j**) MeI, KOH, CH_3_CN, rt, 24 h. * Compound **9b** has a second phenyl carbonate unit attached to the nitrogen. Compounds **2a**, **2d** [45], **3a** [46], **3e** [47], **6a** [48], **6e** (frentizole), **8a**, and **9a** [49] have been previously described.

The synthesis of frentizole (**6e**) and the structural variants (**2**–**10**) was performed as shown in Scheme 1. 2-Aminobenzotiazoles **1a**, **1c**, and **1e** are commercial, and the brominated derivatives **1b** and **1d** were obtained by bromination with NBS. The bromination position was ascertained via nOe experiments between the aromatic protons and the substituents at position 6. We unsuccessfully attempted the synthesis of the unsubstituted ureas **2** by the reaction of the 2-amino benzothiazoles with chlorosulfonyl isocyanate or potassium cyanate in different conditions. Benzoyl derivatives **3** were prepared by reaction of the 2-aminobenzotiazoles with benzoyl chloride, while the phenylacetamides **4** were prepared by reaction with 2-phenylacetylchloride. Methylation of the amide nitrogen was achieved by reaction with methyl iodide in the presence of KOH as a base to afford **3ca** and **3ea**. Substituted ureas **5**, **6**, and **7** were prepared by reaction of the 2-aminobenzothiazoles with ethyl (**5**), phenyl (**6**), and benzyl (**7**) isocyanates. The brominated derivatives **5b**, **5d**, **5f**, and **7b** were prepared by treatment with NBS, and the methylations were carried out with methyl iodide and KOH, resulting in **6ca** and **6ea**. The carbamates **8**, **9**, and **10** were prepared by reaction with the corresponding chloroformates.

### 2.3. Biology

#### 2.3.1. Antiproliferative Activity

The cell proliferation inhibitory activity of frentizole and the synthesized analogs **2**–**10** against representative human cancer cell lines was assayed using the MTT method (Table 1). For an initial screening of the active vs inactive compounds, the cell viability 72 h after the drug treatments at a threshold concentration of 10 μM of compound was measured in triplicate and compared with the untreated controls. For those compounds inhibiting cell proliferation by more than 40% with respect to the untreated controls at the threshold concentration, the IC_50_ values were calculated by measuring the proliferation inhibition in a range of concentrations from 100 to 0.001 μM and adjusting the resulting curves to sigmoidal dose–response curves. The selected cell lines were HeLa (cervix epithelioid carcinoma cells), one of the most frequent kinds of cancer, with good sensitivity against antimitotic agents, and the glioblastoma cell lines U87MG and A172. To study the selectivity of the compounds toward cancer cells versus nontumor cells, the compounds were also evaluated against the nontumorigenic cell line HEK-293 (human embryonic kidney cells) and the murine macrophage cell line J774. Sulfonamide ABT-751, as a representative of the colchicine-site ligands, and the first-line drug for the treatment of glioblastoma multiforme temozolomide (TMZ) were used as reference compounds.

Most compounds did not show antiproliferative activity at concentrations below 10 μM, as would have been anticipated for a series of analogs of frentizole, which is described as a nontoxic anti-inflammatory drug. Satisfyingly and according to the predicted similarity to antimitotic agents, three chemical types of 2-aminobenzothiazole (BZT) derivatives showed significant activity against HeLa cells: benzamide **3ea**, brominated ethyl ureas **5b**, **5d**, and **5f**; and frentizole (**6e**) itself, thus confirming the prediction based on the structural similarity with several colchicine-site ligands. Interestingly, all active compounds were in the low micromolar or submicromolar range against HeLa cells, while most other representatives of the chemotypes were not active. The only group with several actives was the ethyl ureas, which also required a bromine substituent at position 4 of the benzothiazole ring. This modification was, however, not sufficient to render other structural classes active, as can be seen for the brominated carbamates **8b**, **9b**, and **10b** or the benzylurea **7b**. For the brominated ethyl urea series, similar antiproliferative potencies are observed for different substitutions on the phenyl ring of the BZT moiety in the descending order of 5-methyl > 6-methoxy > 5,6-dimethyl. None of the brominated ethyl ureas were active against the glioblastoma cell lines U172 and U87MG, which were less sensitive, in general, to the BZT analogs than the HeLa cells. Interestingly, frentizole was, again, the only compound in the whole series to show activity below the threshold concentration against glioblastoma cell line U87MG, although with a higher IC_50_ value of 7.33 μM. We recently showed that BZT-based inhibitors of tubulin polymerization have antiproliferative activity against glioblastoma cells, which are known to be especially sensitive to tubulin inhibitors [50]. In the phenyl urea series, the only substituent tolerated at the BZT moiety is the methoxy group present in frentizole, and methylation of the urea is also detrimental to the activity, as shown by the methylation of frentizole (**6e**). The opposite situation is observed for the benzamide class, where the only active compound is the methylated derivative **3ea**. For this class, as in the frentizole-like phenyl ureas, the methoxy substituent on the BZT is the only one accepted.

#### 2.3.2. Effect of the Compounds on the Cell Cycle

**Figure 3:**
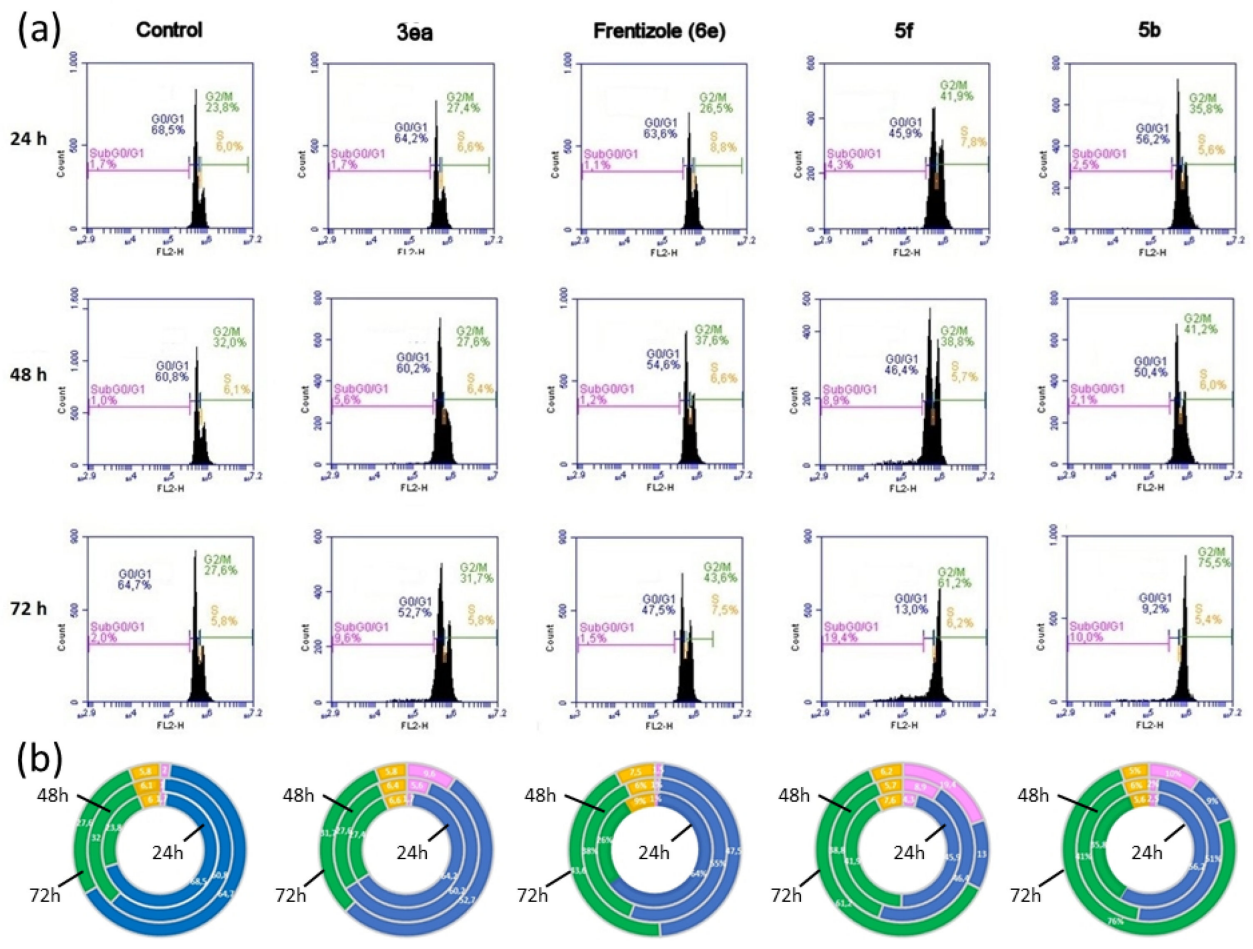
(**a**) Histograms of the cell cycle distribution of HeLa cells 24, 48, or 72 h after treatment with compounds **3ea,** frentizole (**6e**), **5f**, and **5b** at 2 μM. Untreated control cells were run in parallel. The bars of different colors indicate the positions of the subG_0_/G_1_ (pink), G_0_/G_1_ (blue), S (orange), and G_2_/M (green) regions. (**b**) Ring graph representations of the cell cycle region percentages. The concentric rings represent, from inside to outside, the 24 h, 48 h, and 72 h timepoints, with the segments colored as the histograms.

The observed antiproliferative activity against the HeLa cells of frentizole and of some of the synthesized analogs (Table 1) led us to study the effect of the most potent compounds (**3ea**, **5b**, **5f**, and frentizole (**6e**)) on the cell populations’ distribution along the cell cycle (Figure 3). Compound **5d** showed a very limited solubility and was not further studied. To study the effects of the compounds on the cell cycle, cells were incubated for different times with the compounds and, thereafter, their DNA was stained using propidium iodide (PI) to allow for quantification by flow cytometry. Time-course analyses of the cell cycle histograms at 24 h, 48, and 72 h showed a common general pattern that develops differently depending on the compound. All compounds showed a progressive decrease in the resting G_0_/G_1_ populations, with a concomitant increase in the G_2_/M and/or subG_0_/G_1_ populations. The brominated ethyl BZTs (**5b** and **5f**) were faster to show the subG_0_/G_1_ decrease, at 24 h, followed by an apparent stabilization of the G_0_/G_1_ population at 48 h, which abruptly fell at the 72 h time point. The two compounds differ slightly in the rates of increase in the G_2_/M and/or subG_0_/G_1_ populations, with **5b** showing an arrest at G_2_/M in the 24 to 48 h period that increases at the 72 h timepoint with the enlargement of the subG_0_/G_1_ population, while for **5f** the increase in the subG_0_/G_1_ population progressively occurs at the 48 h to 72 h timepoints. Compound **3ea** has the weakest effect on the cell populations, with a progressive increase in the subG_0_/G_1_ population after 48 h. Frentizole (**6e**) elicits a progressive accumulation of cells in the G_2_/M population, without the abrupt change seen for the ethyl ureas. Also, no increase in the subG_0_/G_1_ population was observed after treatments with frentizole, even at 72 h.

#### 2.3.3. Cell Death Mechanism Studies

**Figure 4:**
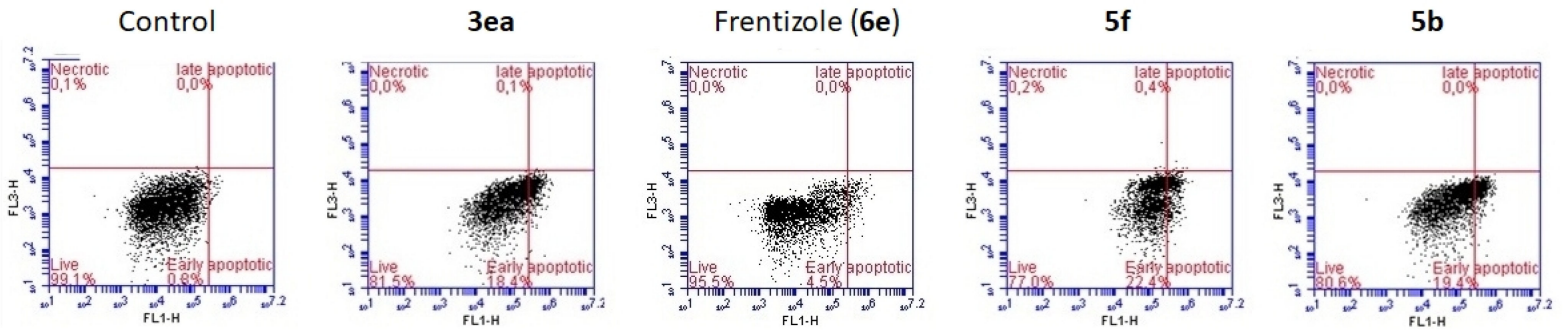
Annexin V-FITC (ordinates) and propidium iodide (PI) (abscissas) profiles of HeLa cells 72 h after treatment with compounds **3ea**, frentizole (**6e**), **5f**, and **5b** at 2 μM. Untreated cells were used as negative controls.

The treatment of HeLa cells with frentizole and the synthesized analogs resulted in the appearance of cells with low DNA content, the subG_0_/G_1_ populations that are usually assigned to apoptotic cells. To further characterize the mechanism of action of the compounds, dual-channel flow cytometry studies (Figure 4) were carried out on cells in the absence (i.e., negative control) or after 72 h treatments with the compounds, as this was the timepoint at which the subG_0_/G_1_ populations started to accumulate in the cell cycle analysis. Fluorescein-isothiocyanate-labeled Annexin V (FITC-AnV) and propidium iodide (PI) were used for the double staining. PI staining indicates cell permeation due to some kind of membrane disintegration, as it occurs in late apoptotic or necrotic cells, whereas cell staining with fluorescent AnV reflects the translocation of the phosphatidylserine from the inner to the outer cell membrane, which takes place early in the apoptotic response. Accordingly, the cells are classified depending on their staining by the two dyes as viable (PI−, AnV−), early apoptotic (PI−, AnV+), late apoptotic or secondary necrotic (PI+, AnV+), or necrotic (PI+, AnV−).

Consistent with the results of the cell cycle histograms, we observed moderate levels of apoptotic cells for the treatments, but in all cases, the apoptotic levels are well above those of untreated HeLa cells. In good agreement with the cell cycle profiles, the apoptotic response was higher after 72 h of incubation with **3ea**, **5d**, and **5f** than with frentizole. The apoptotic cells for all of them belong to the early apoptosis class (AnV+, PI−), with almost no late apoptotic cells (PI+, AnV+). There is a slightly higher percentage of apoptotic cells in the double-stained experiments compared with the single PI-stained cell cycle histograms. This could be due to a more precise cell classification in the latter, as early apoptotic cells would not be labeled as apoptotic (subG_0_/G_1_) until the DNA fragmentation process starts but as belonging to the other populations based only on their DNA content. 

#### 2.3.4. Effects on Cellular Microtubules

The arrest of the cell cycle at the G_2_/M phase is a signature of antimitotic drugs, such as those taken as models at the onset. The fact that frentizole and its derivatives arrest the cell cycle in G_2_/M is also consistent with interference with tubulin polymerization. Therefore, we set out to study the effect of the drugs on the microtubules of treated cells after 24 h, a time considered enough to alter tubulin polymerization in cells without inducing significant cell death based on the time course results of the cell cycle analysis. 

**Figure 5:**
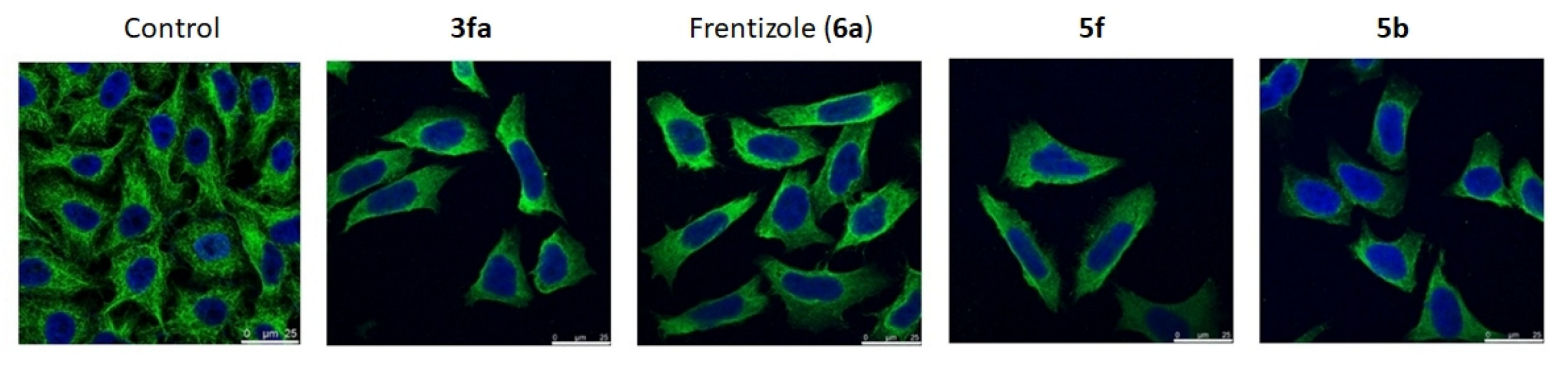
Effects of the treatment for 24 h with compounds **3ea,** frentizole (**6e**), **5f**, and **5b** at 2 μM on the microtubule network of HeLa cells as observed by confocal microscopy, with α-tubulin (microtubules) stained in green and nuclei stained in blue. The negative control are untreated cells. Scale bars: 25 μm.

Therefore, we studied the effect of the compounds after 24 h of treatment on the microtubule network of HeLa cells by immunofluorescence confocal microscopy (Figure 5) through α-tubulin and nuclei labeling. Untreated HeLa cells used as negative controls exhibit round, blue-stained nuclei, with a well-defined cellular body covered by a hairy microtubule network formed by distinct microtubule fibrils. After treatment with the compounds, HeLa cells show elongated cell shapes and nuclei, with a severe microtubule network disruption observed in all cases as undefined green masses lacking structured filaments. These morphological effects are typical of tubulin inhibitors. Again, the effects are more pronounced for **5b** than for **3ea**, frentizole, and **5f**.

### 2.4. Computational Studies

#### 2.4.1. DFT

Ureas can adopt different configurations by rotation of the amide bonds, which for unsymmetrically substituted ones result in four major possibilities, namely, *cis*–*cis*, *cis*–*trans*, *trans*–*cis*, and *trans*–*trans* (Supplementary Figures S5–S7). The rotational energy barrier for ureas is lower than for amides due to the competing conjugation of the two amide bonds. Therefore, faster configurational transitions are expected, thus resulting in conformational-like equilibria [51]. This implies that they can exchange during the biological assays and that the active conformations do not necessarily need to be the same as in solution or in the solid state if the energy difference is not large or the preferred conformation varies with the environment. Diaryl ureas in the solid state show a strong preference for the more extended *trans*–*trans* configuration [52]. In solution, they are mostly considered to adopt the same *trans*–*trans* configuration as in the solid state, but recent studies have shown that more dynamic conformational equilibria are in place, especially when intramolecular interactions are possible, as it is here the case due to the hydrogen bond acceptor nature of the benzothiazole nitrogen and the sigma hole bonding capability of the sulfur atom [53]. Alkylation of the urea nitrogen also changes the conformational preferences observed, in a similar way as it does with amides, that switch from preferred *trans* configurations to *cis* ones [54]. Because of this conformational variability, we studied the structure and the conformational preferences of the ethyl benzothiazole ureas, diaryl ureas, and the amides using DFT calculations, focusing on the energetic differences among the possible dispositions of the substituents of the urea substituents, as they could potentially condition binding to tubulin and/or the occupation of the colchicine domain subsites. AB-binding ligands adopt folded configurations with the two aryl rings close in space, consistent with at least one *cis* urea bond, whereas AC binding requires a more extended arrangement with the two aromatic rings stretching toward opposite ends, as would be achieved by all-*trans* compounds. 

The ethyl BZT ureas (e.g., **5b** and **5d**) preferred conformations (Supplementary Figure S5) in vacuum that had urea moieties in *trans*–*cis* configurations (considering the carbonyl oxygen to hydrogen arrangements), where the NH distant from the BZT makes an intramolecular hydrogen bond to the BZT’s nitrogen. In water, this configuration is similar in energy to the all-*trans* configuration with the two NH vectors pointing in the same direction. Interestingly, this latter configuration is very similar to the nocodazole structure when in complex with the AC zone of tubulin, with the two NH vectors perfectly overlapping. This suggests that these compounds might well adapt to this binding site.

Methylated benzamide **3ea** also has a very similar shape to nocodazole in its more stable *trans* configuration [36], but it is more similar to MI-181 [37], as the methylated amide is more similar to the double bond than to the highly polar NH bonds of the ureas (Supplementary Figure S6).

Frentizole has a preferred *trans*–*cis* configuration as that described for the ethyl ureas, with the all-*trans* one being less stable (Supplementary Figure S7). This configuration is the one observed in X-ray crystals, adopting a bent disposition, probably due to optimization of the crystal packing forces [52]. Again, the superposition of the NH bonds onto those of nocodazole results in an excellent overall fit of the two molecules. These results suggest that the active compounds can adopt configurations compatible with binding at the colchicine site of tubulin, engaging with the AC subpocket similarly to nocodazole but also with the AB subpocket in a similar way to known BZT-based colchicine-site binders. The alternative configurations found that form intramolecular hydrogen bonds might also provide an explanation for the membrane crossing ability, as formation of the intramolecular hydrogen bond, favored in low polar environments, is less polar and might allow membrane crossing.

#### 2.4.2. Docking Studies

**Figure 6:**
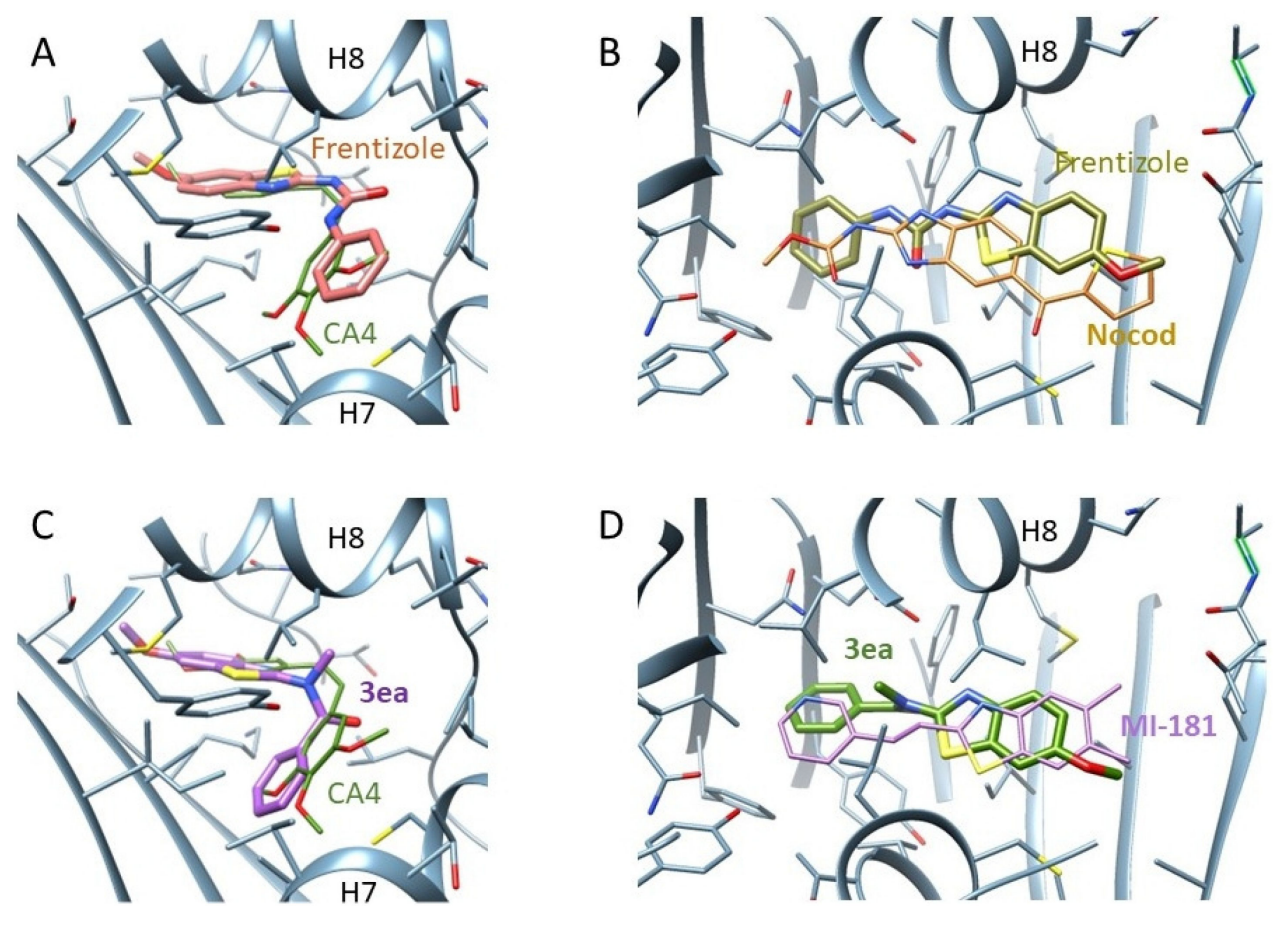
(**A**) Docking pose for the *cis*–*trans* isomer of frentizole (carbons in pink) in the AB subpockets, with combretastatin A-4 (CA4) shown in green. (**B**) Docking pose for the *all*-*trans* isomer of frentizole (carbons in olive green) in the AC subpockets, with nocodazole (Nocod) shown in orange. (**C**) Docking pose for the *cis* isomer of *N*-methyl benzamide **3ea** (carbons in green) in the AB subpockets, with combretastatin A-4 (CA4) shown in green. (**D**) Docking pose for the *trans* isomer of *N*-methyl benzamide **3ea** (carbons in green) in the AC subpockets, with MI-181 shown in light magenta.

The binding modes of the active compounds (Figure 6) within the colchicine site of tubulin were studied via docking experiments. As shown by the many available X-ray crystal structures of complexes with structurally diverse ligands, the colchicine site is flexible enough to allocate ligands with different scaffolds and substituents in the three binding subpockets (A, B, and C), even for structurally similar ligands [22,30]. We accounted for this receptor flexibility using 145 tubulin structures representative of the different configurations of the X-ray crystal structures of tubulin in complexes with different colchicine-site ligands. The 145 colchicine-site structures used in the docking experiments came from adding the 112 X-ray crystal structures of the complexes of tubulin with the different representative ligands within the colchicine site selected from the pdb, excluding the waters [55], plus 27 sites including nearby water molecules that make bridges between the ligands and the proteins up to a total of 139 structures. We also used six additional structures from a molecular dynamics simulation previously obtained [56]. Docking calculations were run with two docking software programs (PLANTS [57] and AutoDock 4.2 [58]) that apply different scoring functions, keeping the proteins rigid (ensemble docking procedure), while the ligands were flexible. The docking programs do not rotate amide bonds, and, therefore, we built all possible amide isomers (see Section 2.4.1 and Supplementary Figure S5) and ran independent docking calculations for all of them. The selected binding mode for each ligand was chosen by comparing the docking results for all possible configurations.

The docking results for the virtual ligands within the protein sites’ ensemble select the best fit combinations of ligand and receptor pairs, thus efficiently exploring the accessible interactional space. For every docked ligand, the binding pose was assigned to the common pair of poses with the best energy scores for the two docking programs. The structural comparison was based on automated geometrical comparisons and visual inspection. Subpocket occupancy assignments for each ligand pose were determined by measuring their lowest distances to the subpocket geometrical centers as defined by the pharmacophores derived from the X-ray structures of the colchicine-site ligands in complex with tubulin [55]. We converted the individual scores to relative scales ranking from 0 (worse) to 1 (best), and calculated the Z-scores to allow for the comparison of the scores of the two programs. For each considered ligand or ligand configuration, we assigned as the consensus binding-mode the pairs of poses with similar binding modes, as assessed by site occupation and visual inspection, and having the best combination of Z-values for the two programs (Supplementary Table S6) [59]. To assist in the comparison of the different binding modes and ligand dispositions, the selected poses were subjected to molecular dynamics calculations and the complexes re-scored with molecular mechanics–generalized Born scoring (MMGBSA) schemes (Supplementary Table S6) [60]. Control experiments correctly retrieved the experimental X-ray binding modes of representative colchicine-site ligands in complex with tubulin poses as described, thus validating the applied methodology [61].

The docking procedure applied resulted in the placement of the BZT ligands in all the possible binding subpocket combinations, thus showing that the sampling protocol explored rather exhaustively the colchicine site. For each protein structure, a differential but not exclusive binding-mode selection was often observed that matched the configuration of the X-ray ligand (i.e., proteins with AB ligands tend to select for AB poses but also find AC and other alternative modes). This is a consequence of the fitting of the protein pocket to its native ligand, but alternative binding modes are not forbidden, as the other pockets do not completely collapse if unoccupied. A clear overall favoring of the AB- or AC-binding modes for the ensemble of ligands is seen. For every ligand, the selected binding modes vary depending on the configuration considered, as they enforce geometries that best fit one or another subpocket combination. As a result of the many possible arrangements that may occur, the BZT ring can be found in any of the subpockets in the different ligands and protein combinations. This is in good agreement with previous studies on BZT ligands whose binding modes have been experimentally determined, showing that the BZT adapts to the colchicine subpockets quite promiscuously, with the rest of the molecule apparently having a larger impact on the binding modes. Thus, the *E* alkene MI-181 places the BZT ring within the A subpocket and the 3-pyridyl ring in the C-zone (pdbID: 4YJ2) [37]; the *Z* olefins SBTub2 (pdbID: 6ZWC) [62] and SBTub2M (pdbID: 7Z01) [63] place the BZT rings within the B pocket and the trimethoxyphenyl rings in their usual A zone; the *Z* olefin SBTub3 places the BZT within the A pocket and the 3-hydroxy-4-methoxyphenyl ring in its usual B zone (pdbID: 6ZWB) [62]; and, finally, the *Z* olefin SBTubA4 places the BZT with three methoxy substituents that render it equivalent to a trimethoxyphenyl ring within the A pocket and the 3-hydroxy-4-methoxyphenyl ring in its usual B zone (pdbID: 7Z01) [63].

The occupation of the B and A zones of the colchicine site requires a relative disposition of the binding moieties in a V-shaped arrangement such as that of a *Z* stilbene, for instance, combretastatin A-4, whereas the occupation of the A and C zones requires a more elongated geometry, such as that adopted by an *E* stilbene (e.g., MI-181). As shown by the DFT calculations, the amides and the ureas can adopt both types of geometrical arrangements depending on their configurations and, therefore, might bind differently to tubulin, both in terms of geometry and of binding pocket occupation. As the docking programs employed are not able to interconvert the amide and urea configurations, we have run separate docking calculations for all the possible configurations of each ligand and later combined the results to try to establish their binding modes. Thus, after the processing of the dockings results of the individual configurations for each ligand, they are combined and compared to each other using their energy scorings from the two docking programs, the MMGBSA energies, and their relative energies from the DFT calculations. 

The brominated ethyl ureas are smaller than the usual colchicine-site ligands and, therefore, might bind just one of the pockets and partially a second one. Smaller sizes are usually associated with lower binding affinities, and this might be the reason for the need of the bromine atom: to gain size and increase the number of interactions to make the binding efficient. Nonetheless, they show high cytotoxic potency and tubulin polymerization inhibitory activity within the cells such that their binding seems to be effective. For the methyl BZT ethyl urea, the preferred binding mode is the occupation of the B zone by the BZT ring, which results in carbonyl–π-type interactions with the side chain of Asn258β (S8). The urea configurations dispose, in different geometries, the ethyl urea moieties that can then occupy different locations within the A zone, only partially filling it. The larger methoxy BZT ethyl urea results in a lower preference for binding at the AB subpockets, as the methoxy benzothiazole can also fill the larger A site and protrude the ethyl urea toward the C zone, in a similar binding mode as that of nocodazole and MI-181. This requires an extended disposition and, thus, prevails in the all-*trans* urea configuration. Overall, the apparently preferred binding mode is with the BZT ring occupying the B zone, with the ethyl ureas projecting toward the A pocket.

The *N*-methylated benzamide can adopt two amide configurations, and rotations about the exocyclic amine bond result in four different configurations, with a preference for the *trans* (Me to C=O) disposition, as shown by the DFT calculations (Supplementary Figures S5–S7). The *trans* isomer adopts bent dispositions similar to those of BZT ligands occupying the AB zones [22,30,62,63], whereas the *cis* ones are more extended and similar to nocodazole [36] or MI-181 [37] when binding to the AC zones. Accordingly, the docking results place the *cis* isomer within the AB subpockets and the *trans* isomer within the AC ones. In the first instance, the BZT ring is located in the B zone and the phenyl ring in the region between the A the C zones, as previously described by us for related ureas [50]. For the *trans* isomer, the BZT is allocated to the A zone, similar to MI-181 [36], and the phenyl ring toward the C zone. The energy differences of the docking programs seem to favor the AC-binding mode, but the MMGBSA scores clear favor the BA-binding mode with the BZT ring in the B zone. The observation that the unmethylated amide is not active suggests that methylation might be a requirement for occupying the AB zones, which are not sampled by the amide, as its preferred configuration is the *trans*.

Frentizole has four possible configurations of the two amide bonds, plus the two tautomeric structures with an exocyclic imine in position 2 of the BZT moiety. The configurations with *cis* amide (O=C to HN) arrangements adopt bent dispositions and occupy the B and A zones, with the methoxybenzothiazole occupying the B zone in a very similar disposition as the 3-hydroxy-4-methoxyphenyl ring of combretastatin A4. The second amide configuration results in a different placement of the phenyl ring at the opposite end of the molecule, with the *cis* isomer placing the Ph ring within the A zone, packing the phenyl ring against Leu248β (L7), Lys352β (S9), and Ala354β (S9), whereas in the *trans* isomer it is located between the A and C zones, contacting Leu255β (H8) and Cis341β (H7). These later poses were the best scored, as they result in an intramolecular hydrogen bond between the second amide NH and the benzothiazole nitrogen, while the other HN vector projects toward the hydroxyl group of Thr179α. The urea with a *trans*–*cis* configuration places the aromatic rings in a similar disposition, but the NH vectors are differently arranged within the pocket. On the other hand, the all-*trans* configuration adopts a more linear disposition and bind to the A and C zones. The urea NH vectors arrange very similarly to those of nocodazole, as described in the DFT calculations, with the methoxy group aligned with that of the trimethoxyphenyl ring of combretastatin A-4. The MMGBSA score these conformations worse than for the *cis*–*trans* configuration in the AB zones. The tautomers with the exocyclic imine also show a preference for the AB-binding mode. 

Selected examples of the consensus binding poses for frentizole and *N*-methyl benzamide **3ea** in different configurations and binding modes are shown in Figure 6. 

The applied ensemble docking approach selects the protein structures that provide a better fit for each docking assay, thus informing on the best complementary sites to the assayed ligands’ configurations. For the AB occupying poses, the pdb IDs most frequently retrieved were 5JVD, with a methyl chalcone (2*E*)-3-(3-hydroxy-4-methoxyphenyl)-1-(7-methoxy-2*H*-1,3-benzodioxol-5-yl)-2-methylprop-2-en-1-one; 5H7O, with a 2-(1*H*-indol-4-yl)-4-(3,4,5-trimethoxyphenyl)-1*H*-imidazo [*4*,*5-c*]pyridine ligand; and 6D88 with a (2-(1*H*-indol-3-yl)-1*H*-imidazol-4-yl)(8-methoxybenzo[*b*][*1*,*4*]dioxin-6-yl)methanone. All of these ligands are structurally similar to CA4. However, on many occasions, different pdb IDs were retrieved, almost all of them carrying AB ligands because of the different shapes of the sampled ligand configurations. For the AC-binding poses, the most retrieved pdb IDs were 5S4Q and 7EMJ, both of them carrying AC ligands: 4-(4-methylthiazole-5-carbonyl)piperazin-2-one and the natural product barbigerone (8,8-dimethyl-3-(2,4,5-trimethoxyphenyl)-4*H*,8*H*-pyrano [*2*,*3-f*]chromen-4-one). These two ligands are quite different in size, which suggests that the binding pockets do not collapse in the unoccupied regions, thus allowing for the docking of ligands of different sizes.

The pdb IDs (i.e., protein structures) of every pair of consensus poses are usually different for AutoDock and PLANTS, probably as a result of their different scoring functions. This also results in differences in the binding poses, even if they are recognizably similar which allows for them to be selected for the consensus pose. These results sustain the application of the docking experiments of the ensemble docking strategies with as many protein structures as possible and validate the application of the consensus scoring approaches that allowed us to rationale the presumed binding modes.

## 3. Discussion

Drug repurposing for antimitotic effects is a promising strategy for the development of antitumor drugs, as approved drugs usually lack the high toxicity profile of conventional antimitotic agents, which are one of the most efficient but toxic cancer treatments [7,18]. Tubulin is one of the main targets of antimitotic agents and of vascular disrupting agents (VDAs), but its most often applied drugs (i.e., taxanes and Vinca alkaloids derivatives) are highly toxic and suffer from tumor resistance via drug efflux proteins associated with multidrug resistance phenotypes (MDR) [19,22]. Drug repurposing focusing on alternative binding sites in tubulin, such as the colchicine site might, therefore, be a promising alternative. This strategy of looking for approved drugs that might bind to a particular site or subsite combination of a target based on diffuse structural similarity to structurally characterized binders has not been previously used, as search strategies do not take into account similar binding modes but the similarity of effects at a higher level [42]. As a result, they often pick candidates that might elicit their effects through binding to different sites or even different targets. The choice of the colchicine site was due to the smaller size of the colchicine-site ligands compared with taxanes and Vinca alkaloids, which make them less susceptible to MDR proteins [22]. Particularly, colchicine-site ligands that bind to the AC subpockets have a higher polarity than ligands that bind to other regions of the colchicine domain, which might allow for finding more drug-like candidates without the usual high lipophilicity of colchicine-site ligands, which makes them poorly soluble and causes clinical failures. Along with this reasoning, the recent approval of a nontoxic colchicine-site binder reinforces this hypothesis [26,27].

The search for approved drugs potentially binding to the AC subpockets of the colchicine site started by selecting structurally characterized binders with different scaffolds [22,25,30]. Finding approved drugs with diffuse similarity to several of these reference molecules should select compounds with scaffolds not related to the ones represented by the references, as they bind to the same subpockets even if they lack recognizable commonalities. Satisfyingly, the structural search of approved drugs with similarity to the references resulted in the repeated selection of frentizole, a nontoxic immunosuppressive drug, altogether with known tubulin-site binders, such as antiparasitic and antiproliferative compounds. Frentizole does not share a common scaffold with any of the model compounds nor has similar moieties. However, the benzothiazole ring of frentizole is also present in colchicine-site ligands binding to different subpocket combinations depending on the exact structure [37,62,63]. The repeated finding of frentizole in the search results and the presence of BZT in the known colchicine-site binders firmly suggest that frentizole might, in fact, behave as an antimitotic agent through binding to tubulin at the colchicine site. We then proceeded to perform the search for a new drug indication for frentizole in the usual way of searching for similarities of frentizole to biologically active compounds present in public databases, but we only retrieved the tubulin binding profile once, even though the databases contain the reference compounds and the benzothiazole ligands mentioned. It might be the case that the compounds in the database bind tubulin at different subpockets, thus making the recognition of the structural features that allow for binding at specific protein subpocket combinations more difficult.

With the aim of testing the hypothesis that frentizole is a tubulin inhibitor, we synthesized frentizole and a series of analogs and evaluated them as antiproliferative agents against HeLa human cancer cells. Small structural changes can dramatically affect the potency of antiproliferative compounds, and colchicine-site binders are not an exception [22,24,28,50]. Therefore testing only a single compound (frentizole) might fail to uncover the desired activity. Assaying a series of structurally related compounds would increase the likelihood of finding a hit, as well as allow us to explore the structure–activity relationship for this family of benzothiazole derivatives and establish the structural elements that are essential for the activity. These elements, in turn, would assist in the analysis of the potential binding modes to tubulin of the active compounds. In agreement with the predictions, frentizole showed antiproliferative activity against HeLa cells with an IC_50_ value in the low micromolar range, while other closely related BZT ureas with modifications of the BZT ring or with methylated ureas did not. This suggests that the methoxy substituent is important for the activity in the phenyl urea series (compounds **6**). The methylation of the urea at the more distal to the BZT ring nitrogen or its substitution by an oxygen atom (carbamate analogs **8**–**10**) also abolished the activity, thus suggesting that a free distal NH group is important for the activity of frentizole. On the other hand, complete removal of this NH group to result in a benzamide (compounds **3**) only yielded an active compound if the amide group (equivalent to the proximal NH urea group of frentizole) was methylated (**3ea**). The methyl derivative preferentially adopts a *cisoid* configuration for the amide bond, thus changing its geometry and consequent binding subpocket preferences. The replacement of the phenyl ring on the distal to the BZT ring urea nitrogen of frentizole by an ethyl group did retain the activity, but only if the BZT ring was enlarged with a bromine atom. This occurred not only for the methoxy substituent on the BZT ring but also for other substitutions on the BZT ring, which is different from what was seen for the phenyl ureas. These results suggest that the replacement of the phenyl ring by the smaller ethyl groups requires a compensating increase in volume on the BZT moiety, and the observed differences might result from different binding modes. As anticipated, most of the synthesized compounds did not show antiproliferative activity, thus reflecting the strict structural requirements for eliciting antiproliferative activity, which is in good agreement with many previous studies on colchicine-site ligands showing that structural variations resulted in large potency changes [22,24,28,50]. Frentizole, although not the most potent antiproliferative agent of the series, has the broader antiproliferative profile and is, therefore, the optimal candidate for repurposing.

Most of the compounds that showed antiproliferative effects in the library screening assays did so by acting on tubulin. To confirm that the observed antiproliferative effects were related to tubulin effects, we studied the effects of the active compounds on the microtubule network of the HeLa cells. The HeLa cells were sensitive to all of the active compounds, while the other cell lines, such as the glioblastoma cell line U87MG, were only sensitive to frentizole and, therefore, would render a comparison unfeasible. The compounds caused a net decrease in the microtubule networks of the HeLa cells, as expected for tubulin inhibitors such as the colchicine-site ligands. Further, we studied the effect of the compounds on the cell cycle populations over time, as colchicine-site ligands typically induce a G_2_/M arrest followed by an apoptotic response that occurs at the expense of the G_2_/M population. Consistent with other colchicine-site tubulin inhibitors, frentizole and its active analogs caused a progressive arrest at the G_2_/M populations of the cell cycle, followed by a progressive increase in the subG_0_/G_1_ populations that are considered apoptotic cells. We characterized the death associated with the treatments with the dual-staining flow cytometry studies as early apoptotic death. All of these results are consistent with a tubulin inhibitor. We studied the potential binding modes of the active compounds to the colchicine site of tubulin with molecular docking approaches. The active compounds might bind at the AB or AC sites depending on the configuration considered and the substituents present on the BZT ring.

All of these results indicate that frentizole acts as an antimitotic agent and set the ground for its repurposing for cancer chemotherapy. The low toxicity of frentizole is a favorable property as toxicity is one of the main drawbacks of tubulin inhibitors for the treatment of cancer. Furthermore, the activity of frentizole against the glioblastoma cell line U87MG with a low micromolar IC_50_ value (7.33 μM) compares very favorably with the only available therapy against glioblastoma, temozolomide, which has in vitro IC_50_ values against this cell line that are in the millimolar range [50]. Recently, glioblastoma tumor cells have been shown to be especially sensitive to microtubule-targeting agents, and, therefore, the activity here described for frentizole suggests it as a promising option for drug repurposing against glioblastomas [50,64]. Furthermore, frentizole is able to cross the blood–brain barrier (BBB), thus making it a promising option for the treatment of this devastating disease [65].

## 4. Materials and Methods

### 4.1. Chemistry

#### 4.1.1. General Chemical Techniques

The purchased reagents were used without further purification. Solvents (dichloromethane, methanol, acetonitrile, and ethyl acetate) were dried and stored over molecular sieves. Precoated silica gel polyester plates (0.25 mm thickness) with a UV fluorescence indicator 254 (Polychrom SI F254) were used for the analytical TLC. Chromatographic separations were performed on silica gel columns with flash (Kieselgel 40, 0.040–0.063; Merck, Madrid, Spain) chromatography. The ^1^H NMR and ^13^C NMR spectra were recorded on a Varian Mercury 400/100 MHz spectrometer or a Bruker SY spectrometer at 400/100 MHz, with the samples dissolved in CDCl_3_, CD_3_OD, or DMSO-d_6_. Chemical shifts (δ) are given in ppm and coupling constants (J values) are in Hz. The IR spectra in the KBr disks were run on a Nicolet Impact 410 Spectrophotometer. The HRMS analyses were conducted using a hybrid QSTAR XL quadrupole/time of flight spectrometer.

#### 4.1.2. General Synthetic Methods

##### General Synthetic Method A1 for Bromination via Bromine

Bromine was carefully added to a solution of the corresponding compound in AcOH. After stirring the reaction mixture at room temperature for 24 h, it was then poured onto ice, extracted with EtOAc, and washed with 5% NaHCO_3_. The organic layer was dried over anhydrous Na_2_SO_4_, filtered, and rotatory evaporated.

##### General Synthetic Method A2 for Bromination via *N*-Bromosuccinimide (NBS)

NBS was added to a solution of the corresponding compound in CH_2_Cl_2_ and stirred at room temperature for 24 h. Then, it was poured onto ice and washed with 1N HCl and NaHCO_3_. The organic layer was dried over anhydrous Na_2_SO_4_, filtered, and evaporated under vacuum.

##### General Synthetic Method B for Amide Bond Formation

An excess of SOCl_2_ was added to the corresponding carboxylic acid and stirred at 70 °C for 24 h. After evaporation of the SOCl_2_, the acyl chloride obtained was added to a solution of the corresponding amine and triethylamine (0.5 mL) in CH_2_Cl_2_ and stirred at room temperature for 24 h to 5 days. It was then poured onto cold water and washed with 1 N HCl, 5% NaHCO_3_, and brine. The organic layer was dried, filtered, and rotatory evaporated.

##### General Synthetic Method C for Urea Bond Formation

The corresponding cyanate or isocyanate was added to a solute ion of the amine and triethylamine in CH_2_Cl_2_ and stirred at room temperature for 24 h to 1 week under N_2_ atmosphere. The reaction mixture was then poured onto cold water and washed with brine, and the organic layer dried over anhydrous Na_2_SO_4_, filtered, and evaporated under vacuum.

##### General Synthetic Method D for Carbamate Formation

A mixture of the corresponding chloroformate, amine, and triethylamine in CH_2_Cl_2_ was stirred at room temperature for 5 to 24 h under an N_2_ atmosphere. The reaction mixture was then poured onto cold water, and the organic layer was dried, filtered, and evaporated.

##### General Synthetic Method E for Methylation

An excess of powdered KOH and methyl iodide was added to a solution of the corresponding compound in acetonitrile. It was stirred for 24 h at room temperature and, if required, more methyl iodide was added. The reaction mixture was poured onto ice washed with 1N HCl, 5% NaHCO_3_, and brine, and the organic layer was dried over anhydrous Na_2_SO_4_, filtered, and evaporated under vacuum.

#### 4.1.3. Chemical Synthesis and Characterization (Figure S8)

##### 4-Bromo-6-methylbenzo[d]thiazol-2-amine (**1b**)

Following general procedure A2, 6-methylbenzo[*d*]thiazol-2-amine (3.00 g, 18.3 mmol) was brominated using bromine (1.22 mL, 23.8 mmol). Compound **1** was isolated by column chromatography (EtOAc/Hex 1:1). Yield: 43% (1.92 g).

Mp (EtOAc): 211–212 °C. ^1^H-NMR (400 MHz, CDCl_3_): δ 2.37 (3H, s, CH_3_); 5.82 (2H, s); 7.32 (2H, s). ^13^C-NMR (100 MHz, DMSOD_6_): δ 20.7 (CH_3_); 110.3 (C); 120.9 (CH); 129.7 (CH); 131.9 (C); 132.0 (C); 149.0 (C); 166.7 (C). GC-MS (C_8_H_7_BrN_2_S^+^): 242 (M^+^).

##### *N*-(6-methylbenzo[*d*]thiazol-2-yl)benzamide (**3a**)

Following general procedure B, benzoyl chloride (275 μL, 2.37 mmol) and 6-methylbenzo[*d*]thiazol-2-amine (259 mg, 1.58 mmol) yielded amide **3a**. Yield (crystals): 74% (312 mg).

M.p. (MeOH/CH_2_Cl_2_): 222–224 °C. IR (KBr): 3058, 1676, 1604, 1551, 1467, 1300 cm^−1^. ^1^H-NMR (400 MHz, CDCl_3_): δ 2.48 (3H, s, CH_3_); 7.20 (1H, d, *J* = 8.0 Hz); 7.43 (1H, t, *J* = 8.0 Hz); 7.50 (2H, t, *J* = 8.0 Hz); 7.59–7.65 (2H, m); 8.03 (2H, d, *J* = 8.0 Hz). ^13^C-NMR (100 MHz, DMSO-D_6_): δ 21.4 (CH_3_); 120.4 (CH); 121.7 (CH); 127.9 (CH); 128.7 (2) (CH); 129.1 (2) (CH); 132.1 (C); 132.4 (C); 133.2 (C); 133.6 (CH); 146.7 (C); 158.5 (C); 166.3 (C). HRMS (C_15_H_12_N_2_NaO_2_S^+^): calculated 291.0563 (M+Na^+^), found 291.0568.

##### *N*-(5,6-dimethylbenzo[*d*]thiazol-2-yl)benzamide (**3c**)

Following general method B, benzoyl chloride (230 mg, 1.98 mmol) and 5,6-dimethybenzo[*d*]thiazol-2-amine (251 mg, 1.41 mmol) yielded compound **3c**. Yield: 51% (204 mg).

M.p. (MeOH): 210 °C. RMN ^1^H (CDCl_3_): 2.38 (6H, s, CH_3_); 7.56 (3H, m); 7.65 (2H, m); 8.04 (2H, d, *J* = 8.0 Hz). HRMS (C_15_H_12_ N_2_O_2_S^+^): calculated 283.0899 (M+H^+^), found 283.0896. ^13^C-RMN was not obtained because of insolubility problems.

##### *N*-(5,6-dimethylbenzo[*d*]thiazol-2-yl)-N-methylbenzamide (**3ca**)

Following general method E, compound **3c** (122 mg, 0.43 mmol) was methylated. Methyl iodide (81 μL, 1.31 mmol). Yield (crystals): 25% (33 mg).

M.p. (MeOH/CH_2_Cl_2_): 231 °C. ^1^H-RMN (CDCl_3_): δ 2.34 (3H, s, CH_3_); 2.39 (3H, s, CH_3_); 3.96 (3H, s, NCH_3_); 7.14 (1H, s); 7.48 (4H, m); 8.38 (2H, d, *J* = 7.2 Hz). ^13^C-RMN (100 MHz, CDCl_3_): δ 19.1 (CH_3_); 19.9 (CH_3_); 31.7 (CH_3_); 111.6 (CH); 122.6 (CH); 123.4 (C); 127.0 (2) (CH); 128.6 (2) (CH); 131.2 (CH); 132.5 (C); 135.1 (C); 135.6 (C); 136.2 (C); 167.2 (C); 174.2 (C). HRMS (C_17_H_17_N_2_OS^+^): calculated 297.1056 (M+H^+^), found 297.1057.

##### *N*-(6-methoxybenzo[*d*]thiazol-2-yl)benzamide (**3e**)

Following general procedure B, benzoyl chloride (230 μL, 1.98 mmol) and 6-methoxybenzo[*d*]thiazol-2-amine (254 mg, 1.41 mmol) yielded amide **3e**. Yield (crystals): 48% (190 mg).

M.p. (CH_2_Cl_2_): 195 °C. ^1^H-RMN (CDCl_3_): δ 3.86 (3H, s); 6.92 (1H, dd, *J* = 2.4 and 8.6 Hz); 7.31 (1H, d, *J* = 2.4 Hz); 7.32 (1H, d, *J* = 8.6 Hz); 7.47 (2H, t, *J* = 7.6 Hz); 7.58 (1H, t, *J* = 7.6 Hz); 7.96 (2H, d, *J* = 7.6 Hz). ^13^C-RMN (100 MHz, CDCl_3_): δ 55.7 (CH_3_); 103.9 (CH); 115.0 (CH); 121.2 (CH); 128.1 (2) (CH); 128.9 (2) (CH); 132.2 (C); 132.9 (CH); 141.7 (C); 156.7 (C); 158.3 (C); 166.3 (C). HRMS (C_15_H_12_ N_2_O_2_S^+^): calculated 285.0692 (M+H^+^), found 285.0704.

##### *N*-(6-methoxybenzo[*d*]thiazol-2-yl)-N-methylbenzamide (**3ea**)

Following general method E, compound **3e** (160 mg, 0.56 mmol) was alkylated using methyl iodide (110 μL, 1.8 mmol) yielding compound **3ea**. Yield (crystals): 8% (14 mg).

^1^H-RMN (CDCl_3_): δ 3.87 (3H, s); 3.97 (3H, s); 7.05 (1H, d, *J* = 8.4 Hz); 7.22 (1H, s); 7.27 (1H, d, *J* = 8.4 Hz); 7.48 (3H, m); 8.38 (2H, dd, *J* = 8.0 Hz). ^13^C-RMN (100 MHz, CDCl_3_): δ 32.3 (CH_3_); 55.9 (CH_3_); 106.6 (CH); 111.9 (CH); 114.7 (CH); 127.9 (C); 128.1 (2) (CH); 129.4 (2) (CH); 131.1 (C); 131.8 (CH); 136.6 (C); 156.7 (C); 167.1 (C); 174.6 (C). HRMS (C_16_H_15_ N_2_O_2_S^+^): calculated 299.0849 (M+H^+^), found 299.0857.

##### *N*-(6-methylbenzo[*d*]thiazol-2-yl)-2-phenylacetamide (**4a**)

Following general method B, 2-phenylacetyl chloride (285 μL, 2.16 mmol) and 6-methylbenzo[d]thiazol-2-amine (236 mg, 1.44 mmol) yielded amide **4a**. Yield (crystals): 72% (292 mg).

M.p. (MeOH/CH_2_Cl_2_): 190–191 °C. IR (KBr): 3127, 1697, 1534, 1454, 1263 cm^−1^. ^1^H-NMR (400 MHz, CDCl_3_): δ 2.46 (3H, s, CH_3_); 3.87 (2H, s, CH_2_); 7.24 (1H, m,); 7.30–7.41 (5H, m); 7.59 (2H, m). ^13^C-NMR (100 MHz, CDCl_3_): δ 21.5 (CH_3_); 43.4 (CH_2_); 120.1 (CH); 121.3 (CH); 127.9 (CH); 128.0 (CH); 129.2 (2) (CH); 129.4 (2) (CH); 132.1 (C); 132.8 (C); 134.2 (C); 145.6 (C); 158.0 (C); 169.4 (C). HRMS (C_16_H_14_N_2_NaOS+): calculated 305.0719 (M+Na^+^), found 305.0719.

##### 1-(4-Bromo-6-methylbenzo[*d*]thiazol-2-yl)-3-phenylurea (**4b**)

Following general method B, 2-phenylacetyl chloride (119 μL, 0.90 mmol) and compound **1b** (146 mg, 0.60 mmol) yielded amide **4b**. Yield (crystals): 57% (122 mg).

M.p. (MeOH/CH_2_Cl_2_): 234–236 °C. IR (KBr): 3142, 1651, 1599, 1328, 1266 cm^−1^. ^1^H-NMR (400 MHz, CDCl_3_): δ 2.43 (3H, s, CH_3_); 3.85 (2H, s, CH_2_); 7.26 (2H, m); 7.36 (3H, m); 7.44 (1H, s); 7.53 (1H, s); 9.08 (1H, s). ^13^C-NMR (100 MHz, CDCl_3_): δ 21.1 (CH_3_); 43.8 (CH_2_); 113.7 (C); 120.5 (CH); 128.0 (CH); 129.2 (2) (CH); 129.4 (2) (CH); 130.9 (CH); 32.5 (C); 133.0 (C); 135.4 (C); 144.5 (C); 157.8 (C); 169.7 (C). HRMS (C_16_H_14_BrN_2_OS^+^): calculated 361.0005 (M+H^+^), found 361.0000.

##### 1-Ethyl-3-(6-methylbenzo[*d*]thiazol-2-yl)urea (**5a**)

Following general method C, ethyl isocyanate (208 μL, 2.63 mmol) and 6-methylbenzo[d]thiazol-2-amine (288 mg, 1.75 mmol) yielded urea derivative **5a**. Yield (crystals): 50% (205 mg).

M.p. (CH_2_Cl_2_): 312–313 °C. IR (KBr): 3325, 1709, 1549, 1468, 1275 cm^−1^. ^1^H-NMR (400 MHz, CDCl_3_): δ 1.26 (3H, t, *J* = 7.2 Hz); 2.44 (3H, s); 3.42 (2H, q, *J* = 7.2 Hz); 7.20 (1H, dd, *J* = 1.2 and 8.4 Hz); 7.52 (1H, s); 7.61 (1H, d, *J* = 8.4 Hz). ^13^C-NMR (100 MHz, DMSO-D_6_): δ 15.6 (CH_3_); 21.3 (CH_3_); 34.7 (CH_2_); 119.6 (CH); 121.4 (CH); 127.4 (CH); 131.9 (C); 132.3 (C); 141.4 (C); 154.2 (C); 156.5 (C). HRMS (C_11_H_13_N_3_NaOS^+^): calculated 258.0672 (M+Na^+^), found 258.0671.

##### 1-(4-Bromo-6-methylbenzo[*d*]thiazol-2-yl)-3-ethylurea (**5b**)

Following general method C, ethyl isocyanate (90 μL, 1.14 mmol) and bromo derivative 1 (181 mg, 0.74 mmol) yielded compound **5b**. Yield (crystals): 63% (147 mg).

M.p. (MeOH): 279–280 °C. IR (KBr): 3367, 3111, 1704, 1682, 1563, 1442, 1259 cm^−1^. ^1^H-NMR (400 MHz, DMSO-D_6_): δ 1.05 (3H, t, *J* = 6.8 Hz); 2.33 (3H, s); 3.16 (2H, q, *J* = 6.8 Hz); 6.52 (1H, s); 7.38 (1H, s); 7.64 (1H, s); 11.16 (1H, s). ^13^C-NMR (100 MHz, DMSO-D_6_): δ 15.6 (CH_3_); 20.9 (CH_3_); 34.8 (CH_2_); 112.6 (C); 121.2 (CH); 130.2 (CH); 132.9 (C); 134.1 (C); 145.7 (C); 154.0 (C); 160.2 (C). HRMS (C_11_H_12_BrN_3_NaOS^+^): calculated 335.9777 (M+Na^+^), found 335.9791.

##### 1-(5,6-Dimethylbenzo[*d*]thiazol-2-yl)-3-ethylurea (**5c**)

Following general procedure C, ethyl isocyanate (127 μL, 1.60 mmol) and 6-methoxybenzo[d]thiazol-2-amine (252 mg, 1.40 mmol) yielded compound **5c**. Yield (crystals): 84% (294 mg).

^1^H-RMN (CDCl_3_): δ 1.26 (3H, m); 2.34 (3H, s); 2.35 (3H, s); 3.42 (2H, m); 7.46 (1H, s); 7.52 (1H, s). HRMS (C_12_H_16_N_3_OS^+^): calculated 250.1014 (M+H^+^), found 250.1009. ^13^C-RMN was not obtained because of insolubility.

##### 1-(4-Bromo-5,6-dimethylbenzo[*d*]thiazol-2-yl)-3-ethylurea (**5d**)

Following general procedure A2, compound **5c** (125 mg, 0.50 mmol) was brominated. NBS (99 mg, 0.56 mmol). Yield (crystals): 46% (76 mg).

^1^H-RMN (CDCl_3_): δ 1.28 (3H, m); 2.78 (6H, s); 3.42 (2H, m); 7.42 (2H, s). HRMS (C_12_H_15_N_3_OBrS^+^): calculated 328.0119 (M+H^+^), found 328.0114. ^13^C-RMN was not obtained because of insolubility.

##### 1-Ethyl-3-(6-methoxybenzo[*d*]thiazol-2-yl)urea (**5e**)

Following general method C, ethyl isocyanate (130 μL, 1.64 mmol) and 6-methoxybenzo[d]thiazol-2-amine (252 mg, 1.40 mmol) yielded compound **5e**. Yield (crystals): 50% (178 mg).

^1^H-RMN (CDCl_3_): δ 1.23 (3H, m); 3.49 (2H, m); 3.82 (3H, s); 6.97 (1H, dd, *J =* 2.4 and 8.5 Hz); 7.20 (1H, d, *J =* 2.4 Hz); 7.62 (1H, d, *J =* 8.5 Hz). ^13^C-RMN (100 MHz, CD_3_OD): δ 14.9 (CH_3_); 35.0 (CH_2_); 55.7 (CH_3_); 104.5 (CH); 114.4 (CH); 120.2 (CH); 132.2 (C); 143.1 (C); 154.6 (C); 156.2 (C); 159.8 (C). HRMS (C_15_H_13_N_3_NaO_2_S+): calculated 274.0626 (M+Na^+^), found 274.0621.

##### 1-(4-Bromo-6-methoxybenzo[*d*]thiazol-2-yl)-3-ethylurea (**5f**)

Following general method A2, urea derivative **5e** (122 mg, 0.49 mmol) was brominated. NBS (97 mg, 0.53 mmol). Yield (crystals): 14% (24 mg).

^1^H-RMN (CDCl_3_): δ 1.22 (3H, m); 3.34 (2H, m); 3.78 (3H, s); 7.03 (1H, s); 7.04 (1H, s); 10.39 (1H, s). ^13^C-RMN (100 MHz, CDCl_3_): δ 14.8 (CH_3_); 35.1 (CH_2_); 55.9 (CH_3_); 104.1 (CH); 113.0 (C); 117.3 (CH); 132.5 (C); 141.6 (C); 154.3 (C); 156.2 (C); 159.9 (C). HRMS (C_11_H_13_N_3_O_2_BrS+): calculated 329.9912 (M+Na^+^), found 329.9906.

##### 1-(6-Methylbenzo[*d*]thiazol-2-yl)-3-phenylurea (**6a**)

Following general procedure C, phenyl isocyanate (258 μL, 2.37 mmol) and 6-methylbenzo[d]thiazol-2-amine (260 mg, 1.58 mmol) yielded urea derivative **6a**. Yield (crystals): 81% (365 mg).

M.p. (CH_2_Cl_2_): 327–329 °C. IR (KBr): 3206, 1726, 1692, 1542, 1291 cm^−1^. ^1^H-NMR (400 MHz, CDCl_3_): δ 2.45 (3H, s); 7.13 (1H, t, *J* = 7.6 Hz); 7.23 (1H, dd, *J* = 1.2 and 8.0 Hz); 7.34 (2H, t, *J* = 7.6 Hz); 7.52 (2H, t, *J* = 7.6 Hz); 7.54 (1H, s); 7.62 (1H, d, *J* = 8.0 Hz). ^13^C-NMR (100 MHz, DMSO-D_6_): δ 21.3 (CH_3_); 119.2 (2) (CH); 119.4 (CH); 121.6 (CH); 123.4 (CH); 127.6 (CH); 129.4 (2) (CH); 131.7 (C); 132.7 (C); 139.0 (C); 146.3 (C); 152.6 (C); 159.4 (C). HRMS (C_15_H_13_N_3_NaOS+): calculated 306.0672 (M+Na^+^), found 306.0676.

##### 1-(5,6-Dimethylbenzo[*d*]thiazol-2-yl)-3-phenylurea (**6c**)

Following general method C, phenyl isocyanate (370 μL, 3.40 mmol) and 5,6-dimethylbenzo[d]thiazol-2-amine (500 mg, 2.81 mmol) yielded compound **6c**. Yields (solid): 90% (754 mg).

M.p. (CH_2_Cl_2_): >290 °C. ^1^H-RMN (CDCl_3_): δ 2.33 (6H, s); 7.12 (1H, bt); 7.34 (2H, bt); 7.49 (2H, s); 7.53 (2H, da). ^13^C-NMR (100 MHz, DMSO-D_6_): δ 19.9 (CH_3_); 20.1 (CH_3_); 119.2 (2) (CH); 121.9 (CH); 123.3 (CH); 129.4 (2) (CH); 132.0 (C); 134.9 (C); 139.0 (C). HRMS (C_16_H_16_N_3_OS+): calculated 298.1014 (M+H^+^), found 298.1008.

##### 1-(5,6-Dimethylbenzo[*d*]thiazol-2-yl)-1-methyl-3-phenylurea (**6ca**)

Following general procedure F, compound **6c** (100 mg, 0.34 mmol) was methylated. Methyl iodide (53 μL, 0.84 mmol). Yield (crystals): 63% (66 mg).

M.p. (MeOH/CH_2_Cl_2_): 179 °C. ^1^H-RMN (CDCl_3_): δ 2.32 (3H, s); 2.36 (3H, s); 3.72 (3H, s); 7.01 (1H, s); 7.03 (1H, t, *J* = 8.4 Hz); 7.31 (2H, t, *J* = 8.4 Hz); 7.36 (1H, s); 7.58 (2H, d, *J* = 8.4).^13^C-NMR (100 MHz, DMSO-D_6_): 19.6 (CH_3_); 20.2 (CH_3_); 32.15 (CH_3_); 112.7 (CH); 118.7 (2) (CH); 121.6 (C); 122.2 (CH); 122.9 (C); 123.0 (CH); 129.0 (2) (CH); 132.0 (C); 135.7 (C); 136.2 (C); 140.9 (C); 161.6 (C).HRMS (C_17_H_18_N_3_OS^+^): calculated 312.1171 (M+H^+^), found 312.1165.

##### 1-(6-Methoxybenzo[*d*]thiazol-2-yl)-3-phenylurea (**6e**)

Following general procedure C, phenyl isocyanate (362 μL, 3.33 mmol) and 6-methoxybenzo[*d*]thiazol-2-amine (500 mg, 2.77 mmol) yielded compound **6e**. Yield (solid): 83% (695 mg).

M.p. (CH_2_Cl_2_): 280 °C. ^1^H-RMN (CDCl_3_): δ 3.86 (3H, s); 7.01 (1H, d, *J* = 8.4 Hz); 7.14 (1H, t, *J* = 7.6 Hz); 7.23 (1H, s); 7.35 (2H, t, *J* = 7.6 Hz); 7.52 (2H, d, *J* = 7.6 Hz); 7.68 (1H, d, *J* = 8.4 Hz). ^13^C-NMR (100 MHz, DMSO-D_6_): δ 56.0 (CH_3_); 105.4 (CH); 110.0 (C); 114.8 (CH); 119.2 (2) (CH); 123.4 (CH); 129.4 (2) (CH); 138.9 (C); 156.2 (C). HRMS (C_15_H_14_N_3_O_2_S+): calculated 300.0807 (M+H^+^), found 300.0801.

##### 1-(6-Methoxybenzo[*d*]thiazol-2-yl)-1-methyl-3-phenylurea (**6ea**)

Following general procedure F, compound **6e** (100 mg, 0.33 mmol) was methylated. Methyl iodide (52 μL, 0.83 mmol). Yield (crystals): 9% (8 mg).

M.p. (Hex/CH_2_Cl_2_): 189 °C. ^1^H-RMN (CDCl_3_): δ 3.72 (3H, s); 3.85 (3H, s); 6.96 (1H, dd, *J* = 2.4 and 8.6 Hz); 7.05 (1H, t, *J* = 8.0 Hz); 7.13 (1H, d, *J* = 2.4 Hz); 7.13 (1H, d, *J* = 8.6 Hz); 7.32 (2H, t, *J* = 8.0 Hz); 7.58 (2H, d, *J* = 8.0 Hz). ^13^C-NMR (100 MHz, DMSO-D_6_): δ 32.3 (CH_3_); 56.2 (CH_3_); 107.5 (CH); 112.6 (CH); 114.2 (CH); 118.7 (2) (CH); 122.3 (CH); 127.2 (C); 129.0 (2) (CH); 131.8 (C); 140.8 (C); 156.3 (C); 160.5 (C); 165.0 (C). HRMS (C_16_H_16_N_3_O_2_S+): calculated 314.0963 (M+H^+^), found 314.0958.

##### 1-Benzyl-3-(6-methylbenzo[*d*]thiazol-2-yl)urea (**7a**)

Following general method C, benzyl isocyanate (314 μL, 2.54 mmol) and 6-methylbenzo[*d*]thiazol-2-amine (278 mg, 1.69 mmol) yielded compound **7a**. Yield (crystals): 92% (275 mg).

M.p. (CH_2_Cl_2_): 283.4–285.7 °C. IR (KBr): 3344, 1708, 1537, 1275 cm^−1^. ^1^H-NMR (400 MHz, CDCl_3_): δ 2.44 (3H, s); 4.54 (2H, d, *J* = 5.6 Hz); 7.17 (1H, dd, *J* = 1.2 and 8.0 Hz); 7.26–7.33 (5H, m); 7.51 (1H, s); 7.57 (1H, d, *J* = 8.0 Hz). ^13^C-NMR (100 MHz, DMSO-D_6_): δ 21.3 (CH_3_); 43.4 (CH_2_); 119.7 (CH); 121.5 (CH); 127.4 (CH); 127.5 (CH); 127.6 (2) (CH); 128.9 (2) (CH); 131.9 (C); 132.5 (C); 139.8 (C); 147.4 (C); 154.4 (C); 159.4 (C). HRMS (C_16_H_15_N_3_NaOS^+^): calculated 320.0828 (M+Na^+^), found 320.0834.

##### 1-Benzyl-3-(4-bromo-6-methylbenzo[*d*]thiazol-2-yl)urea (**7b**)

Following general method C, benzyl isocyanate (100 μL, 0.81 mmol) and compound 1 (125 mg, 0.51 mmol) yielded urea derivative **7b**. Yield (crystals): 65% (124 mg).

M.p. (MeOH): 204 °C. ^1^H-NMR (400 MHz, DMSO-D_6_): δ 2.34 (3H, s); 4.35 (2H, d, *J* = 6.0 Hz); 7.04 (1H, s); 7.24–7.35 (5H, m); 7.41 (1H, s); 7.67 (1H, s); 11.29 (1H, s). ^13^C-NMR (100 MHz, DMSO-D_6_): δ 20.9 (CH_3_); 43.4 (CH_2_); 112.6 (C); 121.2 (CH); 127.4 (CH); 127.6 (2) CH); 128.9 (2) (CH); 130.3 (CH); 132.9 (C); 134.2 (C); 139.8 (C); 145.7 (C); 154.2 (C); 160.2 (C). HRMS (C_16_H_15_BrN_3_OS^+^): calculated 376.0114 (M+H^+^), found 376.0122.

##### Ethyl (6-methylbenzo[*d*]thiazol-2-yl)carbamate (**8a**)

Following general method D, ethyl chloroformate (226 μL, 2.36 mmol) and 6-methylbenzo[*d*]thiazol-2-amine (259 mg, 1.58 mmol) yielded carbamate **8a**. Yield (crystals): 81% (300 mg).

M.p. (CH_2_Cl_2_): 252.8–254.0 °C. ^1^H-NMR (400 MHz, CDCl_3_): δ 1.41 (3H, t, *J* = 7.2 Hz); 2.45 (3H, s); 4.39 (2H, q, *J* = 7.2 Hz); 7.22 (1H, dd, *J* = 1.6 and 8.0 Hz); 7.59 (1H, s); 7.80 (1H, d, *J* = 8.0 Hz). ^13^C-NMR (100 MHz, DMSO-D_6_): δ 14.7 (CH_3_); 21.4 (CH_3_); 62.3 (CH_2_); 120.3 (CH); 121.6 (CH); 127.7 (CH); 132.0 (C); 133.1 (C); 147.6 (C); 154.4 (C); 159.2 (C). HRMS (C_11_H_12_N_2_NaO_2_S^+^): calculated 259.0512 (M+Na^+^), found 259.0517.

##### Ethyl (4-bromo-6-methylbenzo[*d*]thiazol-2-yl)carbamate (**8b**)

Following general method D ethyl chloroformate (60 μL, 0.63 mmol) and compound 1 (101 mg, 0.42 mmol) yielded carbamate **8b**. Yield (crystals): 79% (103 mg).

M.p. (MeOH): 177–178 °C. ^1^H-NMR (400 MHz, CDCl_3_): δ 1.37 (3H, t, *J* = 7.2 Hz); 2.44 (3H, s); 4.33 (2H, q, *J* = 7.2 Hz); 7.44 (1H, s); 7.51 (1H, s); 8.50 (1H, s). ^13^C-NMR (100 MHz, DMSO-D_6_): δ 14.3 (CH_3_); 21.2 (CH_3_); 62.9 (CH_2_); 113.6 (C); 120.3 (CH); 130.7 (CH); 132.8 (C); 134.9 (C); 145.3 (C); 153.2 (C); 159.4 (C). HRMS (C_11_H_12_BrN_2_O_2_S^+^): calculated 314.9797 (M+H^+^), found 314.9790.

##### Phenyl (6-methylbenzo[*d*]thiazol-2-yl)carbamate (**9a**)

Following general method D, phenyl chloroformate (345 μL, 2.75 mmol) and 6-methylbenzo[*d*]thiazol-2-amine (301 mg, 1.83 mmol) yielded compound **9a**. Yield (crystals): 58% (304 mg).

M.p. (MeOH): 251–253 °C. IR (KBr): 3074, 1739, 1613, 1580, 1277, 1188 cm^−1^. ^1^H-NMR (400 MHz, CDCl_3_): δ 2.46 (3H, s); 7.22 (1H, bd, *J* = 8.4 Hz); 7.26–7.33 (3H, m); 7.44 (2H, t, *J* = 8.0 Hz); 7.61 (1H, s); 7.80 (1H, d, *J* = 8.4 Hz). ^13^C-NMR (100 MHz, DMSO-D_6_): δ 21.4 (CH_3_); 120.3 (CH); 121.8 (CH); 122.3 (2) (CH); 126.6 (CH); 127.9 (CH); 130.1 (2) (CH); 132.0 (C); 133.4 (C); 147.2 (C); 150.5 (C); 153.3 (C); 159.3 (C). HRMS (C_15_H_12_N_2_NaO_2_S^+^): calculated 307.0512 (M+Na^+^), found 307.0519.

##### Phenyl (4-bromo-6-methyl-N-phenoxycarbonylbenzo[*d*]thiazol-2-yl)carbamate (**9b**)

Following general method D, benzothiazole derivative **1** (136 mg, 0.56 mmol) reacted with phenyl chloroformate (110 μL, 0.88 mmol) to yield compound **9b**. Yield (crystals): 22% (47 mg).

M.p. (MeOH): 100 °C. ^1^H-NMR (400 MHz, CDCl_3_): δ 2.48 (3H, s); 7.29–7.33 (6H, m); 7.43 (4H, t, *J* = 8.8 Hz); 7.54 (1H, s); 7.59 (1H, s). ^13^C-NMR (100 MHz, CDCl_3_): δ 21.3 (CH_3_); 116.0 (C); 120.5 (CH); 121.1 (4) (CH); 126.9 (2) (CH); 129.6 (4) (CH); 131.4 (CH); 135.3 (C); 137.0 (C); 145.8 (C); 149.3 (C); 150.3 (C); 156.4 (C). HRMS (C_22_H_15_BrN_2_NaO_4_S^+^): calculated 504.9828 (M+Na^+^), found 504.9824.

##### Benzyl (6-methylbenzo[*d*]thiazol-2-yl)carbamate (**10a**)

Following general method D, benzyl chloroformate (217 μL, 2.21 mmol) and 6-methylbenzo[*d*]thiazol-2-amine (241 mg, 1.47 mmol) yielded carbamate **10a**. Yield (crystals): 13% (58 mg). M.p. (CH_2_Cl_2_): 254–255 °C. ^1^H-NMR (400 MHz, CDCl_3_): δ 2.43 (3H, s); 5.33 (2H, s); 6.97 (1H, d, *J* = 8.0 Hz); 7.38–7.44 (5H, m); 7.55 (1H, s); 7.59 (1H, d, *J* = 8.0 Hz). ^13^C-NMR (100 MHz, DMSO-D_6_): δ 21.4 (CH_3_); 67.6 (CH_2_); 120.3 (CH); 121.7 (CH); 127.8 (CH); 128.7 (CH); 128.8 (2) (CH); 129.0 (2) (CH); 132.0 (C); 133.1 (C); 136.2 (C); 147.6 (C); 154.3 (C); 159.1 (C). HRMS (C_16_H_15_N_2_O_2_S^+^): calculated 299.0849 (M+H^+^), found 299.0859.

### 4.2. Biology

#### 4.2.1. Cell Culture Conditions

The cell lines were from ATTC (Manassas, VA, USA). HeLa (human cervix epithelioid carcinoma), U87 MG (human glioblastoma), A172 (human glioblastoma), and HEK-293 (human embryonic kidney) were grown in Dulbecco’s modified Eagle’s medium (DMEM, Gibco, Waltham, MA, USA), supplemented with 10% (*v*/*v*) heat-inactivated fetal bovine serum (HIFBS, Sigma-Aldrich, St. Louis, MO, USA), 2 mM L-glutamine, 100 μg/mL streptomycin, and 100 units/mL penicillin (Gibco, Waltham, MA, USA) at 37 °C in humidified 95% air and 5% CO_2_. The cells were periodically tested for Mycoplasma infection using MycoAlert kit (Lonza, Norwest, Australia), and only mycoplasma-free cells were employed in the experiments.

#### 4.2.2. Cell Growth Inhibition Assay

The effect of the compounds on the proliferation of human tumor cell lines was determined as previously described [50] using the MTT reagent 3-[4,5-dimethylthiazol-2-yl]-2,5-diphenyltetrazolium bromide, MTT Thiazolyl Blue Tetrazolium Bromide, Sigma-Aldrich, St. Louis, MO, USA) dissolved in PBS at 5 mg/mL, according to the manufacturer’s instructions. The cells were incubated in 96-well plates (100 μL/well), in complete DMEM or RPMI 1640 medium (see above), at 37 °C and 5% CO_2_ atmosphere for 24 h to allow for cell attachment to the plate at the following concentrations: 30,000 cells/mL (U87MG, A172, J774, and HEK-293 cells) or 15,000 cells/mL (HeLa cells). Then, every compound was added (10 μL/well) to a final concentration of 10 μM. Untreated cells were used as negative controls. The antiproliferative activity of the compounds was measured 72 h after drug exposure using the MTT assay. The IC_50_ value (the drug concentration required to inhibit 50% of the cell growth in respect the untreated control) was determined for those compounds showing antiproliferative effects in the initial screening at 10 μM. For this purpose, the compounds were used at different concentrations ranging from 10^−10^ to 10^−2^ M. Measurements were performed in triplicate and each experiment was repeated three times. The IC_50_ values were determined using Origin software (OriginLab, Washington, DC, USA).

#### 4.2.3. Cell Cycle Analysis

HeLa cells (8 × 10^4^ cells/mL) were seeded in 6-well plates (2 mL/well) and incubated in complete DMEM medium at 37 °C and 5% CO_2_ atmosphere for 24 h. Then, the medium was replaced with fresh complete DMEM in the presence or absence of the selected compounds (**3ea**, **5b**, **5f**, and frentizole **6e**) at 2 μM. Untreated cells were used as negative controls. Cells were harvested 24, 48, or 72 h following treatment and fixed in ice-cold ethanol/PBS (7:3) overnight. The cells were then washed twice with PBS, suspended in PBS and incubated overnight in darkness with 0.2 mg/mL RNase A (Sigma-Aldrich, St. Louis, MO, USA), 50 μg/mL propidium iodide (Sigma-Aldrich, St. Louis, MO, USA), and Triton 10× at room temperature. A BD Accuri™ C6 Plus Flow Cytometer (BD Bioscience, Madrid, Spain) was used to analyze samples, and BD Accuri™ C6 Software (version 1.0.264.21) was used for data analysis.

#### 4.2.4. Apoptotic Cell Death Quantification

Annexin V-FITC/PI apoptosis detection kit (Immunostep, Salamanca, Spain) was used to quantify cell death of HeLa cells by following the manufacturer’s guidelines. An amount of 8 × 10^4^ cells/mL was seeded in 12-well plates (1 mL/well) and incubated in complete DMEM medium at 37 °C and 5% CO_2_ atmosphere for 24 h. Then, the medium was replaced with fresh complete DMEM in the presence or absence of the selected compounds (**3ea**, **5b**, **5f**, and frentizole **6e**) at 2 μM. Untreated cells were used as negative controls. After 72 h of incubation, cells were collected, centrifugated, resuspended in the Annexin V binding buffer, and stained with Annexin V-FITC/PI. Cells were then incubated in darkness for 15 min at room temperature. Samples were analyzed using BD Accuri™ C6 Plus Flow Cytometer (BD Biosciences), and acquired data were analyzed using BD Accuri™ C6 Software (version 1.0.264.21).

#### 4.2.5. Immunofluorescence Experiments

HeLa cells (8 × 10^4^ cells/mL) were seeded on 0.01% poly-L-lysine precoated square glass coverslips (22 mm^2^), deposited on 6-well plates (1 coverslip/well), and incubated in complete DMEM medium at 37 °C and 5% CO_2_ atmosphere. After 24 h the culture medium was replaced by fresh complete DMEM, and the cells were incubated in the presence of 2 μM or absence of the selected compounds (**3ea**, **5b**, **5f**, and frentizole **6e**) for 24 h. Untreated cells were used as negative controls. Then the medium was removed, and the coverslips were washed three times with PBS, fixed in 4% formaldehyde in PBS for 10 min, permeabilized with 0.5% Triton X-100 (Sigma-Aldrich, St. Louis, MO, USA) in PBS for 90 s at 4 °C, blocked with 10% BSA in PBS for 30 min and washed four times with PBS. Then, the coverslips were incubated for 1 h with anti-α-tubulin mouse monoclonal antibody (Sigma-Aldrich, St. Louis, MO, USA, 1:200 in PBS containing 3% BSA). After four washes with PBS, the coverslips were incubated with fluorescent secondary antibody Alexa Fluor 488 goat anti-mouse IgG (Molecular Probes, Invitrogen, Eugenen, OR, USA, 1:400 in PBS containing 1% BSA) for 1.5 h in darkness. After four washes with PBS, a droplet of ProLong™ Gold Antifade Mountant containing DAPI (ThermoFisher, Waltham, MA, USA) was added for cell nuclei staining. The samples were analyzed via confocal microscopy using a LEICA SP5 microscope DMI-6000V model coupled to a LEICA LAS AF 4.0 software computer.

### 4.3. Computational Studies

#### 4.3.1. Similarity Calculations

A collection of 7320 drugs with assigned International Nonproprietary Names (INN) was searched for similarity to the AC reference compounds using DataWarrior’s [31] default descriptor FragFp, a substructure fragment dictionary-based binary fingerprint that relies on a dictionary of 512 predefined structure fragments, where heteroatoms are often been replaced by wildcards, thus allowing for heteroatom replacements.

#### 4.3.2. Configurational Calculations

The configurations arising from the rotation of the amide and urea bonds were generated by conformational searches at the molecular mechanics level with the MMFF forcefield. Then, the obtained configurations were energy minimized by B3LYP DFT calculations at the 6–31 G* level with the Spartan 08 software package. The relative energies in vacuum and in water were calculated.

#### 4.3.3. Docking Calculations

The ensemble docking studies were conducted as previously described and accounting for the tubulin flexibility in the docking protocols by means of ensemble procedures. We achieved the sampling of the protein conformational space within the binding site using different tubulin structures from diverse colchicine-site ligands complexes [12,43]. 112 pdb X-ray crystal structures of the complexes of tubulin with different representative colchicine-site ligands without waters [55] in addition to 27 sites including water molecules that make intervening bridges between the ligands and the proteins added up to a total of 139 structures. Six additional structures from a previously described molecular dynamics simulation [56] on a tubulin–podophyllotoxin complex completed the set of 145 tubulin structures. We performed the docking studies using AutoDock 4.2 [58] with the Lamarckian genetic algorithm (LGA) 100−300 times for a maximum of 2.5 million energy evaluations, 150 individuals, and a maximum of 27,000 generations and in parallel with PLANTS [57] with the default settings and 10 runs per ligand. For the ligands with amide bonds and urea groups, we started the docking runs from all possible configurations and selected as the docking results for every ligand the best scored among all of them and for every individual configuration. We converted the scores of the different programs into Z-scores to allow for a comparison of the different scoring scales. We selected as the docking results the common poses for the two programs with the best consensus Z-scores. We applied in-house KNIME pipelines to automatically assign every pose to the colchicine subzones [44]. The RMSD between every pose and model scaffolds with no substituents and with colchicine binding-site ligands representative of binders occupying different subzones were calculated with LigRMSD [45]. Docked poses were analyzed with Chimera [46], Marvin [47], OpenEye [48], and JADOPPT [49].

#### 4.3.4. Molecular Dynamics Simulations and MMGBSA Re-Scoring

We re-scored the consensus docking poses using the AMBER scoring functionality included in DOCK 6.11 [66]. The consensus docked poses in complex with their respective targets were subjected to 100 steps of conjugate gradient minimization followed by 3 ns (3000 steps of 1 fs) of Langevin molecular dynamics simulations at 300 K in implicit solvent, followed by 100 steps of energy minimization. The all-atom AMBER forcefield were used for the proteins and the nucleotides and the general AMBER forcefield (GAFF) for the ligands. The ligands and the protein residues within 5 Å were allowed to freely move during the simulations. The total energy is represented by the solvation energy calculated using a Generalized Born solvation model (GB) and the electrostatic and van der Walls energy terms of the molecular mechanics (MM) interaction energy between the protein and the ligand [60].

## Figures and Tables

**Table 1:** Antiproliferative activity against the human tumor cell lines HeLa, A-172, and U87MG; the nontumorigenic cell line HEK-293; and the murine macrophage cell line J774. Potencies are expressed as IC_50_ values ^1^ in μM or as greater than the threshold concentration, as determined by the MTT assay *.

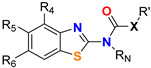											
Compound	R_4_	R_5_	R_6_	R_N_	X	R’	HeLa	A-172	U-87 MG	HEK-293	J-774
**3a**	H	H	Me	H	-	Ph	>10	>10	>10	>10	>10
**3c**	H	Me	Me	H	-	Ph	>10	>10	>10	5.68	7.73
**3ca**	H	Me	Me	Me	-	Ph	>10	>10	>10	>10	>10
**3e**	H	H	**O**Me	H	-	Ph	>10	>10	>10	>10	>10
**3ea**	H	H	**O**Me	Me	-	Ph	0.74	>10	>10	>10	>10
**4a**	H	H	Me	H	-	Bn	>10	>10	>10	>10	>10
**4b**	**Br**	H	Me	H	-	Bn	>10	>10	>10	>10	>10
**5a**	H	H	Me	H	**N**H	Et	>10	>10	>10	>10	>10
**5b**	**Br**	H	Me	H	**N**H	Et	0.49	>10	>10	>10	>10
**5c**	H	Me	Me	H	**N**H	Et	>10	>10	>10	>10	>10
**5d**	**Br**	Me	Me	H	**N**H	Et	1.24	>10	>10	>10	>10
**5e**	H	H	**O**Me	H	**N**H	Et	>10	>10	>10	>10	>10
**5f**	**Br**	H	**O**Me	H	**N**H	Et	0.7	>10	>10	>10	>10
**6a**	H	H	Me	H	**N**H	Ph	>10	>10	>10	>10	1.97
**6b**	**Br**	H	Me	H	**N**H	Ph	>10	>10	>10	>10	>10
**6c**	H	Me	Me	H	**N**H	Ph	>10	>10	>10	>10	0.27
**6ca**	H	Me	Me	Me	N	Ph	>10	>10	>10	>10	>10
**6e**	H	H	**O**Me	H	**N**H	Ph	1	>10	7.33	6.86	>10
**6ea**	H	H	**O**Me	Me	N	Ph	>10	>10	>10	>10	>10
**7a**	H	H	Me	H	**N**H	Bn	>10	>10	>10	>10	>10
**7b**	**Br**	H	Me	H	**N**H	Bn	>10	>10	>10	>10	>10
**8a**	H	H	Me	H	**O**	Et	>10	>10	>10	>10	0.26
**8b**	**Br**	H	Me	H	**O**	Et	>10	>10	>10	>10	>10
**9a**	H	H	Me	H	**O**	Ph	>10	>10	>10	>10	>10
**9b ^2^**	**Br**	H	Me	H	**O**	Ph	>10	>10	>10	>10	>10
**10a**	H	H	Me	H	**O**	Bn	>10	>10	>10	>10	+/−
**10b**	**Br**	H	Me	H	**O**	Bn	>10	>10	>10	>10	>10

^1^ IC_50_ is the drug concentration required to inhibit by 50% the growth of the corresponding cell line with respect to the untreated controls. Measurements were performed 72 h after drug treatments. ^2^ Compound **9b** has a second phenyl carbonate unit attached to the nitrogen. * Data are the mean of three independent experiments performed in triplicate. Unless indicated, standard deviations were below 1% of the mean values in all cases.

## Data Availability

The data presented in this study are available in the Supplementary Materials.

## Supplementary Materials

The following supporting information can be downloaded at: https://www.mdpi.com/article/10.3390/ijms242417474/s1.
