# Supplementary material for: Frentizole, a Nontoxic Immunosuppressive Drug, and Its Analogs Display Antitumor Activity via Tubulin Inhibition
Source: Int J Mol Sci. 2023 Dec 14;24(24):17474. doi: 10.3390/ijms242417474 (PMC10744269; doi:10.3390/ijms242417474)
Supplement: Supplementary file 1 [file ijms-24-17474-s001.zip › ijms-2721809-supplementary.pdf]

International Journal of
Molecular Sciences

MDPI

Article

Frentizole, a Nontoxic Immunosuppressive Drug, and Its Analogs Display Antitumor Activity via Tubulin Inhibition

Supplementary materials

Table S1. Chemical structures of the AC colchicine site ligands. The pdb IDs confirming their binding mode are indicated.

Name	Structure	Pdb ID	Ref
Nocodazole	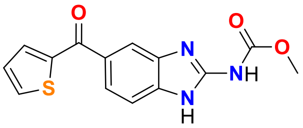	5CA1<br>7Z2P	[1, 2]
MI-181	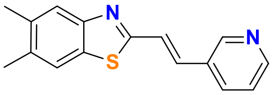	4YJ2	[3]
TN-16	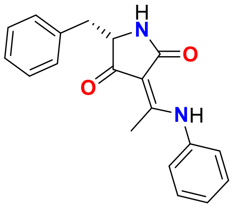	5C8Y	[4]
Plinabulin	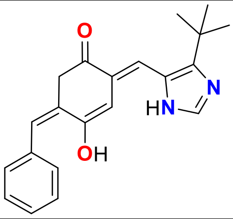	5C8Y<br>6S8K<br>6S8L	[2, 5]
TUB015	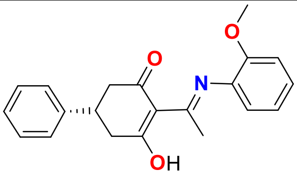	6FKL	[6]
TUB075	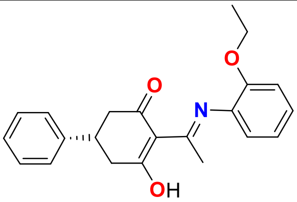	6FKJ	[6]
NSC 613863 (N2G)	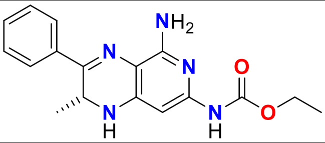	3N2G	[4]
N2K	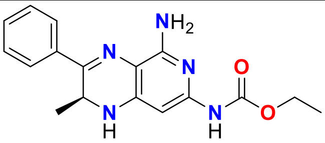	3N2K	[4]
Quinolin-6-yloxyacetamide	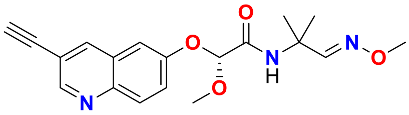	5O7A	[7]

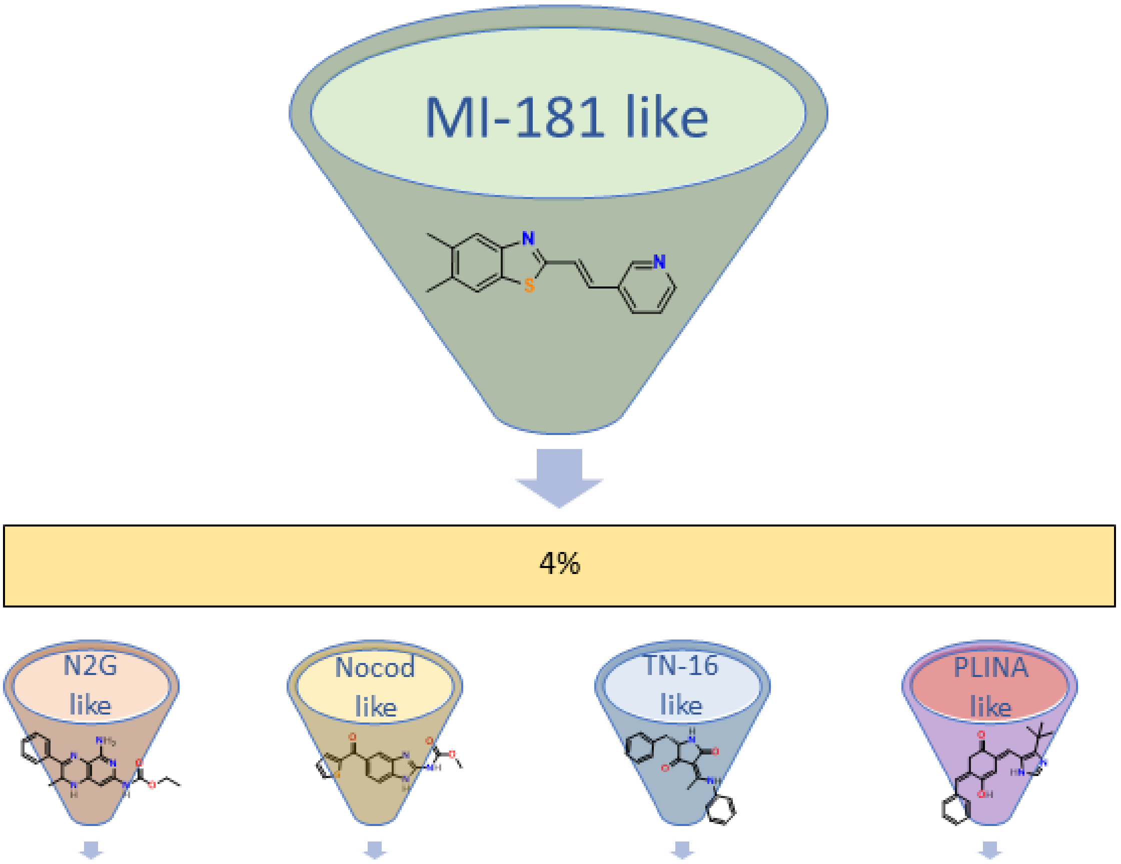

INN	INDICATION
Albendazole	Anthelminithic
Albendazole oxide	Anthelminithic
Cambendazole	Anthelminithic
Fenbendazole	Anthelminithic
Luxabendazole	Anthelminithic
Mebendazole	Anthelminithic
Oxfendazole	Anthelminithic
Parbendazole	Anthelminithic
flubendazole	Anthelminithic
Tenatoprazole	Antihypertensive
Lomeguatribum	Antineoplastic
Nocodazole	Antineoplastic
Disuprazole	Antiulcerative
Pantoprazole	Antiulcerative
Picoprazole	Antiulcerative
Timoprazole	Antiulcerative
esomeprazole	Antiulcerative
omeprazole	Antiulcerative
Ufiprazole	Anxiolytic
Adibendan	Cardiotonic
Frentizole	Immunomodulator
Buriodyl	Unclassified

INN	INDICATION
Albendazole	Anthelminithic
Albendazole oxide	Anthelminithic
Ciclobendazole	Anthelminithic
Dribendazole	Anthelminithic
Febantel	Anthelminithic
Fenbendazole	Anthelminithic
Lobendazole	Anthelminithic
Luxabendazole	Anthelminithic
Mebendazole	Anthelminithic
Oxfendazole	Anthelminithic
Oxibendazole	Anthelminithic
Parbendazole	Anthelminithic
Tioxidazole	Anthelminithic
flubendazole	Anthelminithic
Nocodazole	Antineoplastic
Frentizole	Immunomodulator

INN	INDICATION
Albendazole oxide	Anthelminithic
Cambendazole	Anthelminithic
Mebendazole	Anthelminithic
Parbendazole	Anthelminithic
Subendazole	Anthelminithic
Tiabendazole	Anthelminithic
flubendazole	Anthelminithic
Acriflavinium Cl	Antibacterial
Eflumast	Antihistaminic
Nocodazole	Antineoplastic
Disuprazole	Antiulcerative
Pantoprazole	Antiulcerative
Timoprazole	Antiulcerative
Frentizole	Immunomodulator
Gavestinel	Nootropic
Buriodyl	Unclassified

INN	INDICATION
Xinidaminum	Analgesic
Cambendazole	Anthelminithic
Subendazole	Anthelminithic
Tiabendazole	Anthelminithic
Acriflavinium chloride	Antibacterial
Salazosulfathiazole	Antibiotic
Af 1923	Antihistaminic
Bufrolin	Antihistaminic
Eflumast	Antihistaminic
Valdecoxib	Antiinflammatory
Lomeguatribum	Antineoplastic
Nocodazole	Antineoplastic
Pyritidium bromide	Antiparasitic
Nifursol	Antiprotozoal
Timoprazole	Antiulcerative
Tolimidonum	Antiulcerative
Zolimidine	Antiulcerative
Febuxostat	Antiurolithic
Adibendan	Cardiotonic
Gavestinel	Nootropic
Buriodyl	Unclassified
Furacrinic acid	Unclassified

Figure S1. Graphical summary of the screening process using similarity to known AC colchicine-site binding agents: MI-181 in the first instance and N2G, Nocodazole, TN-16, and Plinabulin in the second. The upper filter reduces the initial database and the secondary filters are shown beneath. The table shows the names and indications of the filtered compounds ordered by indication. Antiparasitic and antineoplastic agents are highlighted in blue backgrounds, and frentizole is highlighted in yellow background.

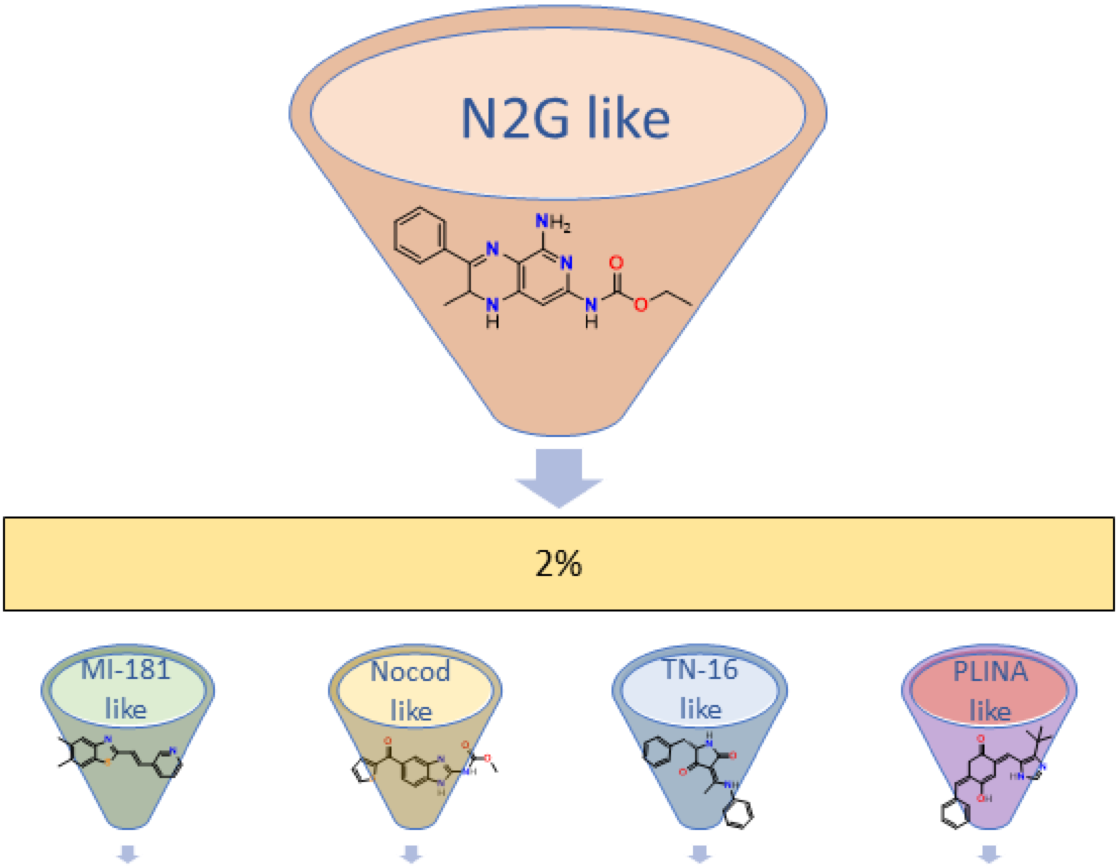

INN	INDICATION
Luxabendazole	Anthelminithic
Oxfendazole	Anthelminithic
Cambendazole	Anthelminithic
Parbendazole	Anthelminithic
flubendazole	Anthelminithic
Mebendazole	Anthelminithic
Albendazole oxide	Anthelminithic
Albendazole	Anthelminithic
Fenbendazole	Anthelminithic
Tenatoprazole	Antihypertensive
Nocodazole	Antineoplastic
Lomeguatribum	Antineoplastic
esomeprazole	Antiulcerative
Disuprazole	Antiulcerative
Picoprazole	Antiulcerative
omeprazole	Antiulcerative
Pantoprazole	Antiulcerative
Timoprazole	Antiulcerative
Ufiprazole	Anxiolytic
Adibendan	Cardiotonic
Frentizole	Immunomodulator
Buriodyl	Unclassified

INN	INDICATION
Ciclobendazole	Anthelminithic
Luxabendazole	Anthelminithic
Oxfendazole	Anthelminithic
Oxibendazole	Anthelminithic
Parbendazole	Anthelminithic
flubendazole	Anthelminithic
Mebendazole	Anthelminithic
Albendazole	Anthelminithic
Fenbendazole	Anthelminithic
Nocodazole	Antineoplastic
Frentizole	Immunomodulator

INN	INDICATION
Isonixin	Analgesic
Brinazarone	Antiarrhythmic
Farglitazar	Antidiabetic
Pioglitazone	Antidiabetic
Darglitazone	Antidiabetic
Rivoglitazone	Antidiabetic
Tetrazolast	Antihistaminic
Sudoxicam	Antiinflammatory
Isofezolac	Antiinflammatory
Meloxicam	Antiinflammatory
zolmitriptan	Antimigraine
Fosquidone	Antineoplastic
Oxmetidine	Antiulcerative
Denotivir	Antiviral
Metisazone	Antiviral
Amipizonum	Bronchodilator
Prinoxodan	Cardiotonic
Meribendan	Cardiotonic
Pimobendan	Cardiotonic
Orf-22,867	Cardiotonic
Varespladib	Cardiovascular
Meclonazepam	Sedative

INN	INDICATION
Isonixin	Analgesic
Brinazarone	Antiarrhythmic
Piromidic acid	Antibacterial
Farglitazar	Antidiabetic
Darglitazone	Antidiabetic
Tetrazolast	Antihistaminic
Sudoxicam	Antiinflammatory
Isofezolac	Antiinflammatory
Parecoxib	Antiinflammatory
Meloxicam	Antiinflammatory
Fosquidone	Antineoplastic
Oxmetidine	Antiulcerative
Denotivir	Antiviral
Metisazone	Antiviral
Amipizonum	Bronchodilator
Meribendan	Cardiotonic
Pimobendan	Cardiotonic
Orf-22,867	Cardiotonic
Meclonazepam	Sedative
Buriodyl	Unclassified

Figure S2. Graphical summary of the screening process using similarity to known AC colchicine-site binding agents: N2G in the first instance and MI-181, Nocodazole, TN-16, and Plinabulin in the second. The upper filter reduces the initial database and the secondary filters are shown beneath. The table shows the names and indications of the filtered compounds ordered by indication. Antiparasitic and antineoplastic agents are highlighted in blue backgrounds, and frentizole is highlighted in yellow background.

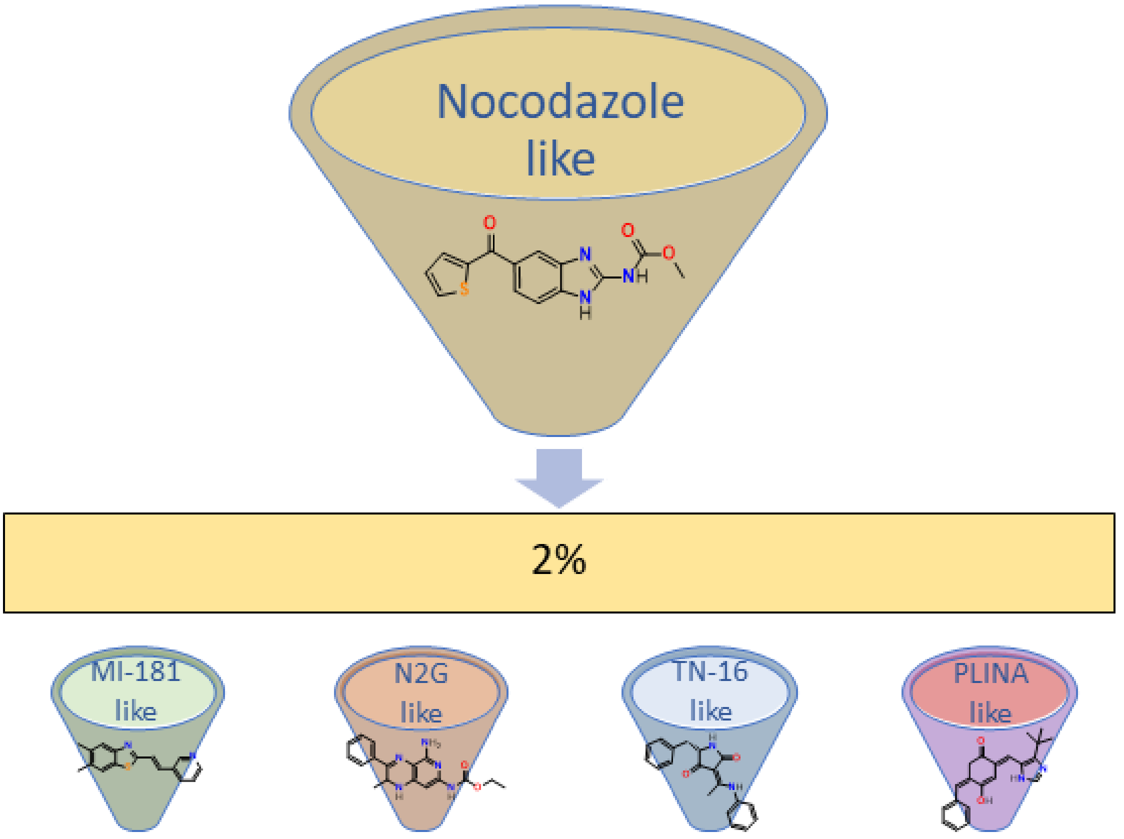

INN	INDICATION
Tioxidazole	Anthelminithic
Butoprozine	Antiarrhythmic
Brinazarone	Antiarrhythmic
Lidorestat	Antidiabetic
Acetryptinum	Antihypotensive
Timegadinum	Antiinflammatory
Tazeprofen	Antiinflammatory
Piritrexim	Antineoplastic
Nocodazole	Antineoplastic
Nictindole	Antithrombotic
Picoprazole	Antiulcerative
Enviradene	Antiviral
Divaplon	Anxiolytic
Tibenelast	Bronchodilator
Frentizole	Immunomodulator
Namirotene	Immunomodulator
Acequinoline	Phamaceutic Aid

INN	INDICATION
Etibendazole	Anthelminithic
Tioxidazole	Anthelminithic
Ciclobendazole	Anthelminithic
Dribendazole	Anthelminithic
Luxabendazole	Anthelminithic
Oxibendazole	Anthelminithic
Parbendazole	Anthelminithic
flubendazole	Anthelminithic
Mebendazole	Anthelminithic
Albendazole	Anthelminithic
Lobendazole	Anthelminithic
Rifamexil	Antibacterial
Piketoprofen	Antiinflammatory
Ilonidapum	Antiinflammatory
Timegadinum	Antiinflammatory
zolmitriptan	Antimigraine
Nocodazole	Antineoplastic
Dabigatran	Antithrombotic
Adibendan	Cardiotonic
Frentizole	Immunomodulator

INN	INDICATION
Etibendazole	Anthelminithic
Tioxidazole	Anthelminithic
Ciclobendazole	Anthelminithic
Dribendazole	Anthelminithic
Luxabendazole	Anthelminithic
Oxibendazole	Anthelminithic
Parbendazole	Anthelminithic
flubendazole	Anthelminithic
Mebendazole	Anthelminithic
Albendazole	Anthelminithic
Rifamexil	Antibacterial
Talmetoprime	Antibiotic
Bunazosin	Antihypertensive
Trimazosin	Antihypertensive
Piketoprofen	Antiinflammatory
Timegadinum	Antiinflammatory
Avitriptan	Antimigraine
zolmitriptan	Antimigraine
Trimetrexate	Antineoplastic
Nocodazole	Antineoplastic
Cinuperone	Antipsychotic
Dabigatran	Antithrombotic
Adibendan	Cardiotonic
Frentizole	Immunomodulator

INN	INDICATION
Etibendazole	Anthelminithic
Tioxidazole	Anthelminithic
Ciclobendazole	Anthelminithic
Dribendazole	Anthelminithic
Luxabendazole	Anthelminithic
Oxibendazole	Anthelminithic
Parbendazole	Anthelminithic
flubendazole	Anthelminithic
Mebendazole	Anthelminithic
Albendazole	Anthelminithic
Lobendazole	Anthelminithic
Rifamexil	Antibacterial
Talmetoprime	Antibiotic
Bunazosin	Antihypertensive
Trimazosin	Antihypertensive
Piketoprofen	Antiinflammatory
Ilonidapum	Antiinflammatory
Timegadinum	Antiinflammatory
Avitriptan	Antimigraine
zolmitriptan	Antimigraine
Trimetrexate	Antineoplastic
Nocodazole	Antineoplastic
Dabigatran	Antithrombotic
Adibendan	Cardiotonic
Frentizole	Immunomodulator

Figure S3. Graphical summary of the screening process using similarity to known AC colchicine-site binding agents: Nocodazole in the first instance and MI-181, N2G, TN-16, and Plinabulin in the second. The upper filter reduces the initial database and the secondary filters are shown beneath. The table shows the names and indications of the filtered compounds ordered by indication. Antiparasitic and antineoplastic agents are highlighted in blue backgrounds, and frentizole is highlighted in yellow background.

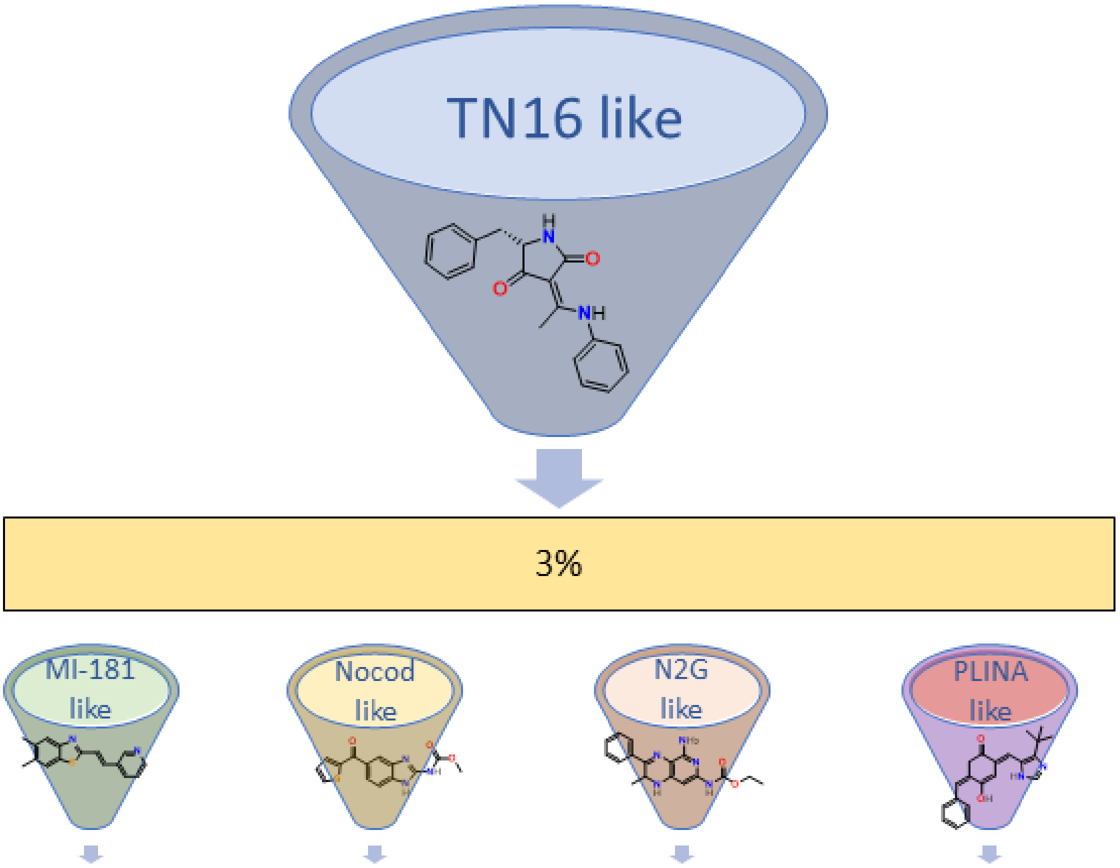

INN	INDICATION
Salverine	Analgesic
Trizoximum	Antibiotic
Anilamate	Anticonvulsant
Oxcarbazepine	Anticonvulsant
Ketimipramine	Antidepressant
Varespladib	Antihypotensive
Bendazac	Antiinflammatory
Delmetacin	Antiinflammatory
Enfenamic acid	Antiinflammatory
piroxicam	Antiinflammatory
Denotivir	Antiviral
Ampyzine	CNS stimulant
Frentizole	Immunomodulator
Roquinimex	Immunomodulator
Who no. 5198	Immunomodulator
Ac. amfonelique	Nootropic
Gavestinel	Nootropic
Bendazol	Vasodilator

INN	INDICATION
Tosifen	Antianginal
Trizoximum	Antibiotic
Anilamate	Anticonvulsant
Ketimipramine	Antidepressant
Lerisetron	Antiemetic
Mebhydrolin	Antihistaminic
Varespladib Na	Antihypotensive
Bendazac	Antiinflammatory
Isofezolac	Antiinflammatory
Oxaprozin	Antiinflammatory
Trifezolac	Antiinflammatory
piroxicam	Antiinflammatory
Oxadimedine	Antineoplastic
Denotivir	Antiviral
Ampyzine	CNS stimulant
Frentizole	Immunomodulator
Roquinimex	Immunomodulator
Who no. 5198	Immunomodulator
Ac. amfonelique	Nootropic
Gavestinel	Nootropic
Bendazol	Vasodilator

INN	INDICATION
Tosifen	Antianginal
Trizoximum	Antibiotic
Anilamate	Anticonvulsant
Talastine	Antihistaminic
Varespladib sodium	Antihypotensive
Bendazac	Antiinflammatory
Delmetacin	Antiinflammatory
Isofezolac	Antiinflammatory
Trifezolac	Antiinflammatory
piroxicam	Antiinflammatory
Bindarit	Antirheumatic
Denotivir	Antiviral
Ampyzine	CNS stimulant
Frentizole	Immunomodulator
Roquinimex	Immunomodulator
Who no. 5198	Immunomodulator
Acide amfonelique	Nootropic
Gavestinel	Nootropic
Bendazol	Vasodilator

INN	INDICATION
Dexpemedolac	Analgesic
Trizoximum	Antibiotic
Phenprocoumon	Anticoagulant
Warfarin	Anticoagulant
Imidafenacinum	Antidepressant
Varespladib Na	Antihypotensive
Bendazac	Antiinflammatory
Bufezolac	Antiinflammatory
Delmetacin	Antiinflammatory
Isofezolac	Antiinflammatory
Oxaprozin	Antiinflammatory
Trifezolac	Antiinflammatory
piroxicam	Antiinflammatory
Bindarit	Antirheumatic
Denotivir	Antiviral
Emivirine	Antiviral
Etocrilene	Dermatologic
Roquinimex	Immunomodulator
Who no. 5198	Immunomodulator
Ac. Amfonelique	Nootropic
Gavestinel	Nootropic
Denpidazonum	Unclassified

Figure S4. Graphical summary of the screening process using similarity to known AC colchicine-site binding agents: TN-16 in the first instance and MI-181, Nocodazole N2G, and Plinabulin in the second. The upper filter reduces the initial database and the secondary filters are shown beneath. The table shows the names and indications of the filtered compounds ordered by indication. Antiparasitic and antineoplastic agents are highlighted in blue backgrounds, and frentizole is highlighted in yellow background.

Table S2. SuperPred Target predictions for frentizole.

Target Name	ChEMBL-ID	UniProt ID	PDB Visualization	TTD ID	Min Activity	Assay type
Ras-related protein Rab-9A	CHEMBL1293294	P51151	1WMS	T66350	707.9 nm	Potency
Survival motor neuron protein	CHEMBL1293232	Q16637	4QQ6	T71907	891.3 nm	Potency

Target Name	ChEMBL-ID	UniProt ID	PDB Visualization	TTD ID	Probability	Model accuracy
Signal transducer and activator of transcription 1-alpha/beta	CHEMBL6101	P42224	1YVL	T64205	98%	73%
Niemann-Pick C1 protein	CHEMBL1293277	O15118	6W5S	Not Available	98%	81%
Kruppel-like factor 5	CHEMBL1293249	Q13887	Not Available	Not Available	97%	86%
Dual-specificity tyrosine-phosphorylation regulated kinase 1/	CHEMBL2292	Q13627	6S14	T92803	96%	93%
Dual specificty protein kinase CLK1	CHEMBL4224	P49759	6KHD	Not Available	95%	85,3%
Transcription intermediary factor 1-alpha	CHEMBL3108638	O15164	4YBM	Not Available	94%	96%
Vascular endothelial growth factor receptor 1	CHEMBL1868	P17948	5T89	Not Available	94%	96%
DNA-(apurinic or apyrimidinic site) lyase	CHEMBL5619	P27695	6BOW	T13348	93%	91%
Nuclear receptor ROR-beta	CHEMBL3091268	Q92753	Not Available	Not Available	91%	95,5%
Casein kinase II alpha/beta	CHEMBL3038477	P67870	6TLS	T51565	90%	99%
Glucose transporter	CHEMBL2535	P11166	6THA	Not Available	90%	99%
Nuclear factor erythroid 2-related factor 2	CHEMBL1075094	Q16236	2FLU	Not Available	89%	96%
Glycine transporter 2	CHEMBL3060	Q9Y345	Not Available	Not Available	89,4%	99%
NADPH oxidase 1	CHEMBL1287628	Q9Y5S8	Not Available	Not Available	89%	95%
Arachidonate 12-lipoxygenase	CHEMBL3687	P18054	3D3L	Not Available	89%	76%
Pregnane X receptor	CHEMBL3401	O75469	6TFI	T82702	89%	95%
Proteasome component C5	CHEMBL4208	P20618	6KWY	Not Available	88%	90%
Nuclear factor NF-kappa-B p105 subunit	CHEMBL3251	P19838	1SVC	Not Available	88%	96%
Thyroid hormone receptor alpha	CHEMBL1860	P10827	3ILZ	T79591	86%	99%
Dual specificity protein phosphatase 3	CHEMBL2635	P51452	3F81	Not Available	86%	94%
Excitatory amino acid transporter 1	CHEMBL3085	P43003	5LM4	Not Available	85%	95%
Muscarinic acetylcholine receptor M5	CHEMBL2035	P08912	6OL9	T79961	85%	95%
Platelet-derived growth factor receptor beta	CHEMBL1913	P09619	3MJG	T59102	85%	95,7%
Photoreceptor-specific nuclear receptor	CHEMBL4374	Q9Y5X4	4LOG	Not Available	84%	85%
G-protein coupled receptor 6	CHEMBL3714130	P46095	Not Available	Not Available	83,6%	97%
GABA-A receptor; alpha-1/beta-2/gamma-2	CHEMBL2095172	P14867	6X3T	T51487	83%	93%
Stem cell growth factor receptor	CHEMBL1936	P10721	2EC8	T57700	81%	84%
Galectin-3	CHEMBL4531	P17931	6FOF	T72038	81%	96,9%
Anandamide amidohydrolase	CHEMBL2243	O00519	Not Available	T11754	80%	98%
Cytochrome P450 3A4	CHEMBL340	P08684	5VCC	T37848	80%	91%
Acyl coenzyme A:cholesterol acyltransferase 1	CHEMBL2782	P35610	6P2J	Not Available	79%	92%
Ephrin type-B receptor 2	CHEMBL3290	P29323	3ZFM	T73756	79%	78%
Glutathione S-transferase Pi	CHEMBL3902	P09211	5J41	T21669	78,7%	94%
Muscarinic acetylcholine receptor M4	CHEMBL1821	P08173	5DSG	T20709	78%	94%
Platelet-derived growth factor receptor alpha	CHEMBL2007	P16234	7LBF	T53524	78%	91%
G protein-coupled receptor kinase 5	CHEMBL5678	P34947	4TND	Not Available	77%	88%
Adenosine A3 receptor	CHEMBL256	P0DMS8	Not Available	T36059	77%	96%
Cyclooxygenase-1	CHEMBL221	P23219	6Y3C	Not Available	77%	90%
Glutamate NMDA receptor; GRIN1/GRIN2B	CHEMBL1907603	Q05586	5EWM	Not Available	76%	96%
ADAM10	CHEMBL5028	O14672	6BE6	T31902	75%	97,5%
Tyrosyl-DNA phosphodiesterase 1	CHEMBL1075138	Q9NUW8	6N0D	Not Available	75%	71%
C5a anaphylatoxin chemotactic receptor	CHEMBL2373	P21730	6C1R	T15439	75%	93%
Protein-tyrosine phosphatase 2C	CHEMBL3864	Q06124	5EHR	T13057	73%	94%
Dual specificity phosphatase Cdc25C	CHEMBL2378	P30307	3OP3	Not Available	72,2%	97%
Lysosomal Pro-X carboxypeptidase	CHEMBL2335	P42785	3N2Z	Not Available	72%	100%
Kallikrein 7	CHEMBL2443	P49862	2QXI	Not Available	72%	94%
Dopamine D1 receptor	CHEMBL2056	P21728	7JVP	Not Available	72%	91%
Muscarinic acetylcholine receptor M2	CHEMBL211	P08172	5ZKC	T46185	71%	95%
Histone deacetylase 7	CHEMBL2716	Q8WUI4	3C10	Not Available	70%	89%
Calpain 1	CHEMBL3891	P07384	1ZCM	Not Available	70%	93%
Glutamate receptor ionotropic, AMPA 2	CHEMBL4016	P42262	2WJW	T42392	69%	87%
Cyclin-dependent kinase 5	CHEMBL4036	Q00535	4AU8	T20973	69%	79%
Dipeptidyl peptidase II	CHEMBL3976	Q9UHL4	4EBB	Not Available	68%	92%
Dual specificity protein kinase CLK4	CHEMBL4203	Q9HAZ1	6FYV	Not Available	68%	94%
Serine/threonine protein kinase NLK	CHEMBL5364	Q9UBE8	Not Available	Not Available	68%	79%
DNA topoisomerase I	CHEMBL1781	P11387	1K4T	T09826	67%	97%
Glycine receptor subunit alpha-1	CHEMBL5845	P23415	4X5T	T50269	67%	91%
Dihydroorotate dehydrogenase	CHEMBL1966	Q02127	6FMD	T99009	66%	96%
Protein Mdm4	CHEMBL1255126	O15151	6Q9Y	T36741	65%	90,2%
Protein-tyrosine phosphatase LC-PTP	CHEMBL2219	P35236	1ZC0	Not Available	64%	79%
Excitatory amino acid transporter 2	CHEMBL4973	P43004	Not Available	Not Available	64%	99%
Cathepsin D	CHEMBL2581	P07339	4OD9	T67102	63%	99%
Caspase-6	CHEMBL3308	P55212	2WDP	Not Available	63%	98%
Coagulation factor XIII	CHEMBL4530	P00488	4KTY	Not Available	63%	96%
Muscarinic acetylcholine receptor M1	CHEMBL216	P11229	6OIJ	T28893	62%	94%
Neuronal acetylcholine receptor; alpha4/beta4	CHEMBL1907591	P30926	6UR8	T70967	62%	100%
Cystic fibrosis transmembrane conductance regulator	CHEMBL4051	P13569	6MSM	T55654	61%	96%
Aminopeptidase N	CHEMBL1907	P15144	4FYT	T67272	61%	93%
Beta-glucosidase	CHEMBL3761	Q9HCG7	Not Available	Not Available	61%	99%
Casein kinase I delta	CHEMBL2828	P48730	6PXN	Not Available	61%	93%
Tyrosine-protein kinase FYN	CHEMBL1841	P06241	2DQ7	T17980	60%	81%
Excitatory amino acid transporter 3	CHEMBL2721	P43005	6X2L	Not Available	60,1%	93,5%
Tissue factor pathway inhibitor	CHEMBL3713062	P10646	5NMV	T78890	60%	97%
Bcr/Abl fusion protein	CHEMBL2096618	P00519	5N7E	Not Available	60%	86%
Histone deacetylase 9	CHEMBL4145	Q9UKV0	Not Available	Not Available	60%	85%
Tyrosine-protein kinase ITK/TSK	CHEMBL2959	Q08881	4HCU	T91761	60%	95%
Interleukin-23 receptor	CHEMBL4296013	Q5VWK5	5MZV	T00484	59%	88%
Activin receptor type-1B	CHEMBL5310	P36896	Not Available	Not Available	57%	70%
Nerve growth factor receptor Trk-A	CHEMBL2815	P04629	2IFG	Not Available	57%	87%
G-protein coupled bile acid receptor 1	CHEMBL5409	Q8TDU6	7CFM	T86273	57%	94%
Fatty acid synthase	CHEMBL4158	P49327	3HHD	T16514	57%	82,5%
Sodium channel protein type III alpha subunit	CHEMBL5163	Q9NY46	Not Available	T76937	57%	96,9%
Geranylgeranyl pyrophosphate synthetase	CHEMBL4769	O95749	6R4V	Not Available	56%	92%
Monoamine oxidase A	CHEMBL1951	P21397	2Z5Y	Not Available	56%	91%
Tyrosine-protein kinase ABL	CHEMBL1862	P00519	5MO4	Not Available	56%	99%
Aldose reductase	CHEMBL1900	P15121	1US0	T26623	55%	92%
Tyrosine-protein kinase BRK	CHEMBL4601	Q13882	6CZ4	T73694	55%	76%
T-cell protein-tyrosine phosphatase	CHEMBL3807	P17706	1L8K	Not Available	54%	93%
cAMP-dependent protein kinase alpha-catalytic subunit	CHEMBL4101	P17612	4WB8	Not Available	54%	83%
Endoplasmic reticulum aminopeptidase 1	CHEMBL5939	Q9NZ08	6Q4R	Not Available	54%	100%
PI3-kinase p110-alpha/p85-alpha	CHEMBL2111367	P27986	4JPS	T80276	53,9%	94%
Tyrosine-protein kinase FRK	CHEMBL4223	P42685	Not Available	Not Available	54%	71%
Proteasome Macropain subunit	CHEMBL3492	P49721	5LE5	Not Available	53%	90%
NT-3 growth factor receptor	CHEMBL5608	Q16288	6KZD	Not Available	53%	96%
MAP/microtubule affinity-regulating kinase 4	CHEMBL5754	Q96L34	5ES1	Not Available	53%	79,5%
Tyrosine-protein kinase FER	CHEMBL3982	P16591	6KC4	Not Available	53%	78%
Mitogen-activated protein kinase 7	CHEMBL5332	Q13164	4IC7	Not Available	52%	93%
Matrix metalloproteinase 12	CHEMBL4393	P39900	3BA0	Not Available	52%	92%
Tyrosine-protein kinase receptor UFO	CHEMBL4895	P30530	5U6B	T82383	52%	91%
Telomerase reverse transcriptase	CHEMBL2916	O14746	7BG9	T86052	52%	90%
Ephrin type-B receptor 3	CHEMBL4901	P54753	5L6O	Not Available	52%	87,5%
Non-receptor tyrosine-protein kinase TNK1	CHEMBL5334	Q13470	Not Available	Not Available	52%	73%
Cysteine protease ATG4B	CHEMBL1741221	Q9Y4P1	2CY7	Not Available	51,5%	87,5%
DNA topoisomerase II alpha	CHEMBL1806	P11388	6ZY5	T17048	51%	89%
Aurora kinase B/Inner centromere protein	CHEMBL3430907	Q96GD4	6YIH	T46781	51%	97,5%
Dipeptidyl peptidase VIII	CHEMBL4657	Q6V1X1	6EOP	Not Available	51%	97%
Formyl peptide receptor 1	CHEMBL3359	P21462	Not Available	T87831	51%	94%
Breakpoint cluster region protein	CHEMBL5146	P11274	5N7E	Not Available	50%	75%
Tyrosine-protein kinase receptor RET	CHEMBL2041	P07949	6Q2O	T60631	50,2%	92%
Beta amyloid A4 protein	CHEMBL2487	P05067	5BUO	T87024	50%	97%

Table S3. SwissTarget predictions for frentizole.

Target	Common name	Uniprot ID	ChEMBL ID	Target Class	Probability*	Known actives (3D/2D)
Casein kinase I delta	CSNK1D	P48730	CHEMBL2828	Kinase	0.0978745343258	0/ 48
Vascular endothelial growth factor receptor 2	KDR	P35968	CHEMBL279	Kinase	0.0978745343258	47/ 29
G protein-coupled receptor 44	PTGDR2	Q9Y5Y4	CHEMBL5071	Family A G protein- coupled re-ceptor	0.0978745343258	1088 / 0
Carnitine O- palmitoyltransferase 1, liver isoform	CPT1A	P50416	CHEMBL1293194	Enzyme	0.0978745343258	129/ 0
Carnitine palmitoyltransferase 2	CPT2	P23786	CHEMBL3238	Enzyme	0.0978745343258	49/ 0
Arachidonate 5- lipoxygenase	ALOX5	P09917	CHEMBL215	Oxidoreductase	0.0978745343258	93/ 0
Aldose reductase	AKR1B1	P15121	CHEMBL1900	Enzyme	0.0978745343258	338/ 0
Angiotensin-converting enzyme	ACE	P12821	CHEMBL1808	Protease	0.0978745343258	377/ 0
Aldehyde reductase	AKR1A1	P14550	CHEMBL2246	Enzyme	0.0978745343258	17/ 0
Lysosomal protective protein	CTSA	P10619	CHEMBL6115	Protease	0.0978745343258	370/ 0
Kelch-like ECH- associated protein 1	KEAP1	Q14145	CHEMBL2069156	Unclassified protein	0.0978745343258	10/ 0
Carnitine O- palmitoyltransferase 1, muscle isoform	CPT1B	Q92523	CHEMBL2216739	Group translocator	0.0978745343258	90/ 0
Interleukin-8 receptor B	CXCR2	P25025	CHEMBL2434	Family A G protein- coupled re-ceptor	0.0978745343258	17/ 0
Caspase-1	CASP1	P29466	CHEMBL4801	Protease	0.0978745343258	154/ 0
Prostanoid EP4 receptor	PTGER4	P35408	CHEMBL1836	Family A G protein- coupled re-ceptor	0.0978745343258	122/ 0
Prostanoid EP2 receptor	PTGER2	P43116	CHEMBL1881	Family A G protein- coupled re-ceptor	0.0978745343258	93/ 0
Integrin alpha-V/beta-3	ITGAV ITGB3	P06756 P05106	CHEMBL1907598	Membrane receptor	0.0978745343258	164/ 0
Prostanoid EP3 receptor	PTGER3	P43115	CHEMBL3710	Family A G protein- coupled re-ceptor	0.0978745343258	39/ 0
Leukocyte elastase	ELANE	P08246	CHEMBL248	Protease	0.0978745343258	22/ 0
5-lipoxygenase activating protein	ALOX5AP	P20292	CHEMBL4550	Other cytosolic protein	0.0978745343258	139/ 0
Neprilysin	MME	P08473	CHEMBL1944	Protease	0.0978745343258	294/ 0
Induced myeloid leukemia cell differentiation protein Mcl-1	MCL1	Q07820	CHEMBL4361	Other cytosolic protein	0.0978745343258	123/ 0
Apoptosis regulator Bcl-2	BCL2	P10415	CHEMBL4860	Other ion channel	0.0978745343258	48/ 0
Endothelin receptor ET- B	EDNRB	P24530	CHEMBL1785	Family A G protein- coupled re-ceptor	0.0978745343258	149/ 0
Integrin alpha-4/beta-1	ITGB1 ITGA4	P05556 P13612	CHEMBL1907599	Membrane receptor	0.0978745343258	575/ 0
Insulin-like growth factor I receptor	IGF1R	P08069	CHEMBL1957	Kinase	0.0978745343258	12/ 0
Peroxisome activated receptor gamma	PPARG	P37231	CHEMBL235	Nuclear receptor	0.0978745343258 728/ 0 proliferator-	
Peroxisome proliferator-acti-vated receptor alpha	PPARA	Q07869	CHEMBL239	Nuclear receptor	0.0978745343258	498/ 0
Endothelin receptor ET- A	EDNRA	P25101	CHEMBL252	Family A G protein- coupled re-ceptor	0.0978745343258	442/ 0
L-lactate dehydrogenase A chain	LDHA	P00338	CHEMBL4835	Enzyme	0.0978745343258	48/ 0
Liver glycogen phosphory-lase	PYGL	P06737	CHEMBL2568	Enzyme	0.0978745343258	116/ 0
Peroxisome proliferator-activa-ted receptor delta	PPARD	Q03181	CHEMBL3979	Nuclear receptor	0.0978745343258	145/ 0
Cholecystokinin B receptor	CCKBR	P32239	CHEMBL298	Family A G protein- coupled re-ceptor	0.0978745343258	201/ 0
Chymase	CMA1	P23946	CHEMBL4068	Protease	0.0978745343258	113/ 0
Cathepsin G	CTSG	P08311	CHEMBL4071	Protease	0.0978745343258	13/ 0
ADAMTS5	ADAMTS5	Q9UNA0	CHEMBL2285	Protease	0.0978745343258	96/ 0
Squalene synthetase (by ho-mology)	FDFT1	P37268	CHEMBL3338	Enzyme	0.0978745343258	50/ 0
Phospholipase A2 group IIA	PLA2G2A	P14555	CHEMBL3474	Enzyme	0.0978745343258	61/ 0
Thromboxane A2 receptor	TBXA2R	P21731	CHEMBL2069	Family A G protein- coupled re-ceptor	0.0978745343258	259/ 0
Integrin alpha-4/beta-7	ITGB7 ITGA4	P26010 P13612	CHEMBL2095184	Membrane receptor	0.0978745343258	228/ 0
Matrix metalloproteinase 9	MMP9	P14780	CHEMBL321	Protease	0.0978745343258	142/ 0
Matrix metalloproteinase 2	MMP2	P08253	CHEMBL333	Protease	0.0978745343258	255/ 0
Angiotensin-converting enzyme 2	ACE2	Q9BYF1	CHEMBL3736	Protease	0.0978745343258	38/ 0
Prostanoid DP receptor	PTGDR	Q13258	CHEMBL4427	Family A G protein- coupled re-ceptor	0.0978745343258	162/ 0
Phosphomannomutase 2	PMM2	O15305	CHEMBL1741162	Enzyme	0.0978745343258	1/ 0
Vitronectin receptor alpha	ITGAV	P06756	CHEMBL3660	Membrane receptor	0.0978745343258	30/ 0

Free fatty acid receptor 1	FFAR1	O14842	CHEMBL4422	Family A G protein- coupled receptor	0.0978745343258	100/ 0
Phosphoethanolamine/ phosphocholine phosphatase	PHOSPHO1	Q8TCT1	CHEMBL6113	Enzyme	0.0978745343258	1/ 0
Carboxypeptidase A1	CPA1	P15085	CHEMBL2088	Protease	0.0978745343258	19/ 0
Protein farnesyltransferase	FNTA FNTB	P49354 P49356	CHEMBL2094108	Enzyme	0.0978745343258	75/ 0
Peptidyl-prolyl cis-trans isomerase NIMA- interacting 1	PIN1	Q13526	CHEMBL2288	Enzyme	0.0978745343258	54/ 0
Prostanoid EP1 receptor	PTGER1	P34995	CHEMBL1811	Family A G protein- coupled receptor	0.0978745343258	326/ 0
Integrin alpha-4	ITGA4	P13612	CHEMBL278	Membrane receptor	0.0978745343258	66/ 0
Matrix metalloproteinase 1	MMP1	P03956	CHEMBL332	Protease	0.0978745343258	85/ 0
ADAM17	ADAM17	P78536	CHEMBL3706	Protease	0.0978745343258	52/ 0
Matrix metalloproteinase 14	MMP14	P50281	CHEMBL3869	Protease	0.0978745343258	60/ 0
Thrombin and coagulation factor X	F10	P00742	CHEMBL244	Protease	0.0978745343258	37/ 0
Bromodomain- containing protein 4	BRD4	O60885	CHEMBL1163125	Reader	0.0978745343258	9/ 0
Cholecystokinin A receptor	CCKAR	P32238	CHEMBL1901	Family A G protein- coupled receptor	0.0978745343258	65/ 0
Cyclophilin A	PPIA	P62937	CHEMBL1949	Isomerase	0.0978745343258	2/ 0
Glyoxalase I	GLO1	Q04760	CHEMBL2424	Enzyme	0.0978745343258	1/ 0
Serine/threonine- protein kinase PLK1	PLK1	P53350	CHEMBL3024	Kinase	0.0978745343258	4/ 0
Matrix metalloproteinase 12	MMP12	P39900	CHEMBL4393	Protease	0.0978745343258	69/ 0
Matrix metalloproteinase 8	MMP8	P22894	CHEMBL4588	Protease	0.0978745343258	126/ 0
Endothelin-converting enzyme 1	ECE1	P42892	CHEMBL4791	Protease	0.0978745343258	131/ 0
Cyclooxygenase-2	PTGS2	P35354	CHEMBL230	Oxidoreductase	0.0978745343258	110/ 0
Serum albumin	ALB	P02768	CHEMBL3253	Secreted protein	0.0978745343258	4/ 0
Prostaglandin E synthase	PTGES	O14684	CHEMBL5658	Enzyme	0.0978745343258	96/ 0
Group IIE secretory phospholipase A2	PLA2G2E	Q9NZK7	CHEMBL2154	Enzyme	0.0978745343258	8/ 0
Lysine-specific demethylase 5A	KDM5A	P29375	CHEMBL2424504	Eraser	0.0978745343258	7/ 0
Dual specificity protein phosphatase 3	DUSP3	P51452	CHEMBL2635	Phosphatase	0.0978745343258	16/ 0
Matrix metalloproteinase 13	MMP13	P45452	CHEMBL280	Protease	0.0978745343258	142/ 0
Matrix metalloproteinase 3	MMP3	P08254	CHEMBL283	Protease	0.0978745343258	149/ 0
Lysine-specific demethylase 5B	KDM5B	Q9UGL1	CHEMBL3774295	Eraser	0.0978745343258	6/ 0
Lysine-specific demethylase 2B	KDM2B	Q8NHM5	CHEMBL3779760	Eraser	0.0978745343258	3/ 0
Matrix metalloproteinase 7	MMP7	P09237	CHEMBL4073	Protease	0.0978745343258	23/ 0
Bradykinin B1 receptor	BDKRB1	P46663	CHEMBL4308	Family A G protein- coupled receptor	0.0978745343258	4/ 0
Phospholipase A2 group V	PLA2G5	P39877	CHEMBL4323	Enzyme	0.0978745343258	6/ 0
Group X secretory phospholipase A2	PLA2G10	O15496	CHEMBL4342	Enzyme	0.0978745343258	10/ 0
Phospholipase A2 group 1B	PLA2G1B	P04054	CHEMBL4426	Enzyme	0.0978745343258	26/ 0
Lysine-specific demethylase 4C	KDM4C	Q9H3R0	CHEMBL6175	Eraser	0.0978745343258	16/ 0
Phosphodiesterase 4D	PDE4D	Q08499	CHEMBL288	Phosphodiesterase	0.0978745343258	78/ 0
PI3-kinase p110-beta subunit	PIK3CB	P42338	CHEMBL3145	Enzyme	0.0978745343258	20/ 0
Voltage-gated N-type calcium channel alpha-1B subunit	CACNA1B	Q00975	CHEMBL4478	Voltage-gated ion channel	0.0978745343258	7/ 0
Fatty acid binding protein adipocyte	FABP4	P15090	CHEMBL2083	Fatty acid binding protein family	0.0978745343258	49/ 0
Glycogen synthase kinase-3 alpha	GSK3A	P49840	CHEMBL2850	Kinase	0.0978745343258	16/ 0
Fatty acid binding protein epidermal	FABP5	Q01469	CHEMBL3674	Fatty acid binding protein family	0.0978745343258	10/ 0
Leukotriene B4 receptor 1	LTB4R	Q15722	CHEMBL3911	Family A G protein- coupled receptor	0.0978745343258	62/ 0
Aldo-keto-reductase family 1 member C3	AKR1C3	P42330	CHEMBL4681	Enzyme	0.0978745343258	107/ 0
Solute carrier family 22 member 12	SLC22A12	Q96S37	CHEMBL6120	Electrochemical transporter	0.0978745343258	51/ 0
Bile acid receptor FXR	NR1H4	Q96RI1	CHEMBL2047	Nuclear receptor	0.0978745343258	58/ 0
Neurokinin 3 receptor	TACR3	P29371	CHEMBL4429	Family A G protein- coupled receptor	0.0978745343258	3/ 0
Glutamate [NMDA] receptor PROTEIN	GRIN1	Q05586	CHEMBL2015	Ligand-gated ion channel	0.0978745343258	21/ 0
Gamma-secretase	PSEN2 PSENEN NCSTN APH1A PSEN1 APH1B	P49810 Q9NZ42 Q92542 Q96BI3 P49768 Q8WW43	CHEMBL2094135	Protease	0.0978745343258	12/ 0
Cyclooxygenase-1	PTGS1	P23219	CHEMBL221	Oxidoreductase	0.0978745343258	32/ 0
Type-1 angiotensin II receptor (by homology)	AGTR1	P30556	CHEMBL227	Family A G protein- coupled receptor	0.0978745343258	205/ 0
Serotonin transporter	SLC6A4	P31645	CHEMBL228	Electrochemical transporter	0.0978745343258	3/ 0
Bone morphogenetic protein 1	BMP1	P13497	CHEMBL3898	Protease	0.0978745343258	18/ 0
Group IIF secretory phospholipase A2	PLA2G2F	Q9BZM2	CHEMBL4278	Enzyme	0.0978745343258	5/ 0
Group IID secretory phospholipase A2	PLA2G2D	Q9UNK4	CHEMBL4281	Enzyme	0.0978745343258	5/ 0

Table S4. Chemical Checker predictions for frentizole.

Frentizole

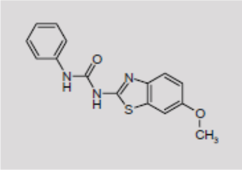

InChIKey
JHBWYQRKOUBPCA-UHFFFAOYSA-N (https://www.ebi.ac.uk/unichem/frontpage/results?queryText=JHBWYQRKOUBPCA-UHFFFAOYSA-N&kind=InChIKey&sources=&incl=exclude)

Name
Frentizole

PubChem CID
33334 (https://pubchem.ncbi.nlm.nih.gov/compound/33334)

Molecular Formula
$C_{15}H_{13}N_3O_2S$

Molecular Weight
299.36 g/mol

Rule of 5 Violations
0

Chemical Beauty
0.567

Singularity
0.356

Popularity
0.218

Mappability
0.078

Targets
RELA (https://www.uniprot.org/uniprot/Q04206), MAPT (https://www.uniprot.org/uniprot/P10636), KMT2A (https://www.uniprot.org/uniprot/Q03164), MEN1 (https://www.uniprot.org/uniprot/O00255), NFKB1 (https://www.uniprot.org/uniprot/P19838), NFKB2 (https://www.uniprot.org/uniprot/Q00653), NPC1 (https://www.uniprot.org/uniprot/O15118), RAB9A (https://www.uniprot.org/uniprot/P51151), SMN1 (https://www.uniprot.org/uniprot/Q16637), TDP1 (https://www.uniprot.org/uniprot/Q9NUW8), P08659 (https://www.uniprot.org/uniprot/P08659), ENSG00000284190 (https://www.uniprot.org/uniprot/ENSG00000284190), Rorc (https://www.uniprot.org/uniprot/P51450)
Less

Table S5. Similarity ensemble approach (SEA) predictions for frentizole.

Target ID	Affinity Threshold (nM)	P-Value	Max Tc	Cut Sum	Z-Score	Name	Description
HCD2_HUMAN	5	8,637E-102	5085	30068	1863773	HSD17B10	3-hydroxyacyl-CoA dehydrogenase type-2
C3SLN3_ECOLX	5	7,586E-93	4000	63217	1703206	gyrB	DNA gyrase subunit B
P2RY1_HUMAN	5	1,398E-86	4444	434447	1590722	P2RY1	P2Y purinoceptor 1
NMT_CANAL	5	1,811E-76	4583	106540	1409170	NMT1	Glycylpeptide N-tetradecanoyltransferase
LUCI_PHOPE	5	7,839E-67	5556	58324	1236167		Luciferin 4-monooxygenase
PORCN_MOUSE	5	2,682E-65	5152	120716	1208625	Porcn	Protein-serine O-palmitoleoyltransferase porcupine
KCNK9_HUMAN	5	6,567E-59	4600	55335	1093923	KCNK9	Potassium channel subfamily K member 9
Q9FBC5_STREE	5	7,668E-59	6034	40623	1092714	fabK	Enoyl-ACP reductase II
ISPD_ARATH	5	4,134E-57	6296	11890	1061624	ISPD	2-C-methyl-D-erythritol 4-phosphate cytidylyltransferase, chloroplastic
LIPL_RAT	5	4,466E-57	3333	24392	1061023	Lpl	Lipoprotein lipase
LUCI_PHOPY	5	2,048E-54	5849	289155	1013241		Luciferin 4-monooxygenase
SENP7_HUMAN	5	7,485E-54	5439	295710	1003137	SENP7	Sentrin-specific protease 7
FPRS1_MOUSE	5	6,092E-51	3000	23047	950883	Fpr-s1	Formyl peptide receptor-related sequence 1
C3SLB7_ECOLX	5	4,718E-49	3284	27510	916968	glmU	Bifunctional protein GlmU
NCS1_HUMAN	5	3,751E-48	4074	4074	900803	NCS1	Neuronal calcium sensor 1
Q9VWX8_DROME	5	3,751E-48	4074	4074	900803	Frq2	FI18190p1
SENP8_HUMAN	5	4,948E-48	5263	147997	898644	SENP8	Sentrin-specific protease 8
DYR1A_RAT	5	5,7E-48	6078	345940	861636	Dyrk1a	Dual specificity tyrosine-phosphorylation-regulated kinase 1A
AK1C2_HUMAN	5	9,688E-48	4038	86298	893406	AKR1C2	Aldo-keto reductase family 1 member C2
KC1D_HUMAN	5	1,12E-47	6863	277144	874324	CSNK1D	Casein kinase I isoform delta
PTPRE_HUMAN	5	3,304E-45	3231	9502	847933	PTPRE	Receptor-type tyrosine-protein phosphatase epsilon
BLM_HUMAN	5	7,694E-45	3167	29653	841343	BLM	Bloom syndrome protein
TESK2_HUMAN	5	3,141E-44	3194	12136	830374	TESK2	Dual specificity testis-specific protein kinase 2
GYRB_MYCSM	5	5,95E-43	3939	84564	789488	gyrB	DNA gyrase subunit B
SENP6_HUMAN	5	7,402E-42	5263	153388	787785	SENP6	Sentrin-specific protease 6
EPHB_MYCTO	5	1,133E-41	4146	7284	784468		Epoxide hydrolase B
Q50565_METTH	5	3,537E-41	3509	3509	775589		Carbonic anhydrase
LCK_HUMAN	5	8,084E-41	5893	458588	769144	LCK	Tyrosine-protein kinase Lck
GP139_HUMAN	5	2,18E-36	3279	25039	671646	GPR139	Probable G-protein coupled receptor 139
BAP1_HUMAN	5	4,067E-36	3099	3099	684734	BAP1	Ubiquitin carboxyl-terminal hydrolase BAP1
LIPE_HUMAN	5	1,502E-35	3836	225033	674548	LIPG	Endothelial lipase
AA3R_HUMAN	5	1,616E-32	5283	594635	620118	ADORA3	Adenosine receptor A3
STK39_RAT	5	1,616E-32	4286	10038	620118	Stk39	STE20/SPS1-related proline-alanine-rich protein kinase
A0A0C7ACN7_PSEAI	5	1,126E-31	3387	16143	604982	pqsD	3-oxoacyl-ACP synthase
DPOL_HHV6U	5	1,147E-31	4219	13169	604837	U38	DNA polymerase catalytic subunit
VGFR2_HUMAN	5	1,644E-31	5000	987045	602031	KDR	Vascular endothelial growth factor receptor 2
CDK3_HUMAN	5	4,447E-31	4028	55513	594274	CDK3	Cyclin-dependent kinase 3
PPIA_HUMAN	5	5,735E-31	3485	58528	592290	PPIA	Peptidyl-prolyl cis-trans isomerase A
MTR1A_HUMAN	5	1,17E-30	3455	219664	568776	MTNR1A	Melatonin receptor type 1A
MCHR1_MOUSE	5	4,123E-30	3380	41957	576909	Mchr1	Melanin-concentrating hormone receptor 1
STAT1_HUMAN	5	5,129E-30	5472	55837	575207	STAT1	Signal transducer and activator of transcription 1-alpha/beta
CLK1_HUMAN	5	5,984E-30	5333	133206	574005	CLK1	Dual specificity protein kinase CLK1
PGFRA_HUMAN	5	1,553E-29	4407	169621	566567	PDGFRA	Platelet-derived growth factor receptor alpha
KCNJ6_HUMAN	5	3,725E-29	4194	53686	559748	KCNJ6	G protein-activated inward rectifier potassium channel 2
CYSP_PLAFA	5	6,947E-29	3333	27746	554888		Trophozoite cysteine proteinase
KC1D_RAT	5	1,051E-28	3457	22189	551657	Csnk1d	Casein kinase I isoform delta
NEDD4_HUMAN	5	4,15E-28	3729	3729	522999	NEDD4	E3 ubiquitin-protein ligase NEDD4
CAH2_HUMAN	5	1,461E-27	4286	654269	531140	CA2	Carbonic anhydrase 2
AK1C3_HUMAN	5	1,577E-27	4038	125973	530545	AKR1C3	Aldo-keto reductase family 1 member C3
ABCG2_HUMAN	5	6,89E-27	3810	139808	501094	ABCG2	Broad substrate specificity ATP-binding cassette transporter ABCG2
Q9GME2_CALJA	5	3,775E-26	3607	3607	505785	HSD17B1	17-beta-hydroxysteroid dehydrogenase type 1
PLCG2_HUMAN	5	7,137E-26	5172	11559	500818	PLCG2	1-phosphatidylinositol 4,5-bisphosphate phosphodiesterase gamma-2
HIS1_MYCTU	5	8,786E-26	3676	6489	499198	hisG	ATP phosphoribosyltransferase
RAF1_HUMAN	5	1,714E-25	5000	253481	493985	RAF1	RAF proto-oncogene serine/threonine-protein kinase
PPIA_RAT	5	2,075E-25	3621	13162	492496	Ppia	Peptidyl-prolyl cis-trans isomerase A
TIE2_HUMAN	5	5,602E-25	5000	163137	484754	TEK	Angiopoietin-1 receptor
CAH4_BOVIN	5	1,202E-24	3750	222108	478799	CA4	Carbonic anhydrase 4
CAH1_HUMAN	5	1,584E-24	4118	519999	476649	CA1	Carbonic anhydrase 1
PTGES_MOUSE	5	2,144E-24	3226	6167	474288	Ptges	Prostaglandin E synthase
MTR1B_HUMAN	5	2,192E-24	3929	169550	474116	MTNR1B	Melatonin receptor type 1B
PDGFB_HUMAN	5	3,847E-24	3906	15664	469732	PDGFB	Platelet-derived growth factor subunit B
KC1A_BOVIN	5	7,184E-23	3188	3188	446907	CSNK1A1	Casein kinase I isoform alpha
KCNJ3_HUMAN	5	1,246E-22	4194	53686	442616	KCNJ3	G protein-activated inward rectifier potassium channel 1
MECP2_HUMAN	5	1,469E-22	4107	4107	441331	MECP2	Methyl-CpG-binding protein 2
PTN7_HUMAN	5	3,361E-22	4407	74104	434877	PTPN7	Tyrosine-protein phosphatase non-receptor type 7
Q38C41_TRYB2	5	7,237E-22	3607	31916	428897		Phosphotransferase
PRIO_SHEEP	5	8,424E-22	3333	12426	427713	PRNP	Major prion protein
KPYK_STAAR	5	1,05E-21	4561	34220	408043	pyk	Pyruvate kinase
Q6FTL6_CANGA	5	1,162E-21	3922	34518	425205	NCE103	Carbonic anhydrase

Target ID	Affinity Threshold (nM)	P-Value	Max Tc	Cut Sum	Z-Score	Name	Description
MYOC_HUMAN	5	4,21E-21	0.2963	0.2963	415.164	MYOC	Myocilin
MK03_HUMAN	5	5,26E-21	0.3200	35.467	413.436	MAPK3	Mitogen-activated protein kinase 3
CP1A2_HUMAN	5	6,78E-21	0.7045	481.041	411.452	CYP1A2	Cytochrome P450 1A2
B2CL1_HUMAN	5	9,82E-21	0.3820	164.680	408.563	BCL2L1	Bcl-2-like protein 1
B4EB35_BURCJ	5	1,44E-20	0.2895	0.2895	405.557	hldA	D-beta-D-heptose 7-phosphate kinase
CP2CJ_HUMAN	5	3,58E-20	0.5179	464.663	398.474	CYP2C19	Cytochrome P450 2C19
CASP3_HUMAN	5	4,64E-20	0.5263	188.483	396.455	CASP3	Caspase-3
ACRO_HUMAN	5	7,21E-20	0.3284	21.366	393.023	ACR	Acrosin
Q862F3_BOVIN	5	1,36E-19	0.3143	20.783	388.049		Similar to alpha-tubulin isoform 1
STRP_STRP1	5	2,20E-19	0.5472	311.649	384.308	ska	Streptokinase A
Q8IL11_PLAF7	5	2,60E-19	0.3750	69.097	383.031		M17 leucyl aminopeptidase
AURKB_HUMAN	5	2,92E-19	0.5536	269.351	382.107	AURKB	Aurora kinase B
HYEP_HUMAN	5	3,18E-19	0.4146	62.767	381.454	EPHX1	Epoxide hydrolase 1
Q25615_ONCVO	5	8,34E-19	0.3333	18.789	373.931		Chitinase
EBNA1_EBVB9	5	5,30E-18	0.3710	22.127	359.518	EBNA1	Epstein-Barr nuclear antigen 1
HIPK4_HUMAN	5	5,48E-18	0.5536	66.831	359.251	HIPK4	Homeodomain-interacting protein kinase 4
KCNJ5_HUMAN	5	1,17E-17	0.4062	39.954	353.344	KCNJ5	G protein-activated inward rectifier potassium channel 4
CSK22_HUMAN	5	1,19E-17	0.3667	62.925	353.219	CSNK2A2	Casein kinase II subunit alpha'
DHB1_HUMAN	5	2,19E-17	0.4259	82.950	348.435	HSD17B1	Estradiol 17-beta-dehydrogenase 1
AHK3_ARATH	5	2,20E-17	0.3922	0.3922	348.424	AHK3	Histidine kinase 3
TBB2B_BOVIN	5	2,20E-17	0.3571	50.609	348.401	TUBB2B	Tubulin beta-2B chain
HPSE_HUMAN	5	2,22E-17	0.3485	42.859	348.353	HPSE	Heparanase
WRN_HUMAN	5	2,45E-17	0.3167	0.6167	347.590	WRN	Werner syndrome ATP-dependent helicase
KLF5_HUMAN	5	3,00E-17	0.5849	35.347	346.003	KLF5	Krueppel-like factor 5
NR2E3_HUMAN	5	3,85E-17	0.4364	38.150	344.055	NR2E3	Photoreceptor-specific nuclear receptor
CAH12_HUMAN	5	9,55E-17	0.4118	268.509	336.970	CA12	Carbonic anhydrase 12
TIM23_YEAST	5	1,48E-16	0.5849	64.373	333.532	TIM23	Mitochondrial import inner membrane translocase subunit TIM23
ITK_HUMAN	5	1,60E-16	0.6296	122.821	332.952	ITK	Tyrosine-protein kinase ITK/TSK
ROCK1_HUMAN	5	1,76E-16	0.3636	174.124	332.219	ROCK1	Rho-associated protein kinase 1
KIT_HUMAN	5	3,33E-16	0.4286	170.695	327.236	KIT	Mast/stem cell growth factor receptor Kit
ENPP2_MOUSE	5	3,42E-16	0.3125	0.9157	327.020	Enpp2	Ectonucleotide pyrophosphatase/phosphodiesterase family member 2
SENP1_HUMAN	5	5,54E-16	0.3286	0.9421	323.267	SENP1	Sentrin-specific protease 1
VGFR3_HUMAN	5	6,96E-16	0.3529	104.018	321.486	FLT4	Vascular endothelial growth factor receptor 3
SRPK1_HUMAN	5	7,35E-16	0.3538	48.962	321.059	SRPK1	SRSF protein kinase 1
DHB2_HUMAN	5	1,02E-15	0.4259	80.125	318.481	HSD17B2	Estradiol 17-beta-dehydrogenase 2
HYEP_MOUSE	5	1,15E-15	0.3704	37.253	317.580	Ephx1	Epoxide hydrolase 1
RYR2_HUMAN	5	1,21E-15	0.3871	19.077	317.203	RYR2	Ryanodine receptor 2
PYRD_HUMAN	5	2,67E-15	0.5769	100.677	311.015	DHODH	Dihydroorotate dehydrogenase (quinone), mitochondrial
PK3CG_HUMAN	5	3,19E-15	0.5200	221.745	309.604	PIK3CG	Phosphatidylinositol 4,5-bisphosphate 3-kinase catalytic subunit gamma isoform
TRPM7_MOUSE	5	3,62E-15	0.2879	0.2879	308.638	Trpm7	Transient receptor potential cation channel subfamily M member 7
LIPC_HUMAN	5	3,86E-15	0.3553	65.641	308.133	LIPC	Hepatic triacylglycerol lipase
GLSK_HUMAN	5	5,16E-15	0.3770	96.483	305.856	GLS	Glutaminase kidney isoform, mitochondrial
LYOX_HUMAN	5	5,39E-15	0.3433	0.9588	305.521	LOX	Protein-lysine 6-oxidase
PK3CA_HUMAN	5	6,87E-15	0.5246	403.290	303.630	PIK3CA	Phosphatidylinositol 4,5-bisphosphate 3-kinase catalytic subunit alpha isoform
TAOK3_HUMAN	5	9,22E-15	0.5397	29.621	301.340	TAOK3	Serine/threonine-protein kinase TAO3
PORCN_HUMAN	5	1,64E-14	0.5000	19.464	296.873	PORCN	Protein-serine O-palmitoleoyltransferase porcupine
PGLD_CAMJE	5	1,72E-14	0.3188	14.957	296.499	pglD	UDP-N-acetylbacillosamine N-acetyltransferase
LUXN_VIBHA	5	1,88E-14	0.3333	0.3333	295.772	luxN	Autoinducer 1 sensor kinase/phosphatase LuxN
CASR_HUMAN	5	1,93E-14	0.4857	85.521	295.583	CASR	Extracellular calcium-sensing receptor
FLT3_HUMAN	5	2,23E-14	0.3846	207.967	294.443	FLT3	Receptor-type tyrosine-protein kinase FLT3
AA1R_HUMAN	5	3,90E-14	0.4286	306.413	290.094	ADORA1	Adenosine receptor A1
ABL1_HUMAN	5	4,59E-14	0.5849	209.698	288.826	ABL1	Tyrosine-protein kinase ABL1
ILK_HUMAN	5	4,90E-14	0.3793	37.366	288.314	ILK	Integrin-linked protein kinase
C4YTQ8_CANAW	5	3,33E-13	0.5000	116.114	272.619		ATP-dependent molecular chaperone HSP82
KCNQ1_HUMAN	5	3,33E-13	0.3239	11.890	273.862	KCNQ1	Potassium voltage-gated channel subfamily KQT member 1
CAN_CANAL	5	4,44E-13	0.3922	31.185	271.089	NCE103	Carbonic anhydrase
LUCI_RENRE	5	4,44E-13	0.3125	0.5946	271.053		Coelenterazine h 2-monooxygenase
ANDR_MOUSE	5	5,55E-13	0.3684	19.648	269.121	Ar	Androgen receptor
VGFR1_HUMAN	5	5,55E-13	0.4286	119.751	270.054	FLT1	Vascular endothelial growth factor receptor 1
AHK4_ARATH	5	7,77E-13	0.3922	0.3922	266.316	AHK4	Histidine kinase 4
CP3A4_HUMAN	5	7,77E-13	0.4306	286.801	266.422	CYP3A4	Cytochrome P450 3A4
DYR1A_MOUSE	5	7,77E-13	0.4737	0.4737	266.233	Dyrk1a	Dual specificity tyrosine-phosphorylation-regulated kinase 1A
MITF_HUMAN	5	7,77E-13	0.4800	81.886	266.286	MITF	Microphthalmia-associated transcription factor
PPAP_HUMAN	5	7,77E-13	0.3103	0.6207	267.163	ACP3	Prostatic acid phosphatase
MCL1_HUMAN	5	8,88E-13	0.4746	152.634	265.766	MCL1	Induced myeloid leukemia cell differentiation protein Mcl-1
CSK21_HUMAN	5	1,22E-12	0.3667	99.878	262.971	CSNK2A1	Casein kinase II subunit alpha
ETHR_MYCTU	5	1,55E-12	0.4286	13.894	261.603	ethR	HTH-type transcriptional regulator EthR
5HT3A_CAVPO	5	1,89E-12	0.4000	13.375	260.048	HTR3A	5-hydroxytryptamine receptor 3A
TAOK1_HUMAN	5	2,11E-12	0.5397	63.446	259.039	TAOK1	Serine/threonine-protein kinase TAO1
A4_HUMAN	5	2,89E-12	0.4528	103.346	256.635	APP	Amyloid-beta precursor protein
FPR2_HUMAN	5	3,11E-12	0.4615	70.893	255.834	FPR2	N-formyl peptide receptor 2
GRM5_MOUSE	5	3,44E-12	0.2879	0.2879	255.087	Grm5	Metabotropic glutamate receptor 5
TRPC6_HUMAN	5	4,00E-12	0.3188	0.9068	253.980	TRPC6	Short transient receptor potential channel 6
DYR1A_HUMAN	5	5,11E-12	0.6957	111.637	252.002	DYRK1A	Dual specificity tyrosine-phosphorylation-regulated kinase 1A
BRAF_HUMAN	5	1,09E-11	0.5000	276.351	246.220	BRAF	Serine/threonine-protein kinase B-raf
CTDS1_HUMAN	5	1,37E-11	0.4182	89.770	244.430	CTDSP1	Carboxy-terminal domain RNA polymerase II polypeptide A small phosphatase 1
SMN_HUMAN	5	1,49E-11	0.3898	10.379	243.727	SMN1; SMN2	Survival motor neuron protein
AOFB_MOUSE	5	2,05E-11	0.3000	0.5917	241.222	Maob	Amine oxidase [flavin-containing] B
CCKAR_RAT	5	2,28E-11	0.3582	90.417	240.415	Cckar	Cholecystokinin receptor type A
CCNE1_HUMAN	5	2,30E-11	0.4028	68.160	240.354	CCNE1	G1/S-specific cyclin-E1
PGFRB_HUMAN	5	2,71E-11	0.4286	121.538	239.076	PDGFRB	Platelet-derived growth factor receptor beta
BLK_HUMAN	5	5,31E-11	0.3333	45.255	233.831	BLK	Tyrosine-protein kinase Blk
ALR_HUMAN	5	5,49E-11	0.5490	122.610	233.572	GFER	FAD-linked sulfhydryl oxidase ALR
GHSR_HUMAN	5	7,37E-11	0.6604	139.650	231.269	GHSR	Growth hormone secretagogue receptor type 1
CKX2_ARATH	5	8,12E-11	0.3016	0.3016	230.514	CKX2	Cytokinin dehydrogenase 2
NEK4_HUMAN	5	8,89E-11	0.3333	31.002	229.808	NEK4	Serine/threonine-protein kinase Nek4
TRPC3_HUMAN	5	1,13E-10	0.3188	0.9068	227.967	TRPC3	Short transient receptor potential channel 3
FES_HUMAN	5	1,30E-10	0.5536	20.634	226.867	FES	Tyrosine-protein kinase Fes/Fps

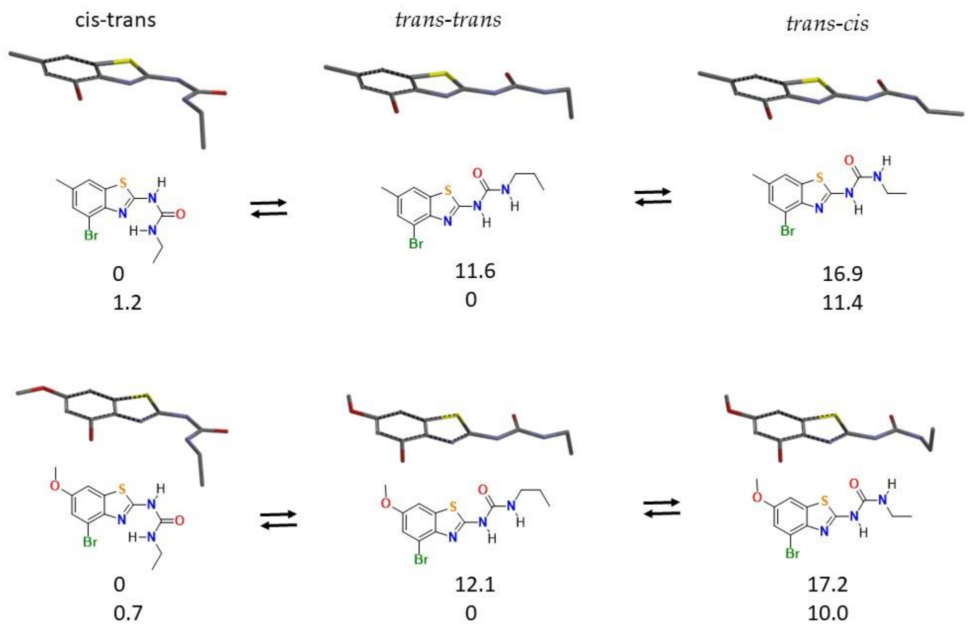

Figure S5. Configurations of the amide bonds in ethylureas

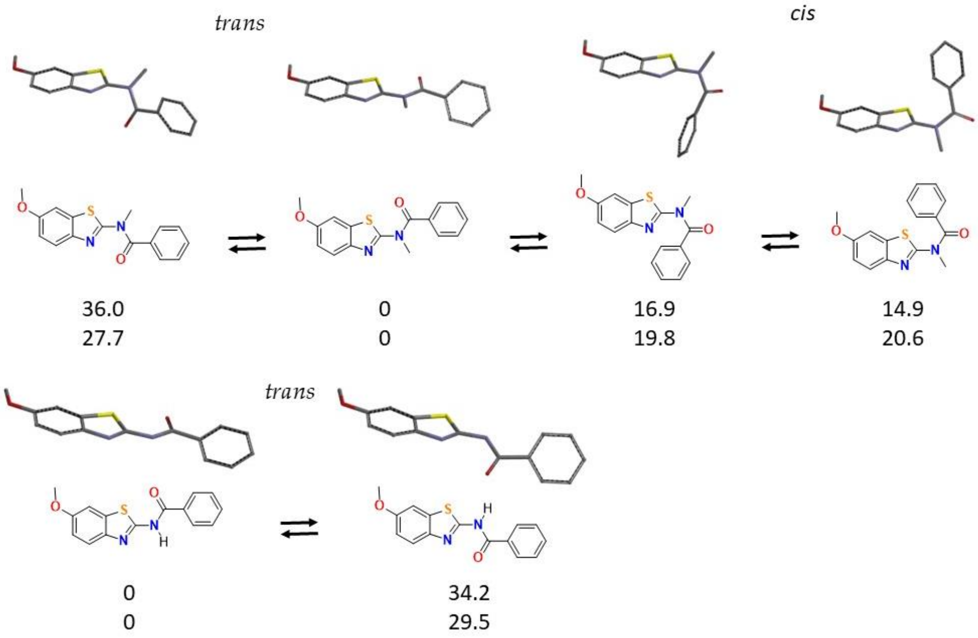

Figure S6. Configurations of the amide bonds in benzamides

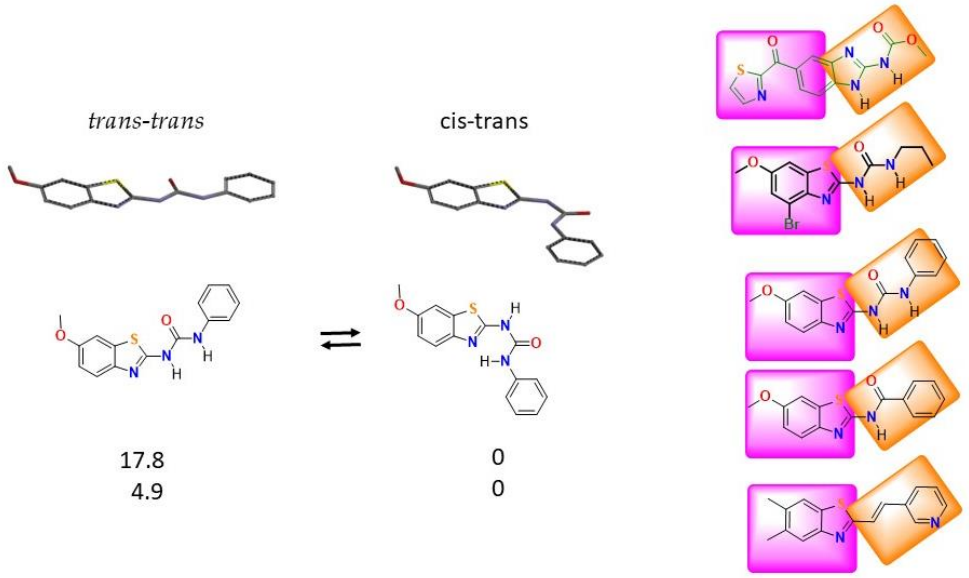

Figure S7. Configurations of the amide bonds in frentizole. The structural resemblance of frentizole to nocodazole is indicated by colored rectangles

Table S6. Docking results

Pose	PDB	PDBsite	SITE_OCC	Score	Complex_E	Receptor_E	Ligand_E	CombE	Z	E_PL	EPenRot	E/N	E/N1/3	E/PM	E/PM3root	E/cont	E_AD	Ki nM	Einterm	IW_Hbond_D	Eelctr	Eint	Etors	Eunb	Rank	AvE	ClSize
**MeOBZT_NHCONHPh_EE**																											
MeOBZT_NHCONHPh_EE_6BRY_60	6BRY	AB	BA	-26,5	-29339,8	29206,2	107,1	0,80	2,02								-8,8	346,3	-9,7	-9,7	0,0	-0,5	0,9	-0,5	2,0	-8,8	86
MeOBZT_NHCONHPh_EE_6PC4nw_conf_02	6PC4nw	BC	AB	-22,1	-5891,5	5758,0	111,4	0,79	1,64	-74,5	-68,5	-3,5	-27,0	-0,2	-11,1	-24,8											
MeOBZT_NHCONHPh_EE_7EMJww_conf_01	7EMJww	AC	CD	-24,5	-5662,2	5523,8	113,9	0,80	1,65	-74,6	-68,6	-3,6	-27,0	-0,2	-11,2	-24,9											
MeOBZT_NHCONHPh_EE_5S4Q_29	5S4Q	AC	CD	3,7	-28280,4	28173,3	110,8	0,78	1,88								-8,7	402,2	-9,6	-9,6	0,0	-0,6	0,9	-0,6	1,0	-8,7	77
**MeOBZT_NHCONHPh_EZ**																											
MeOBZT_NHCONHPh_EZ_6LSM_conf_04	6LSM	AB	BC	-35,9	-6008,4	5845,2	127,4	0,85	3,36	-88,8	-82,8	-4,2	-32,2	-0,3	-13,3	-30,0											
MeOBZT_NHCONHPh_EZ_5JVD_76	5JVD	AC	BC	-30,8	-6017,4	5845,2	141,4	0,76	1,43								-9,0	251,6	-9,9	-9,9	0,0	-1,0	0,9	-1,0	3,0	-8,99	29
**MeOBZT_NHCONHPh_ZE**																											
MeOBZT_NHCONHPh_ZE_5H7O_conf_01	5H7O	AB	BC	-34,4	-31552,1	31394,2	123,6	0,99	3,26	-91,2	-85,2	-4,3	-33,1	-0,3	-13,6	-30,9											
MeOBZT_NHCONHPh_ZE_8A9Z_88	8A9Z	BC	BC	-39,3	-30541,4	30378,3	123,9	0,67	1,74								-9,1	205,6	-10,0	-10,0	0,0	-0,3	0,9	-0,3	2,0	-9,1	79,0
**MeOBZT_NHCONHPh_ZZ**																											
MeOBZT_NHCONHPh_ZZ_5S4Q_92	5S4Q	AC	AC	-14,0	-28300,2	28173,3	112,9	0,90	1,97								-8,9	307,6	-9,8	-9,7	-0,1	-0,3	0,9	-0,3	2,0	-8,9	14
MeOBZT_NHCONHPh_ZZ_7EMJwn_conf_01	7EMJwn	AC	AC	-20,2	-8977,7	8833,1	124,5	0,88	3,05	-85,3	-79,3	-4,1	-30,9	-0,3	-12,8	-28,8											
**MeOBZTNH_NCONHPh_EE**					-31008,6	30841,8	126,7																				
MeOBZTNH_NCONHPh_EE_5YL2_82	5YL2	BC	BC	-40,1	-6008,8	5845,2	129,9	0,83	1,78								-8,7	440,6	-9,3	-9,3	0,0	-0,3	0,6	-0,3	1,0	-8,66	97
MeOBZTNH_NCONHPh_EE_5JVD_conf_01	5JVD	AC	BC	-33,7				0,80	2,76	-82,9	-76,9	-3,9	-30,1	-0,3	-12,4	-27,9											
**MeOBZTNH_NCONHPh_EZ**																											
MeOBZTNH_NCONHPh_EZ_5S4Q_conf_01	5S4Q	AC	AC	-15,0	-8984,8	8833,1	136,7	0,93	3,06	-86,01	-80,01	-4,10	-31,18	-0,29	-12,86	-29,00											
MeOBZTNH_NCONHPh_EZ_7Z2N_78	7Z2N	BC	CD	-19,8	1458662,6	-1458818,6	136,2	0,88	1,53								-8,72	405,57	-9,32	-9,31	-0,01	-0,24	0,60	-0,24	1,00	-8,72	99
**MeOBZT_NMeCO_Ph**																											
MeOBZT_NMeCO_Ph_5S4Q_4	5S4Q	AC	AC	7,9	-28173,3	28173,3	7,9	0,92	2,07								-8,6	462,4	-9,5	-9,5	0,0	-0,4	0,9	-0,4	1,0	-8,6	85
MeOBZT_NMeCO_Ph_7EMJnw_conf_01	7EMJnw	AC	AC	-27,7	3135,7	-3175,4	12,0	0,75	2,65	-77,4	-71,4	-3,7	-28,0	-0,3	-11,6	-25,9											
**MeOBZT_NMeCOZ_Ph**																											
MeOBZT_NMeCOZ_Ph_5CB4_conf_02	5CB4	AB	AB	-38,9	-31072,5	31015,8	17,9	0,83	1,84	-74,05	-68,05	-3,53	-26,84	-0,25	-11,08	-24,67											
MeOBZT_NMeCOZ_Ph_5XKG_79	5XKG	AB	AB	-36,5	-30429,0	30378,3	14,2	0,82	5,17								-8,45	643,97	-9,34	-9,33	-0,01	-0,79	0,89	-0,79	1	-8,37	82
**MeOBZT_NHCONHEt_EE**																											
MeOBZT_NHCONHEt_EE_3N2K_38	3N2K	AC	CD					0,77	2,05								-7,0	7,2	-7,9	-8,0	0,0	-0,3	0,9	-0,3	1,0	-6,84	26
MeOBZT_NHCONHEt_EE_5S4Q_conf_02	5S4Q	AC	CD					0,69	1,37	-66,8	-60,8	-3,9	-26,0	-0,3	-10,6	-23,6											
**MeOBZT4Br_NHCONHEt_EE**																											
MeOBZT4Br_NHCONHEt_EE_5S4Q_conf_01	5S4Q	AC	AC	-28,2	-21475,8	21349,0	98,6	0,76	2,33	-72,31	-66,31	-4,02	-27,59	-0,22	-10,46	-25,30											
MeOBZT4Br_NHCONHEt_EE_6FKL_76	6FKL	BC	CD	-13,7	-21468,0	21349,0	105,3	0,73	1,72								-7,56	2,87	-8,46	-8,35	-0,11	-0,22	0,89	-0,22	1	-7,42	23
**MeOBZT4Br_NHCONHEt_ZE**																											
MeOBZT4Br_NHCONHEt_ZE_6D88_35	6D88	BC	B	-28,4	-28534,2	28391,7	114,1	1,00	2,84								-8,22	935,65	-9,12	-9,15	0,03	-0,20	0,89	-0,20	1,00	-8,16	81
MeOBZT4Br_NHCONHEt_ZE_6D88_conf_01	6D88	BC	B	-37,8	245940,0	-246099,3	121,5	0,92	3,30	-78,39	-72,39	-4,36	-29,91	-0,24	-11,34	-27,62											
**MeOBZT4Br_NHCONHEt_ZZ**																											
MeOBZT4Br_NHCONHEt_ZZ_6O5N_77	6O5N	AB	B	-20,6	-5978,7	5845,2	113,0	1,01	2,88								-8,24	918,55	-9,13	-9,16	0,03	-0,15	0,89	-0,15	1	-8,22	82
MeOBZT4Br_NHCONHEt_ZZ_5XLZ_conf_01	5XLZ	BC	B	-8,3	-31121,5	30997,5	115,7	1,00	3,76	-81,40	-75,40	-4,52	-31,06	-0,25	-11,78	-28,77											
**MeBZT4Br_NHCONHEt_EE**																											
MeBZT4Br_NHCONHEt_EE_5LYJ_CA4_100	CA4	BC	B					1,01	3,24								-8,62	479,51	-9,22	-9,23	0,02	-0,11	0,60	-0,11	1,00	-8,6	67
MeBZT4Br_NHCONHEt_EE_6O5Mnww_conf_01	6O5Mnww	BC	B					0,88	2,84	-75,82	-71,82	-4,46	-29,49	-0,24	-11,15	-27,93											
**MeBZT4Br_NHCONHEt_EZ**																											
MeBZT4Br_NHCONHEt_EZ_6O5N_conf_01	6O5N	AB	B					1,00	3,76	-83,97	-79,97	-4,94	-32,66	-0,27	-12,35	-31,10											
MeBZT4Br_NHCONHEt_EZ_5JVD_7	5JVD	AC	B					0,95	2,25								-8,45	642,42	-9,04	-9,00	-0,04	-0,19	0,60	-0,19	1	-8,37	36
**MeBZT4Br_NHCONHEt_ZE**																											
MeBZT4Br_NHCONHEt_ZE_5H7O_48	5H7O	AB	B					0,99	2,96								-8,58	513,65	-9,18	-9,17	-0,01	-0,16	0,60	-0,16	1	-8,51	34
MeBZT4Br_NHCONHEt_ZE_8C0F_conf_01	8C0F	BC	B					0,84	2,86	-77,55	-73,55	-4,56	-30,16	-0,25	-11,41	-28,60											
**MeBZT4Br_NHCONHEt_ZZ**																											
MeBZT4Br_NHCONHEt_ZZ_8C0F_conf_01	8C0F	BC	B					0,97	3,60	-80,57	-76,57	-4,74	-31,34	-0,26	-11,85	-29,78											
MeBZT4Br_NHCONHEt_ZZ_5H7O_30	5H7O	AB	B					0,92	2,16								-8,28	858,64	-8,87	-8,89	0,02	-0,14	0,60	-0,14	2,00	-8,26	8

•

Figure S8. ^{1}H- and ^{13}C-NMR spectra for the synthesized compounds

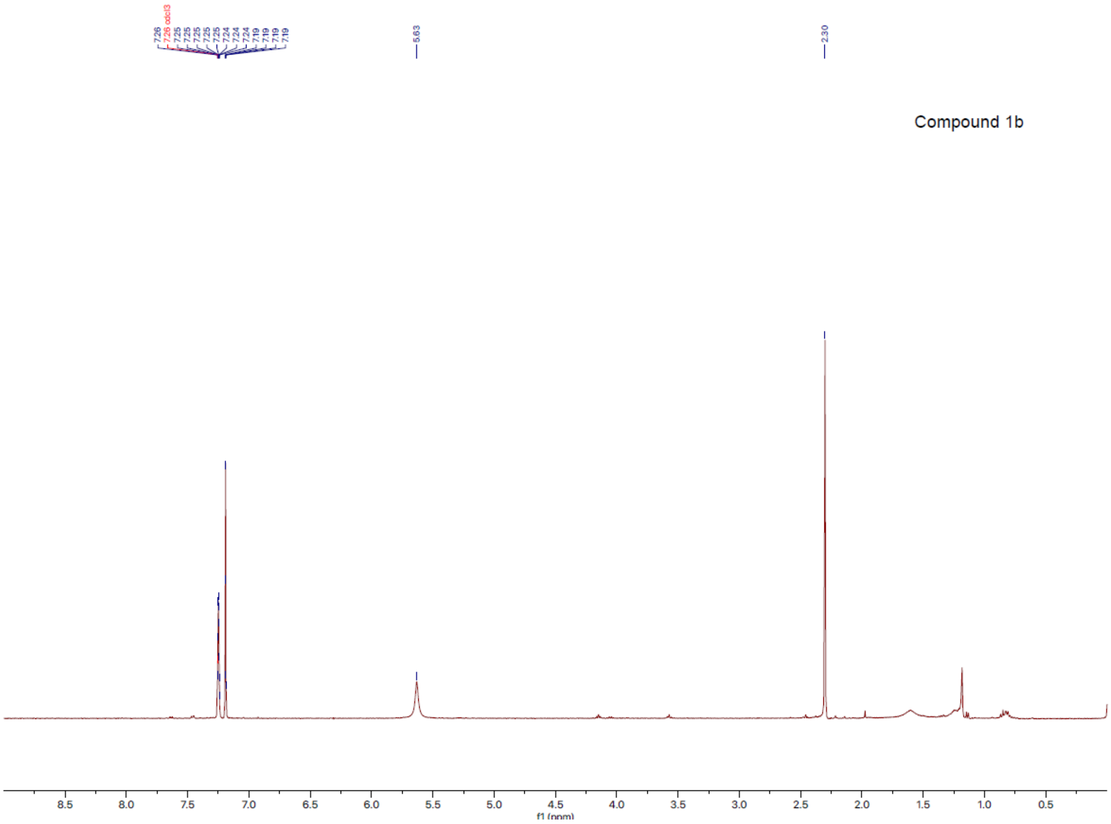

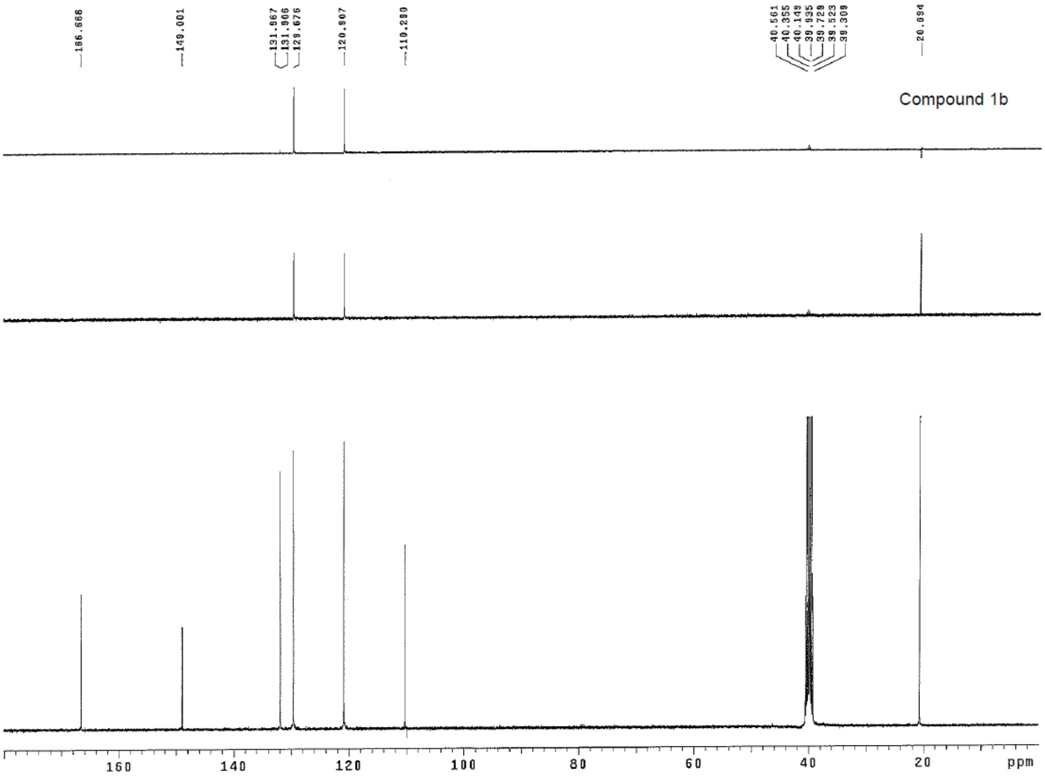

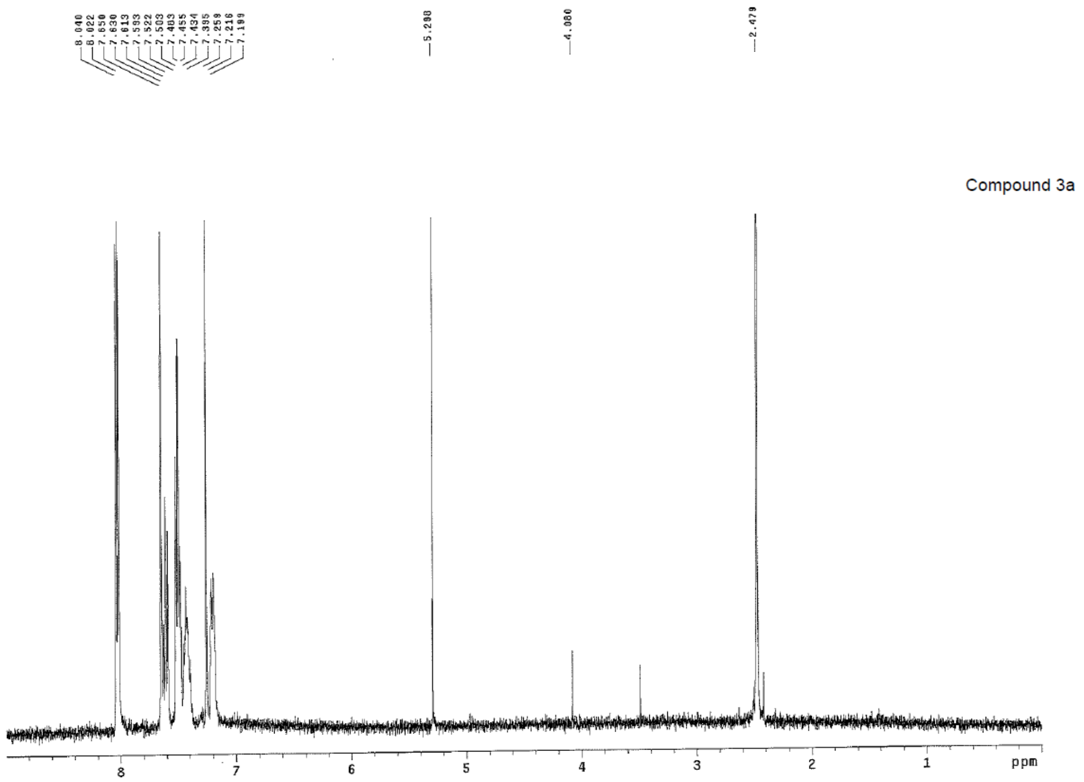
Compound 3a
ppm

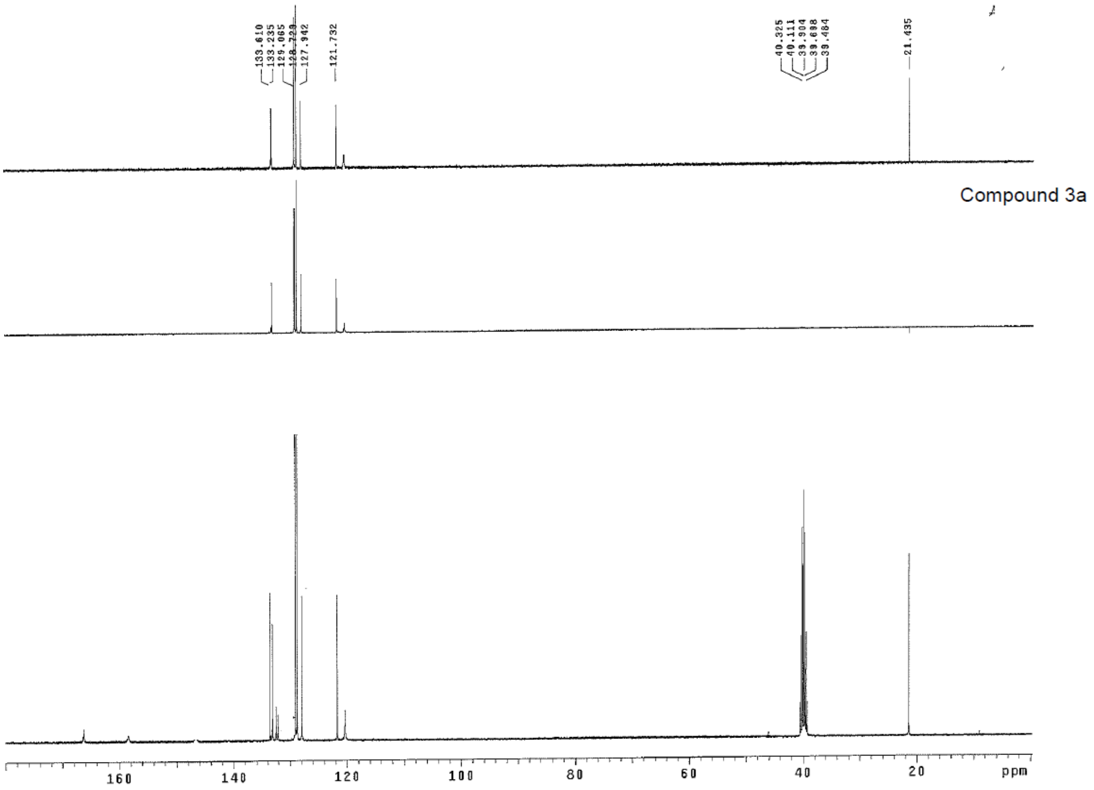
Compound 3a
ppm

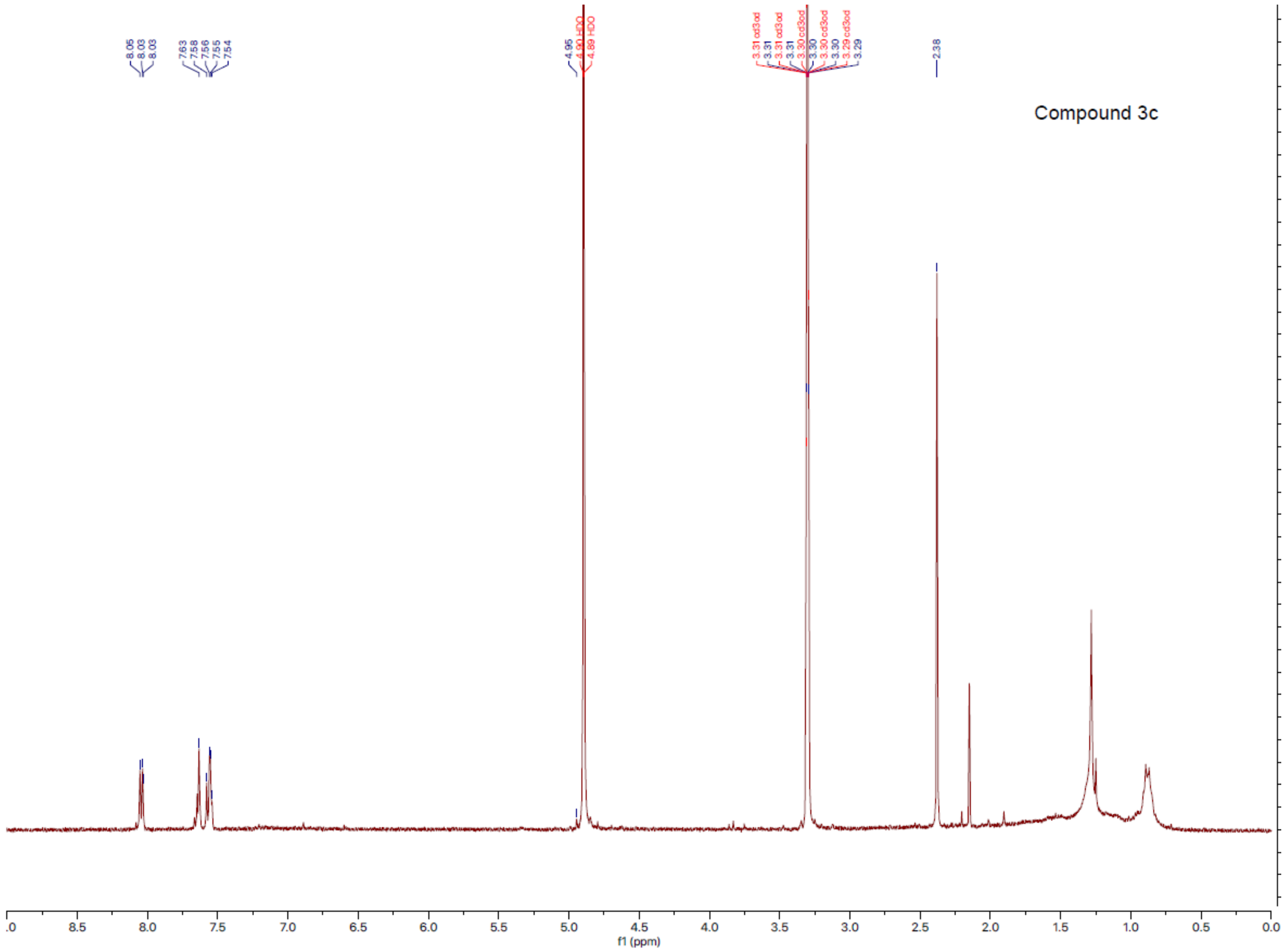
Compound 3c
f1 (ppm)

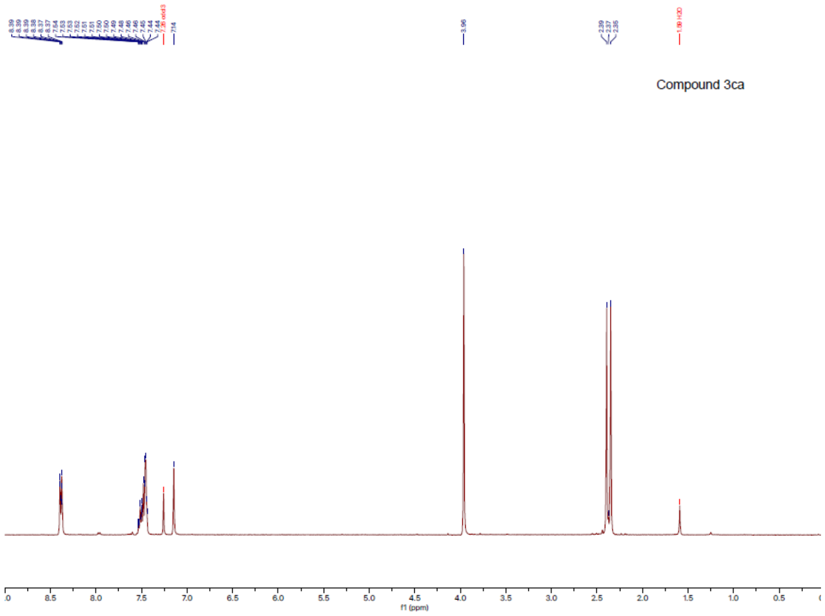
Compound 3ca
f1 (ppm)

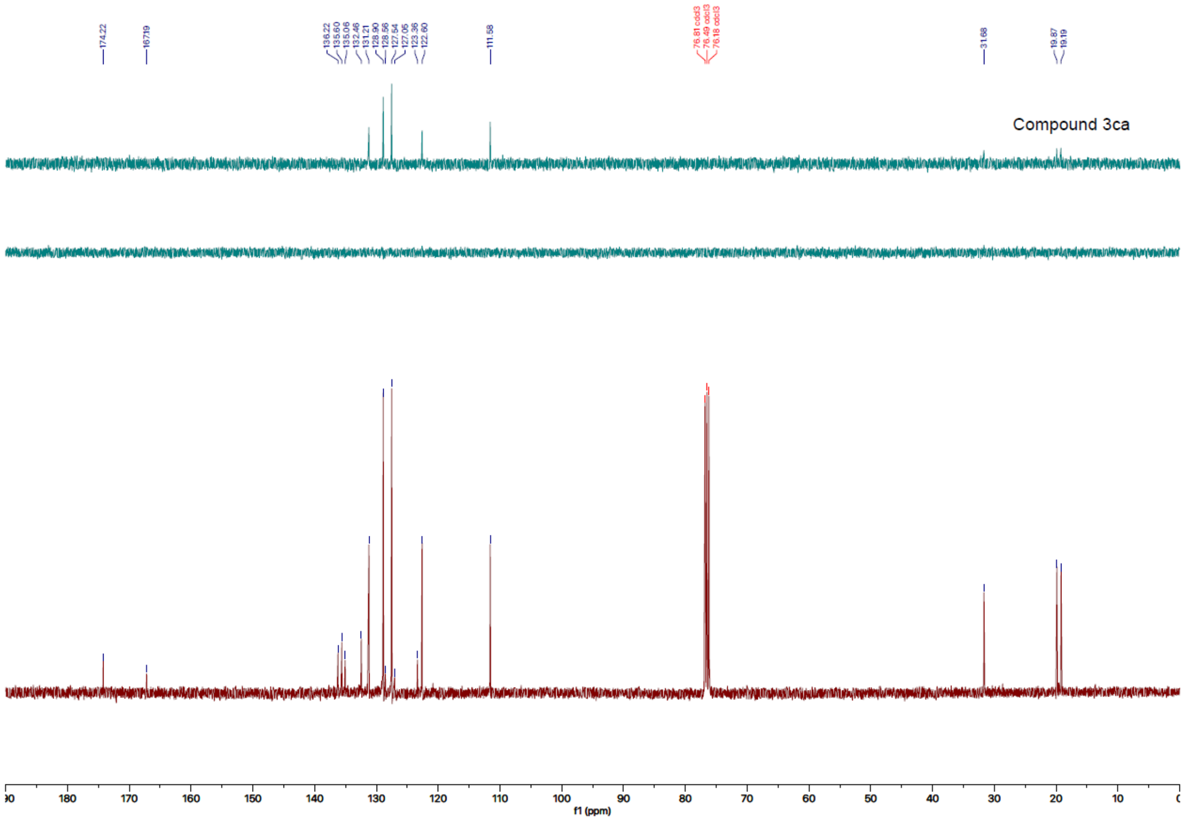
Compound 3ca
f1 (ppm)

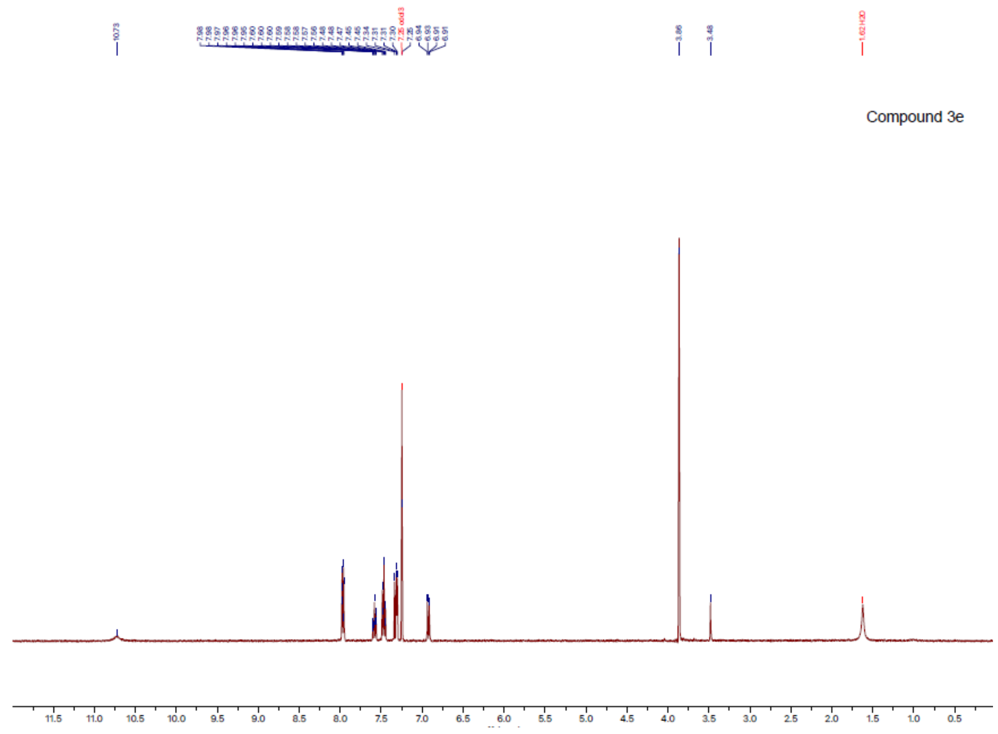
10.73
3.86
3.48
1.62 H2O
Compound 3e

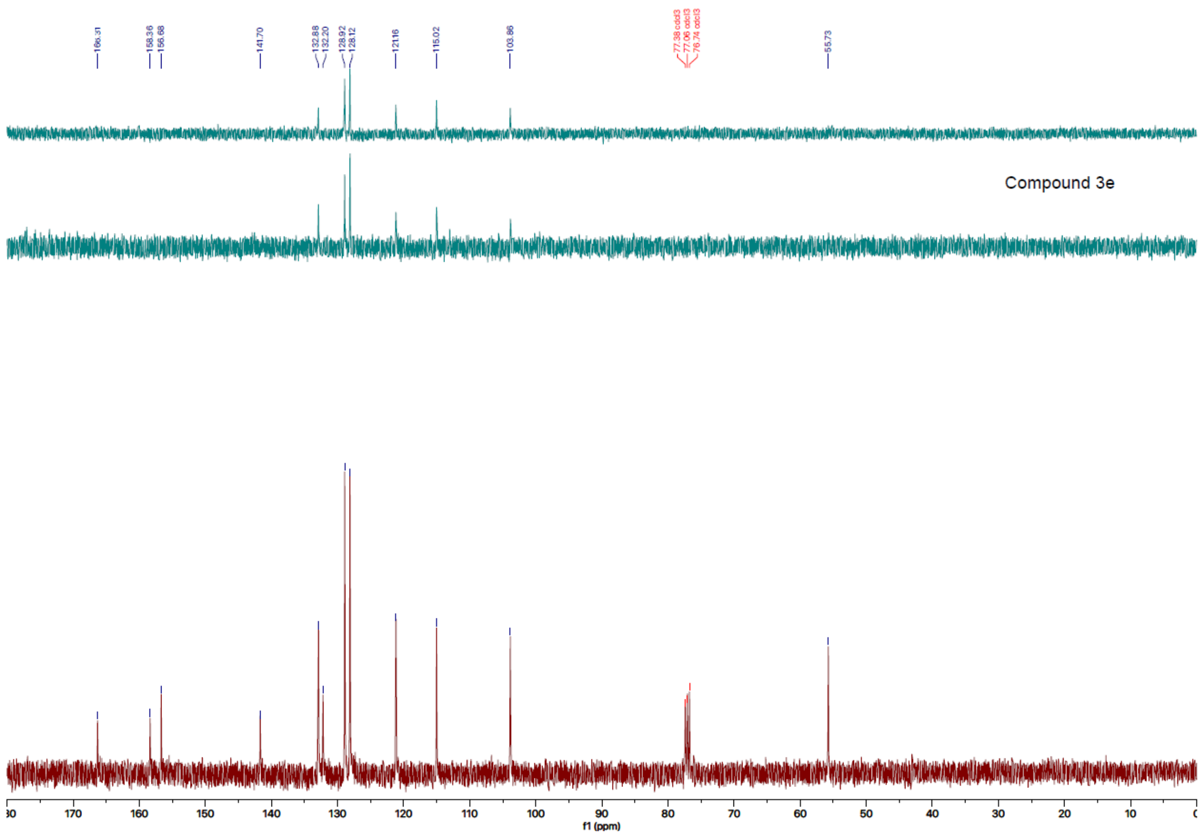
166.31
158.36
156.68
141.70
132.88
132.20
128.92
128.12
121.16
115.02
103.86
77.38 cdcl3
77.06 cdcl3
76.74 cdcl3
55.73
Compound 3e
f1 (ppm)

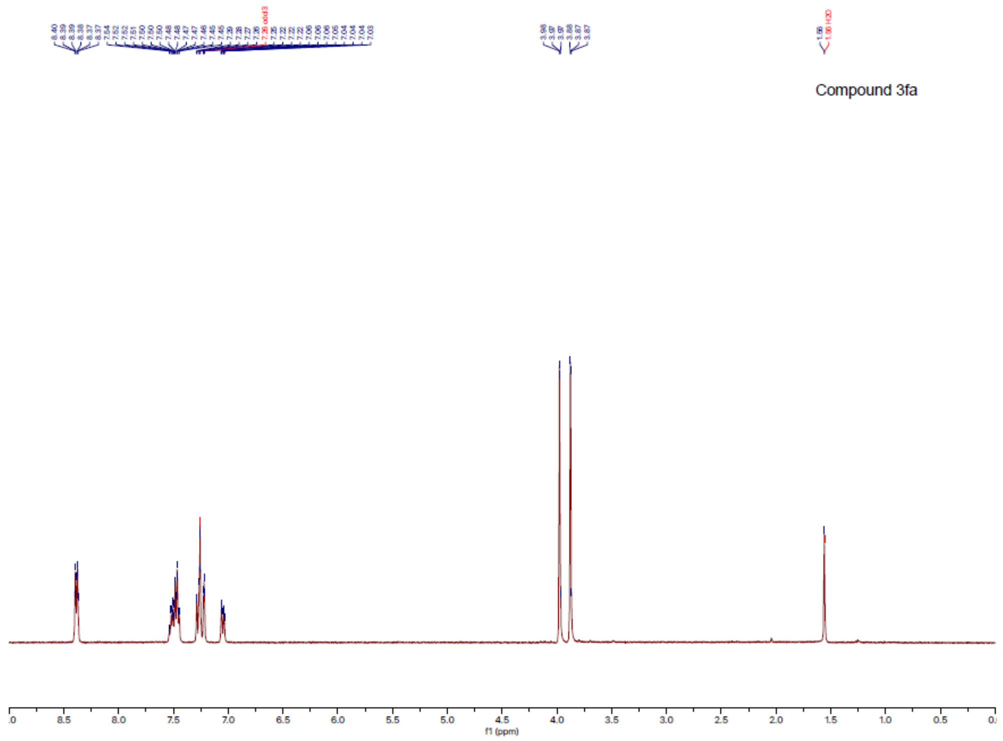
Compound 3fa
f1 (ppm)

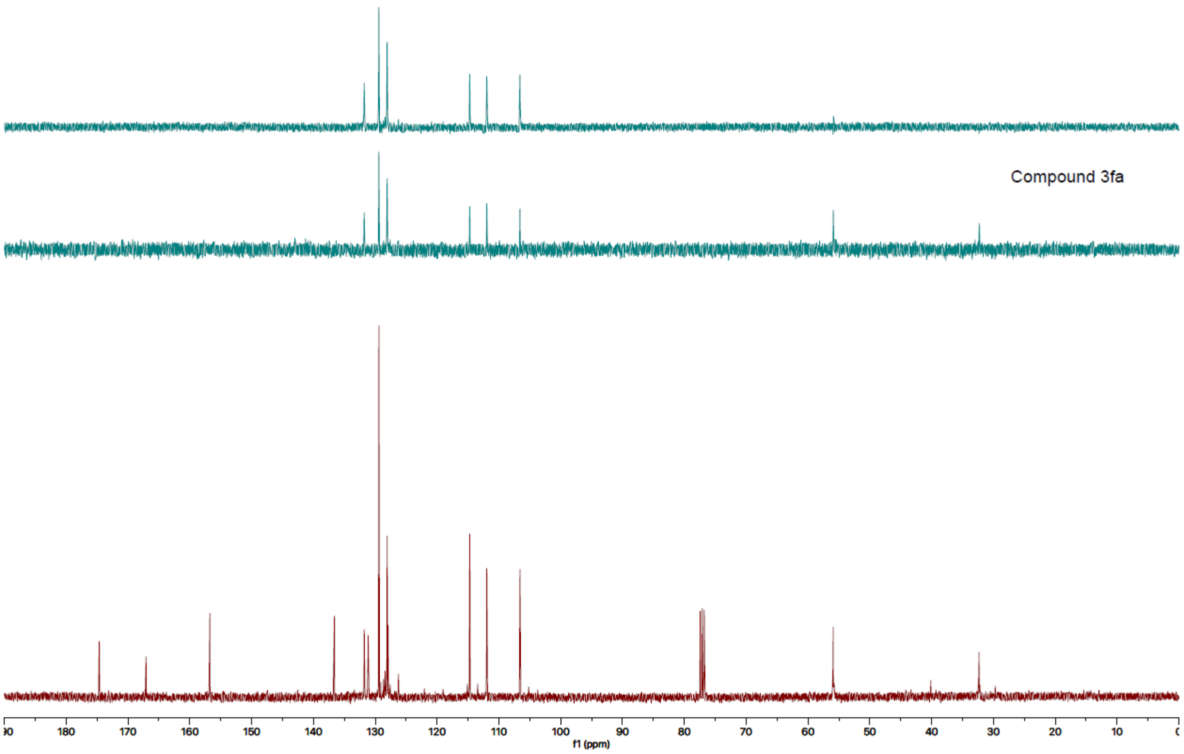
Compound 3fa
f1 (ppm)

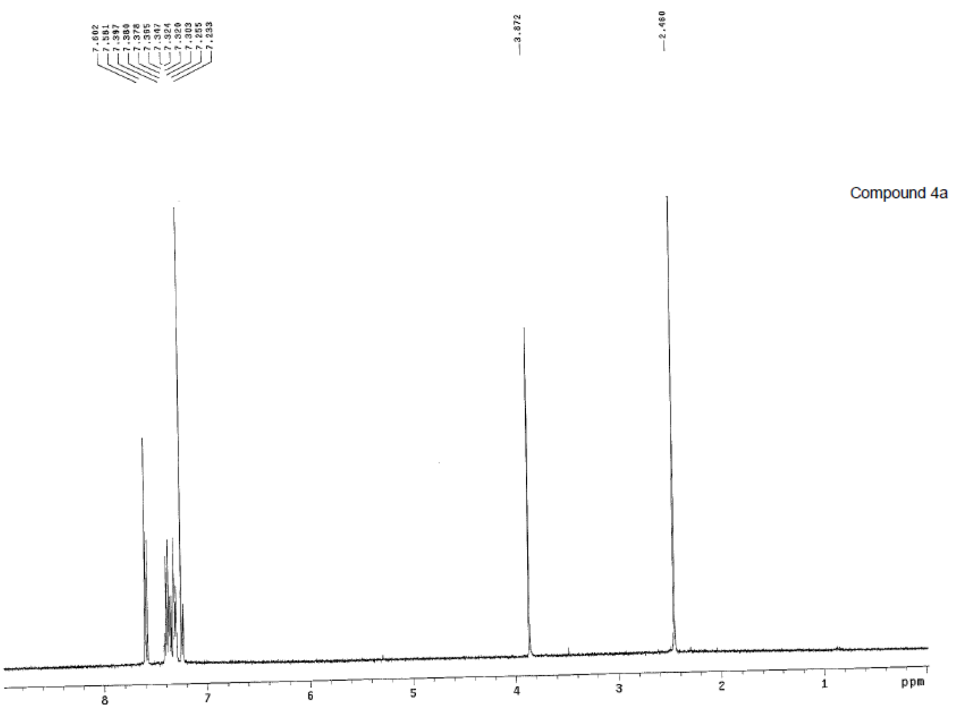
3.872
2.460
Compound 4a
8
7
6
5
4
3
2
1
ppm

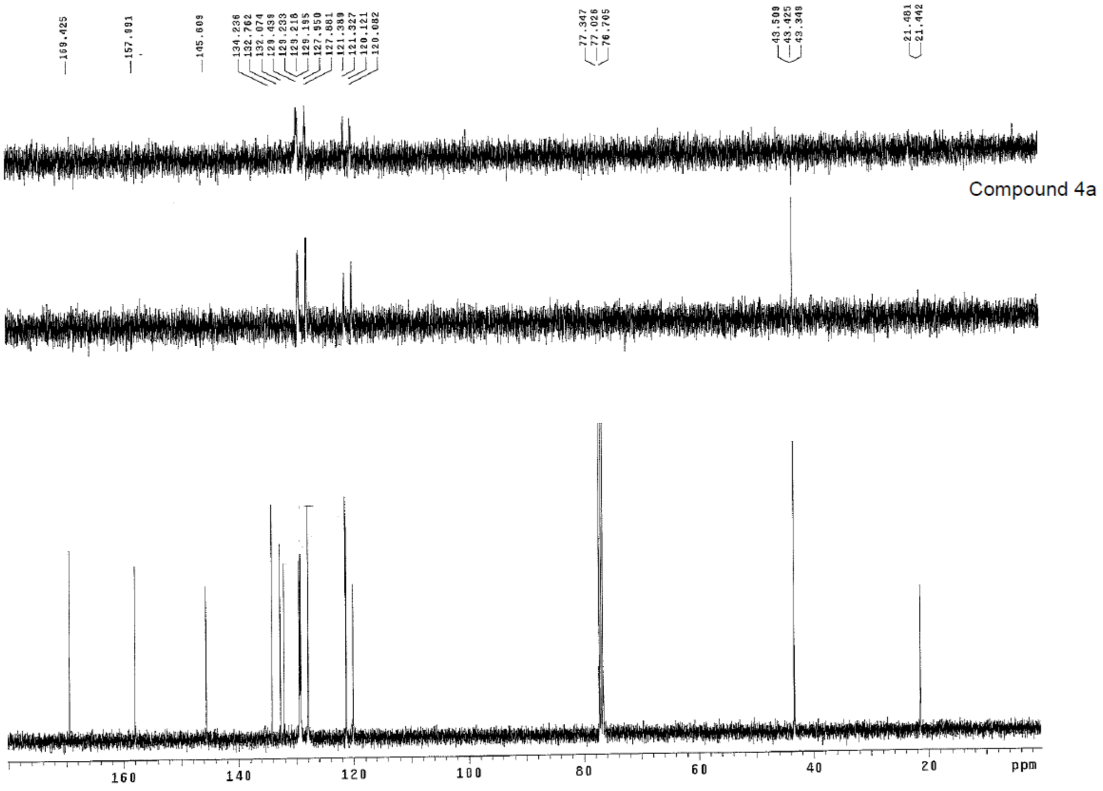
77.347
77.026
76.706
43.509
43.425
43.340
21.481
21.442
Compound 4a
160
140
120
100
80
60
40
20
ppm

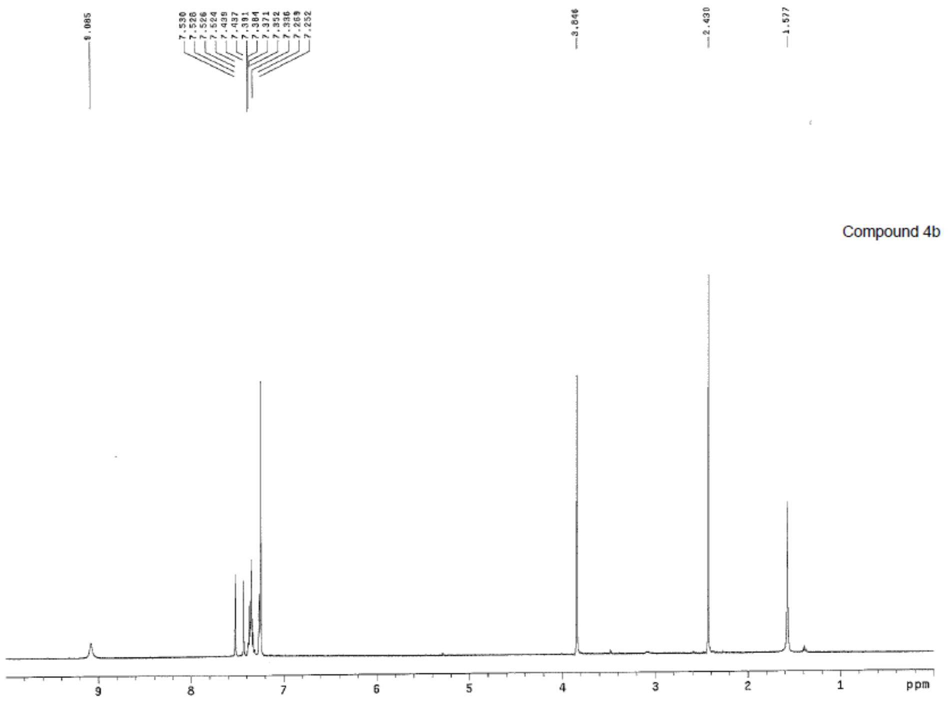
9.085
7.530
7.528
7.526
7.524
7.439
7.437
7.391
7.384
7.371
7.352
7.336
7.269
7.252
3.846
2.430
1.577
Compound 4b
9
8
7
6
5
4
3
2
1
ppm

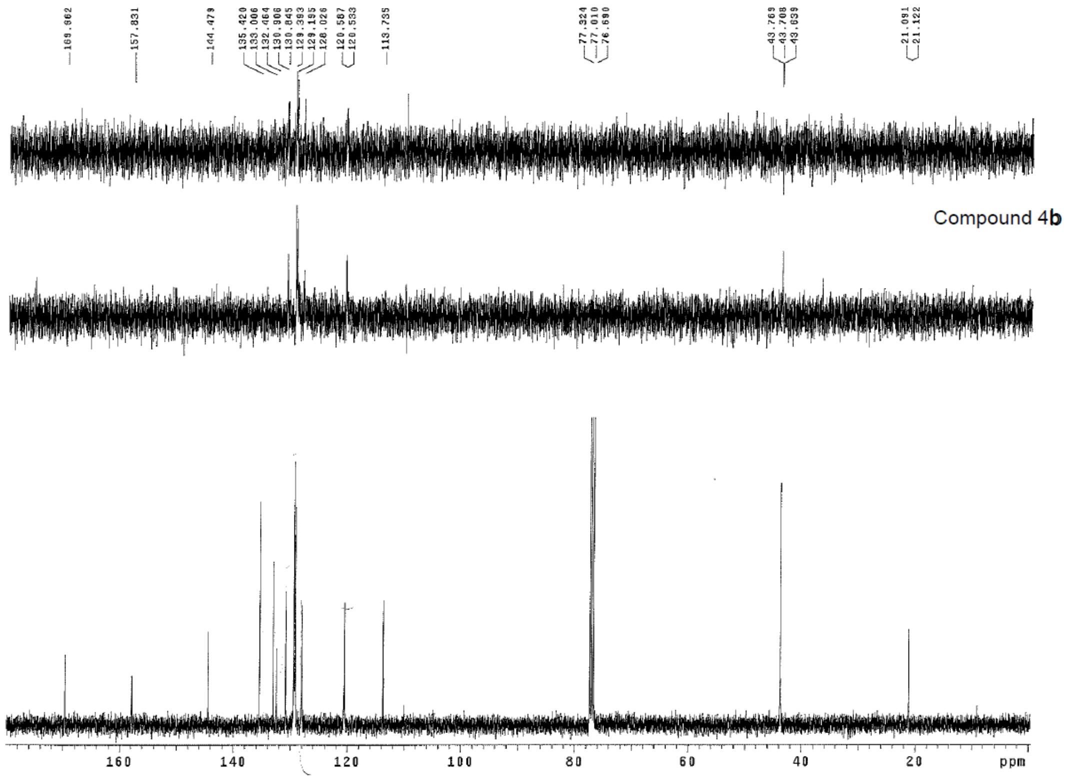
169.862
157.831
144.479
135.420
133.006
132.464
130.906
130.845
129.393
129.185
128.026
120.587
120.533
113.735
77.324
77.010
76.690
43.769
43.708
43.639
21.091
21.122
Compound 4b
160
140
120
100
80
60
40
20
ppm

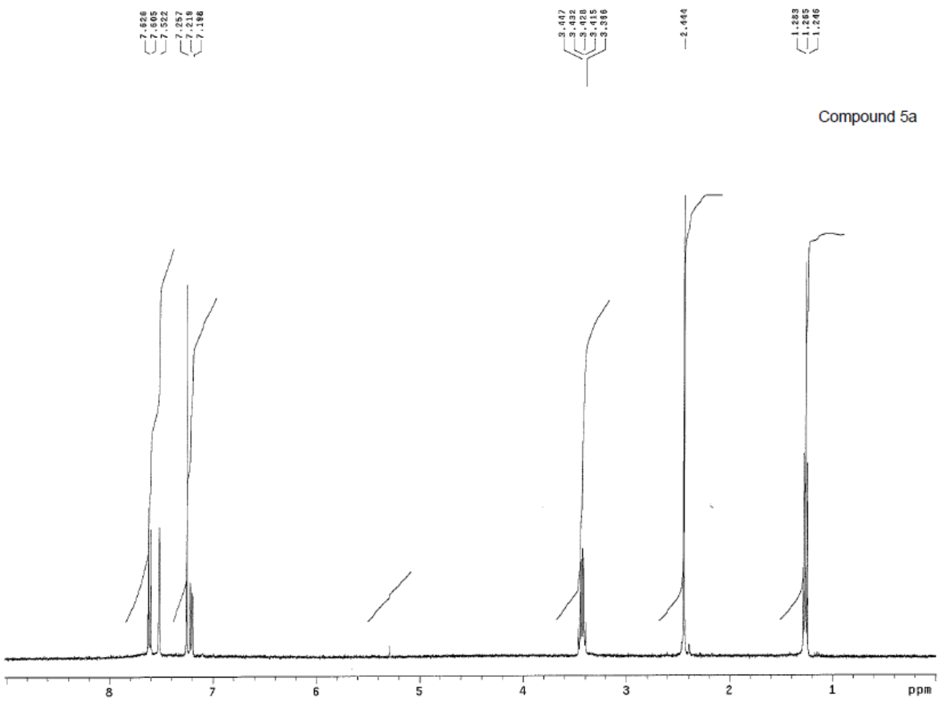
7.626
7.605
7.522
7.257
7.218
7.198
3.442
3.432
3.428
3.415
3.396
2.444
1.283
1.265
1.246
Compound 5a
8
7
6
5
4
3
2
1
ppm

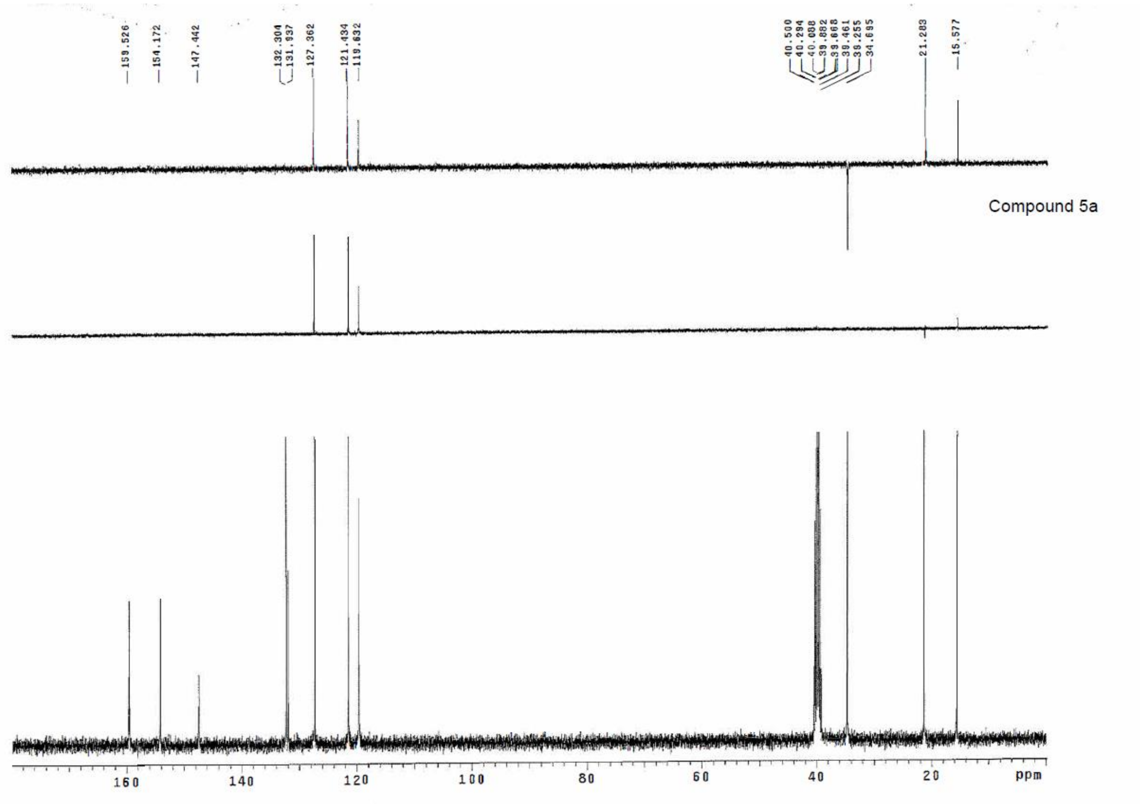
159.526
154.172
147.442
132.304
131.937
127.362
121.434
119.632
40.500
40.294
40.088
39.882
39.668
39.461
39.255
34.695
21.283
15.577
Compound 5a
160
140
120
100
80
60
40
20
ppm

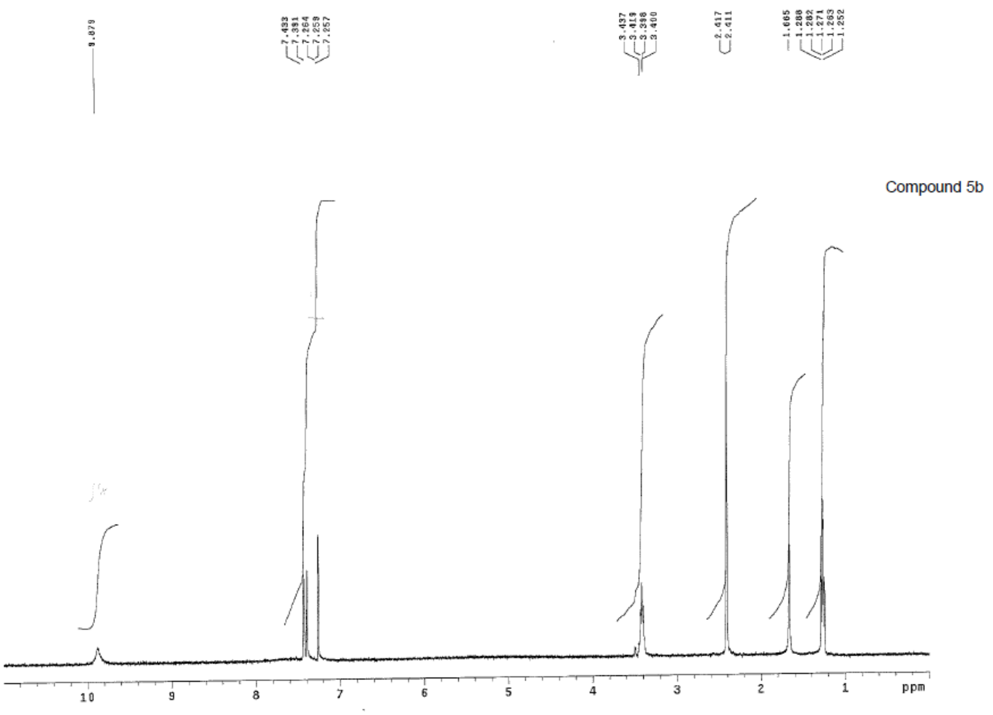
Compound 5b
10
9
8
7
6
5
4
3
2
1
ppm

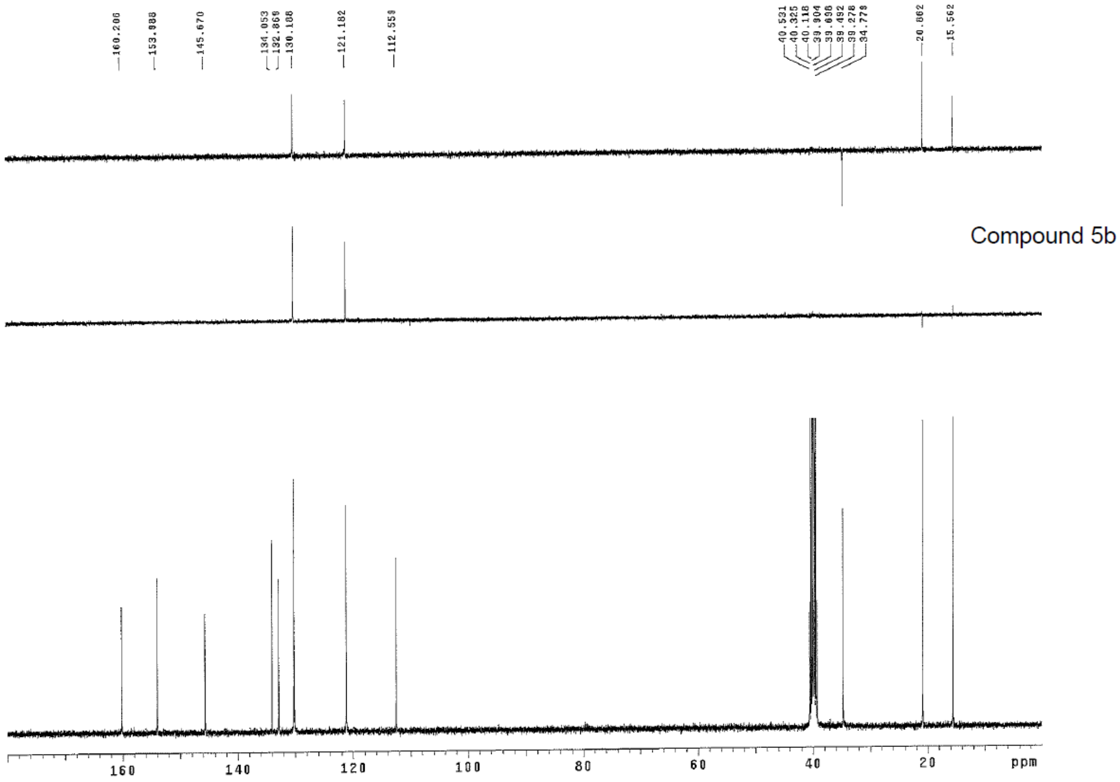
Compound 5b
160
140
120
100
80
60
40
20
ppm

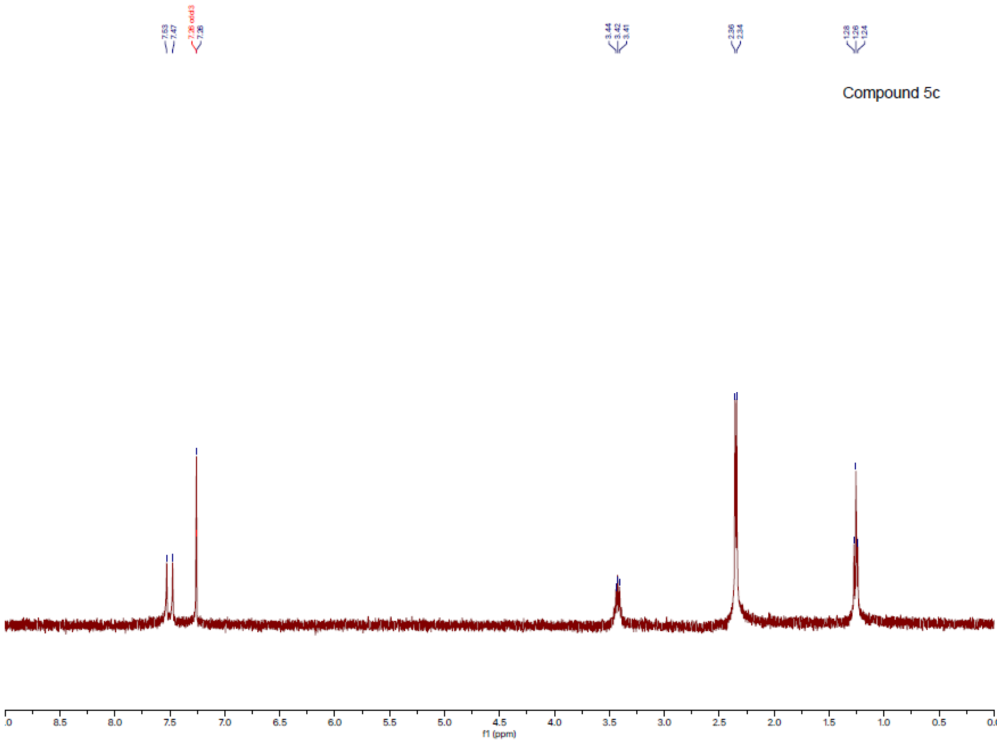
Compound 5c
f1 (ppm)

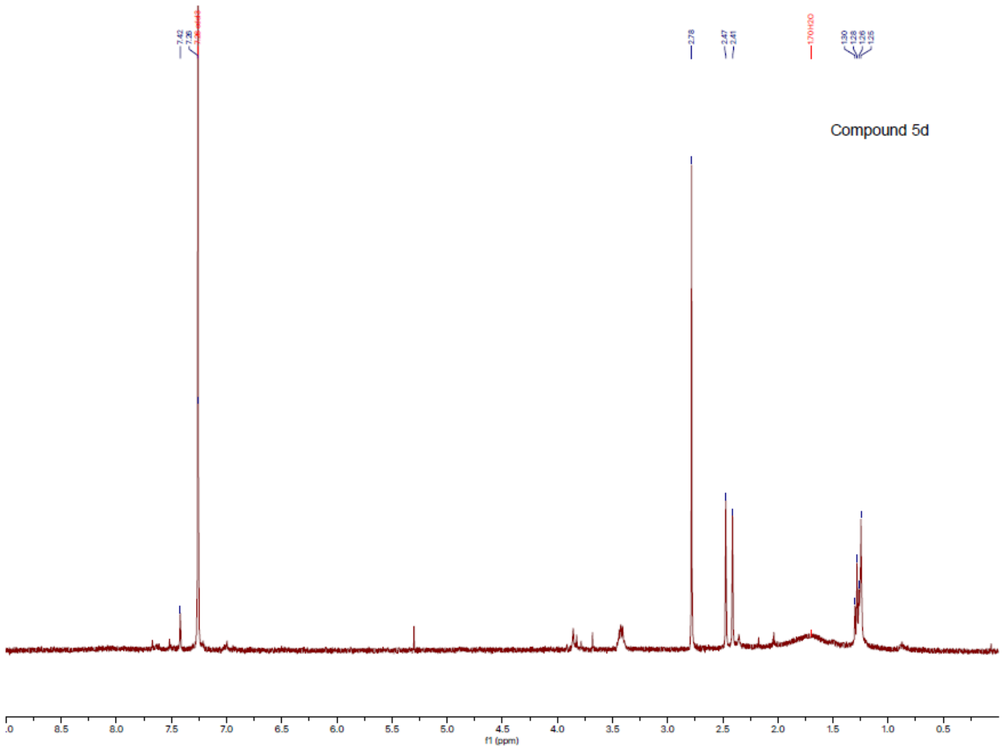
Compound 5d
f1 (ppm)

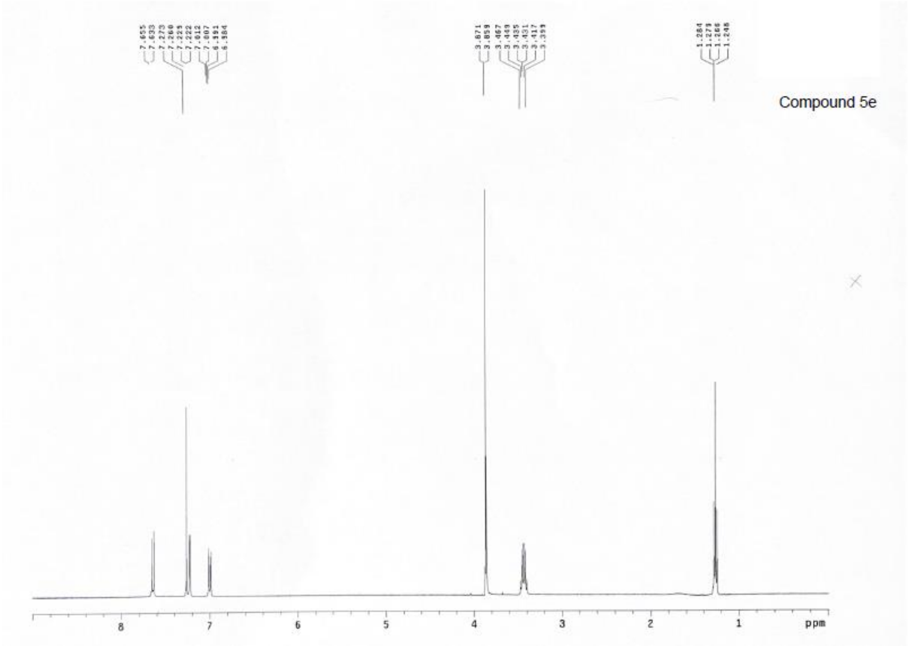
Compound 5e
ppm

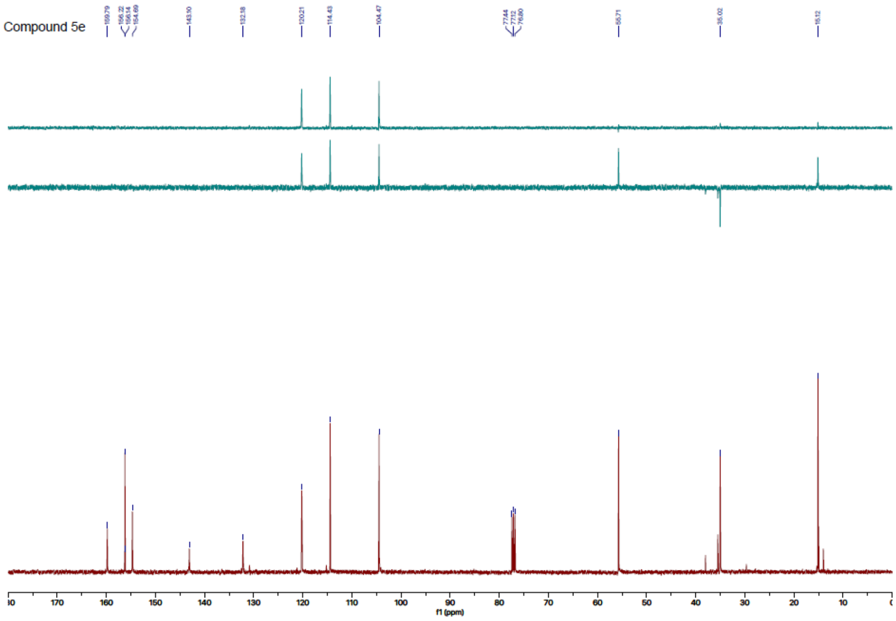
Compound 5e
f1 (ppm)

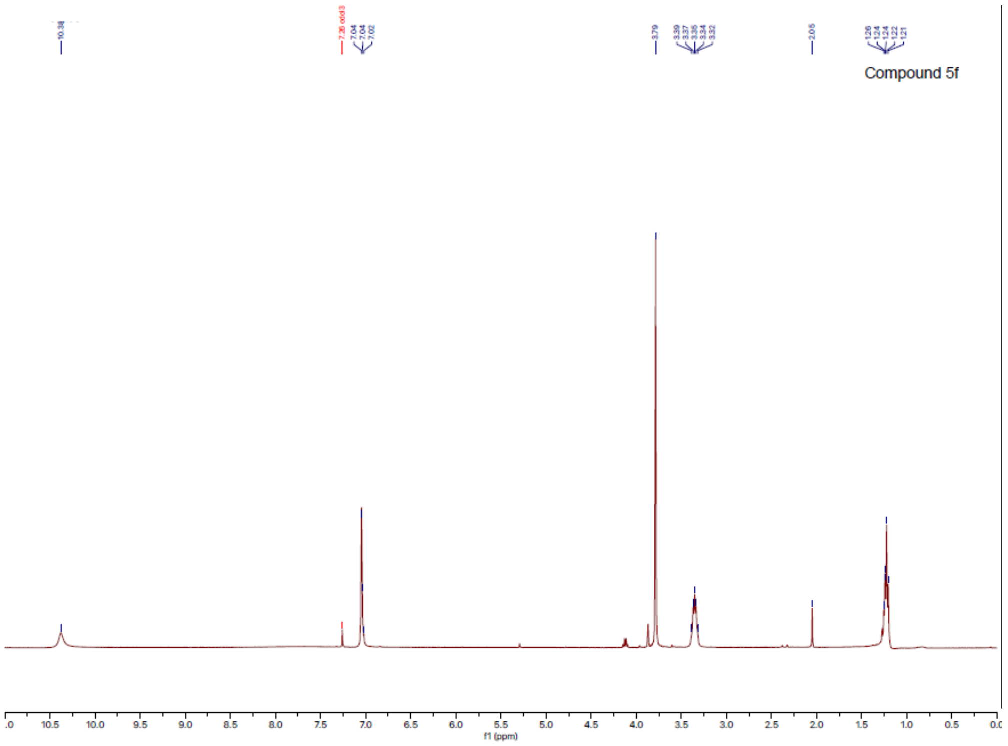
Compound 5f

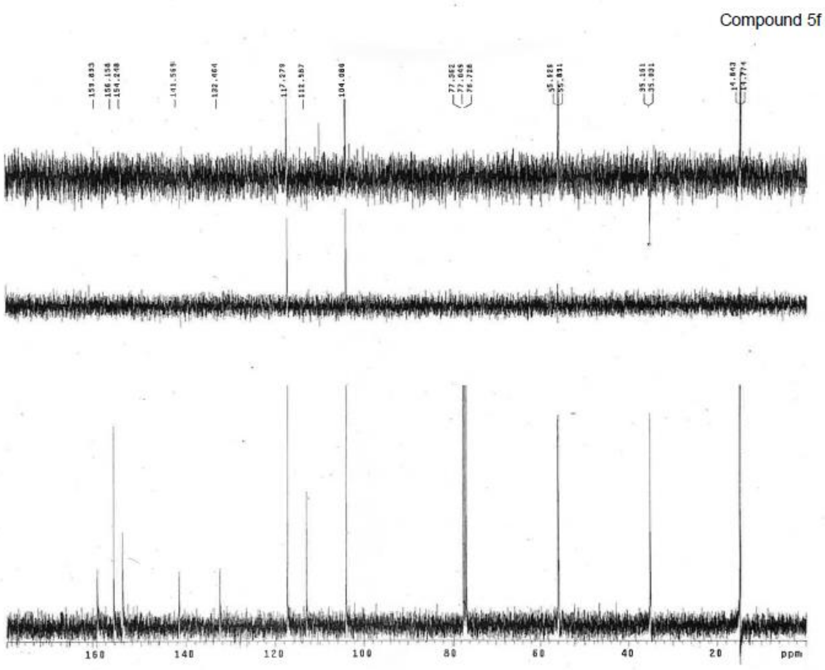
Compound 5f

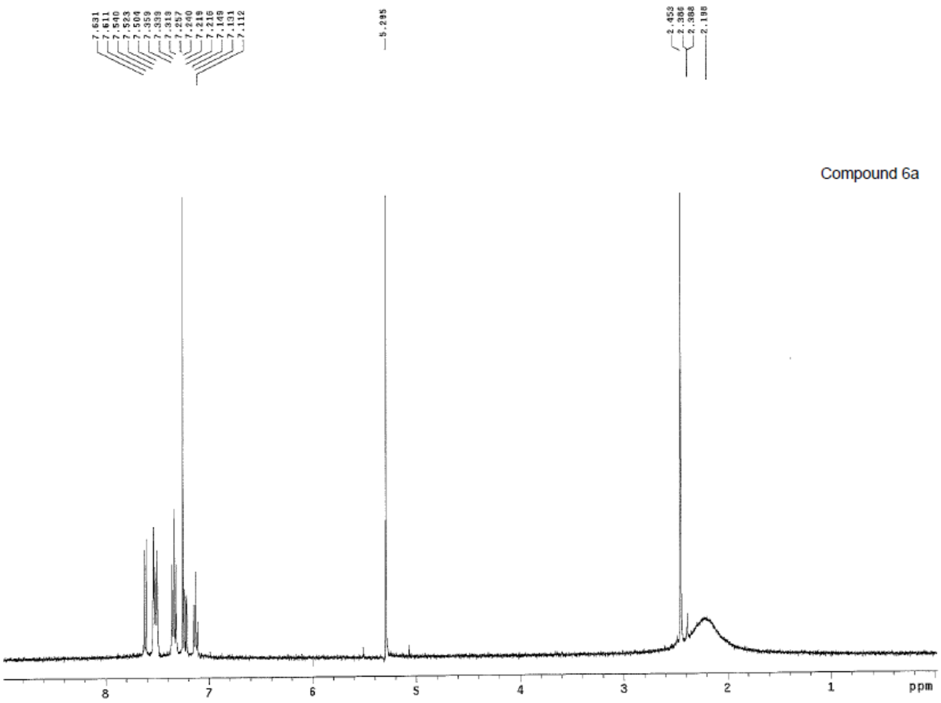
Compound 6a
ppm

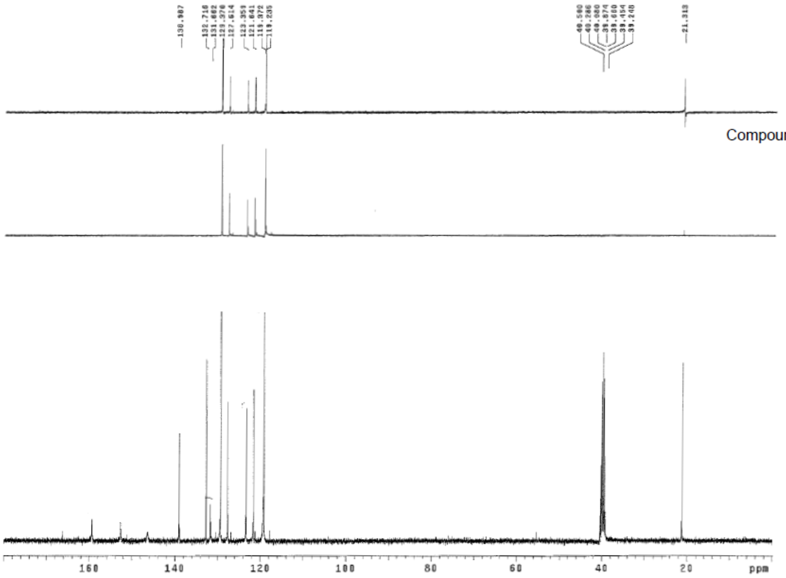
Compound 6a
ppm

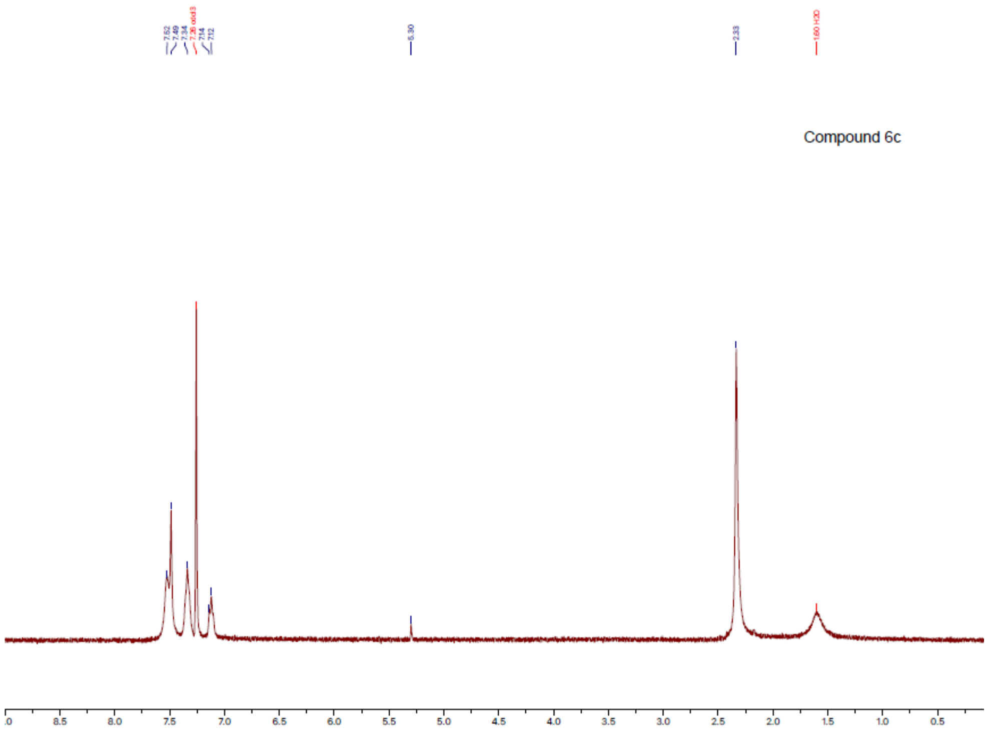
7.52
7.49
7.34
7.26 cdcl3
7.14
7.12
5.30
2.33
1.60 H2O
Compound 6c
8.5 8.0 7.5 7.0 6.5 6.0 5.5 5.0 4.5 4.0 3.5 3.0 2.5 2.0 1.5 1.0 0.5

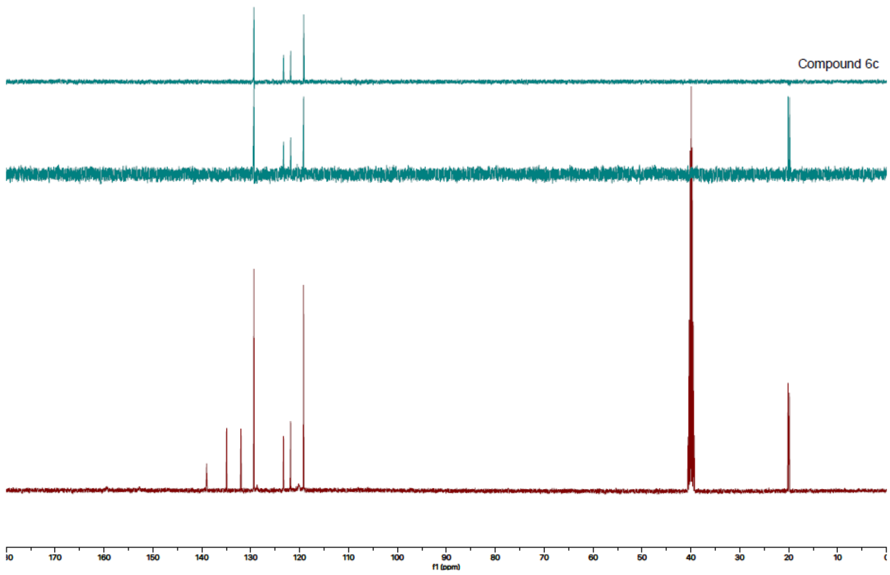
Compound 6c
170 160 150 140 130 120 110 100 90 80 70 60 50 40 30 20 10
f1 (ppm)

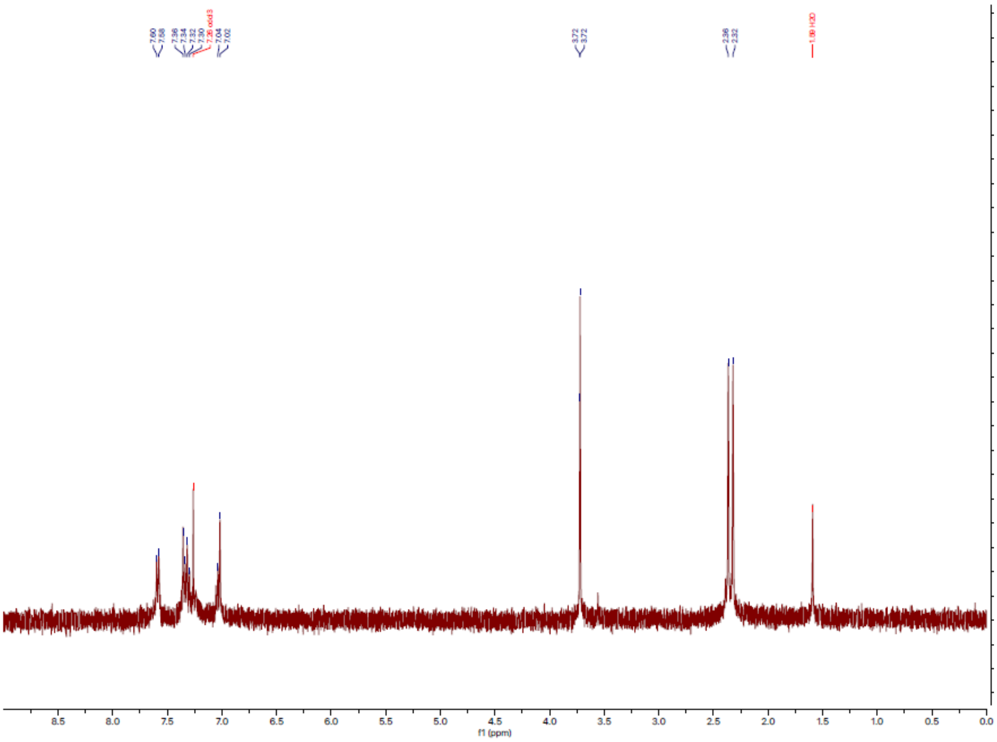
f1 (ppm)

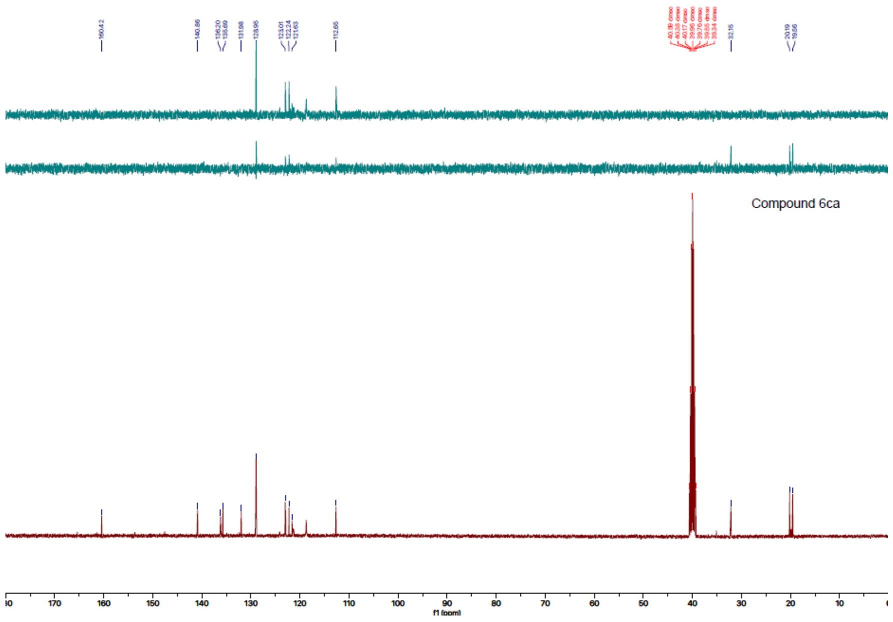
Compound 6ca
f1 (ppm)

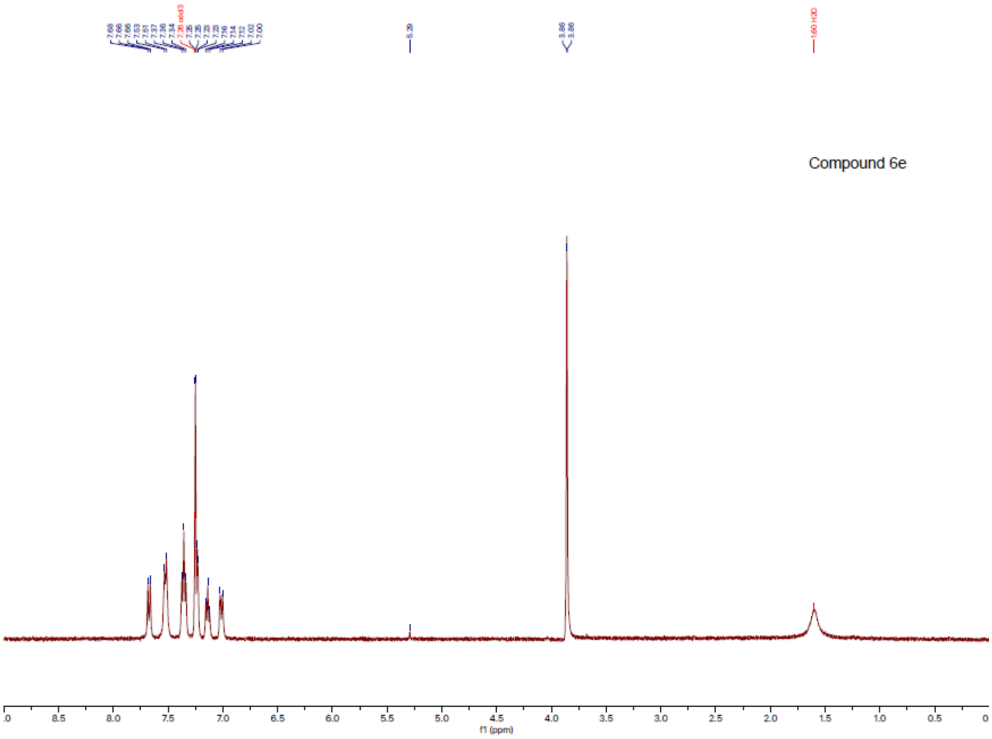
Compound 6e
f1 (ppm)

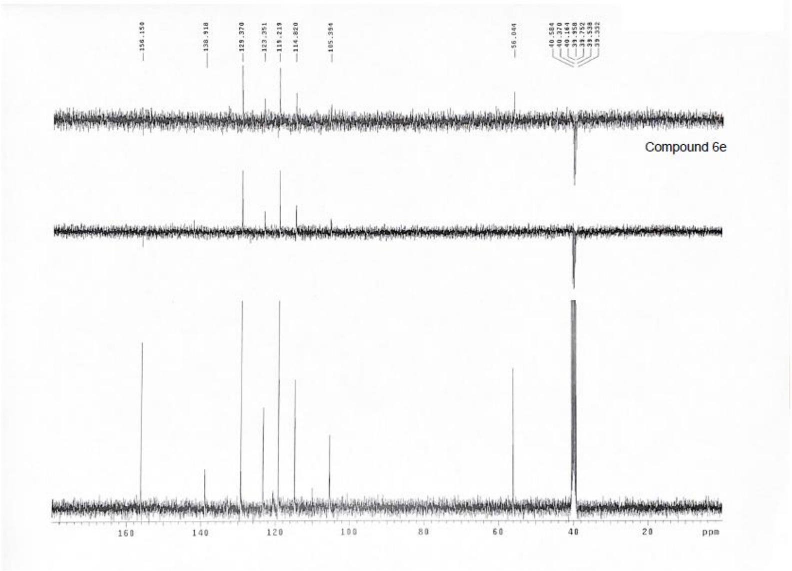
Compound 6e
ppm

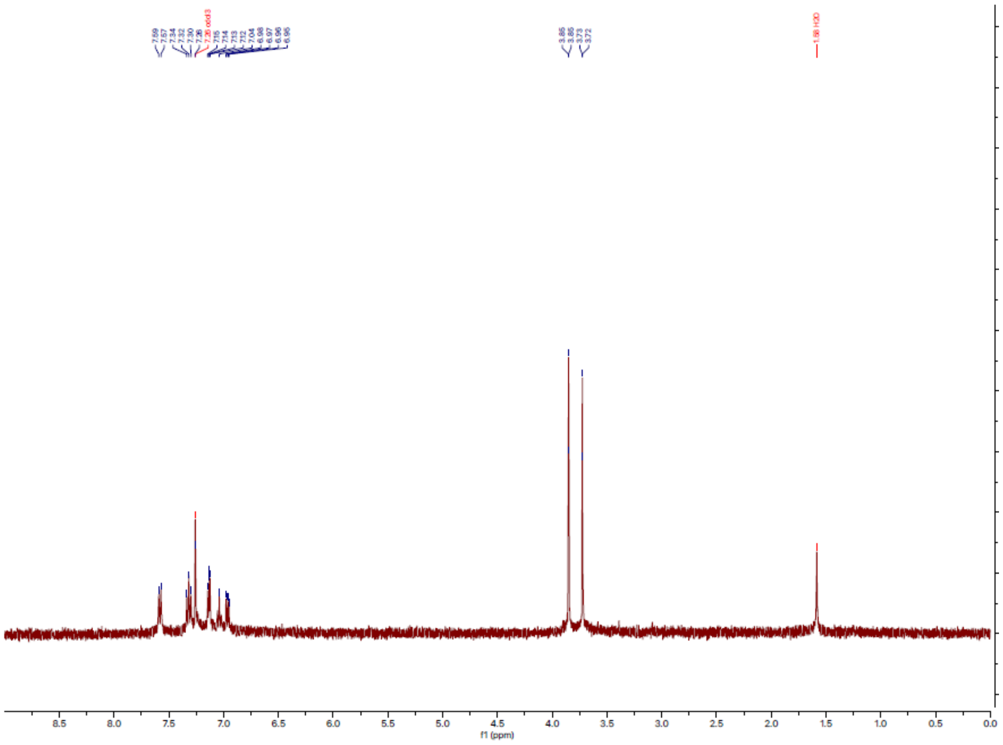
f1 (ppm)
1.58 H2O

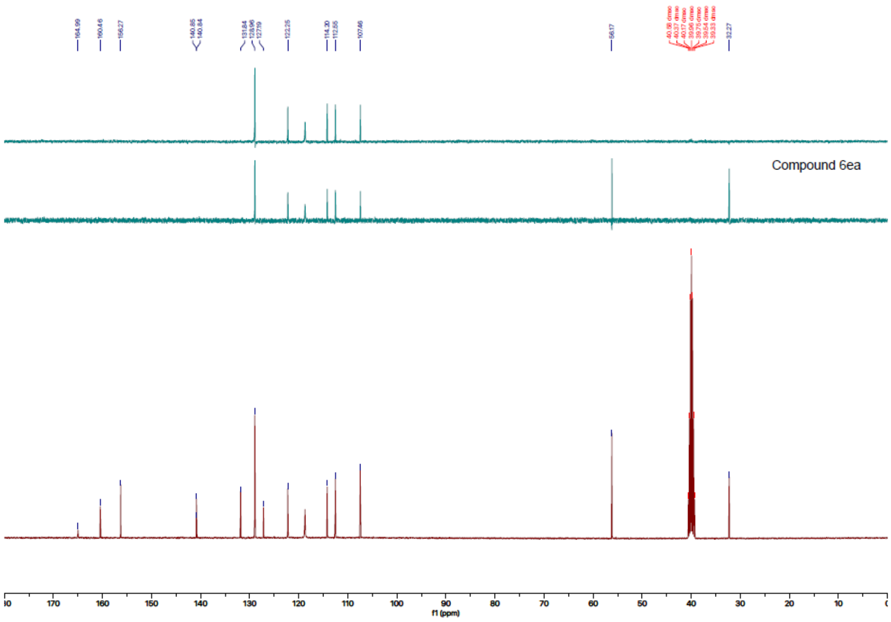
Compound 6ea
f1 (ppm)

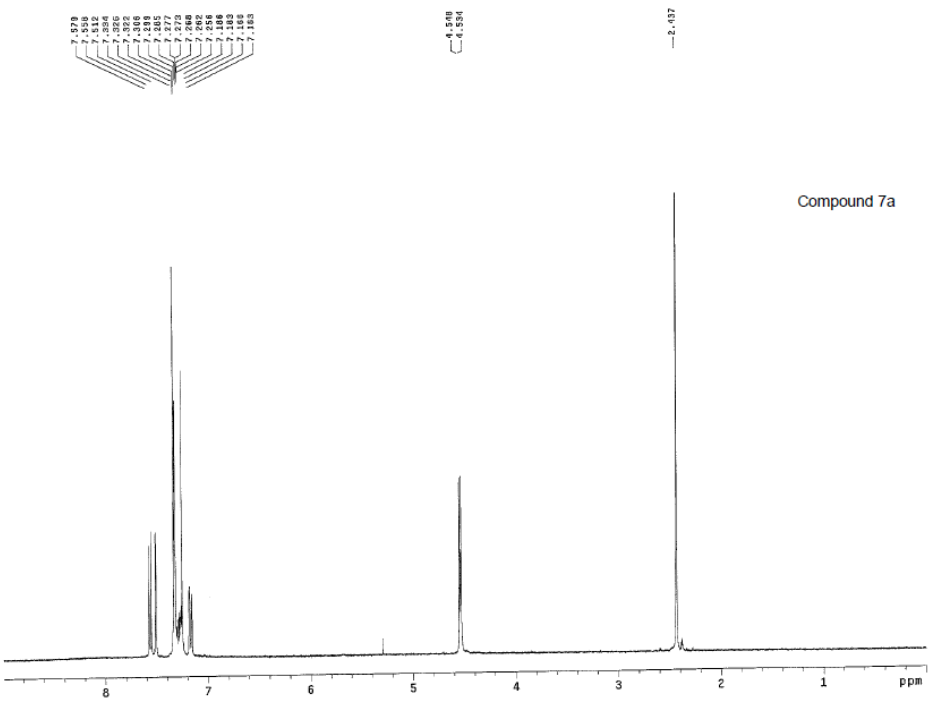
Compound 7a
8
7
6
5
4
3
2
1
ppm

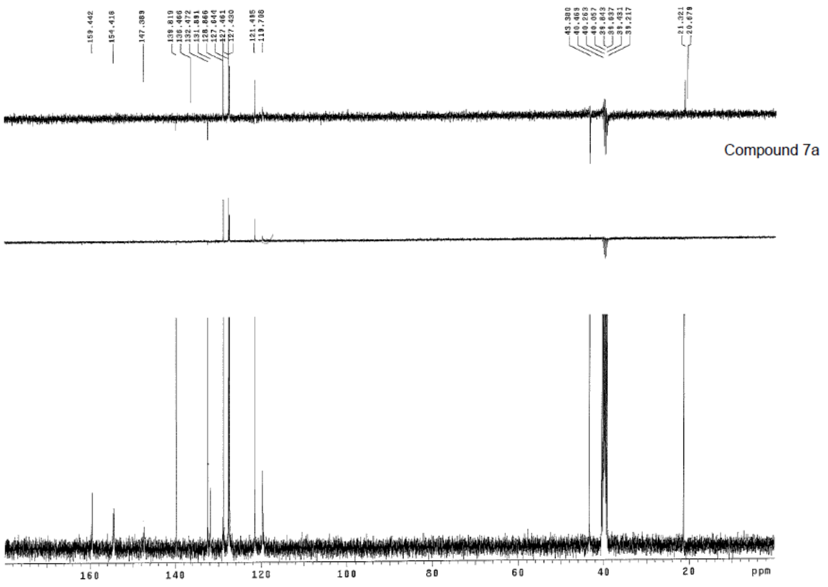
Compound 7a
160
140
120
100
80
60
40
20
ppm

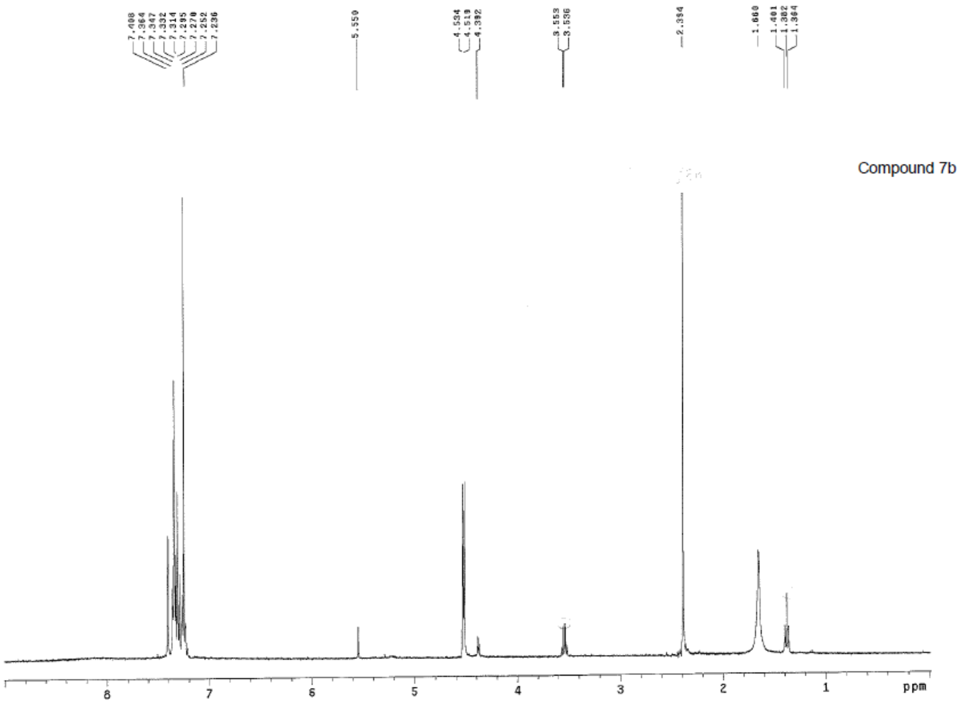
Compound 7b
ppm

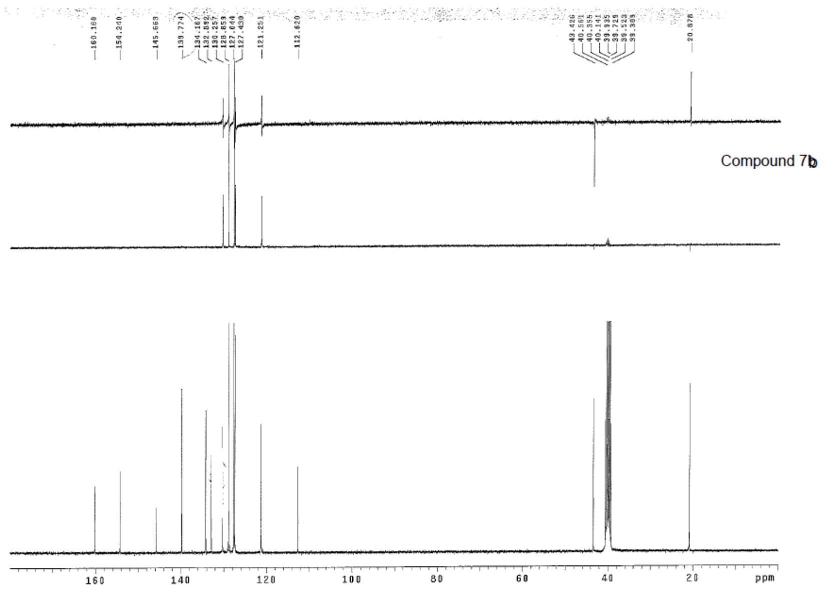
Compound 7b
ppm

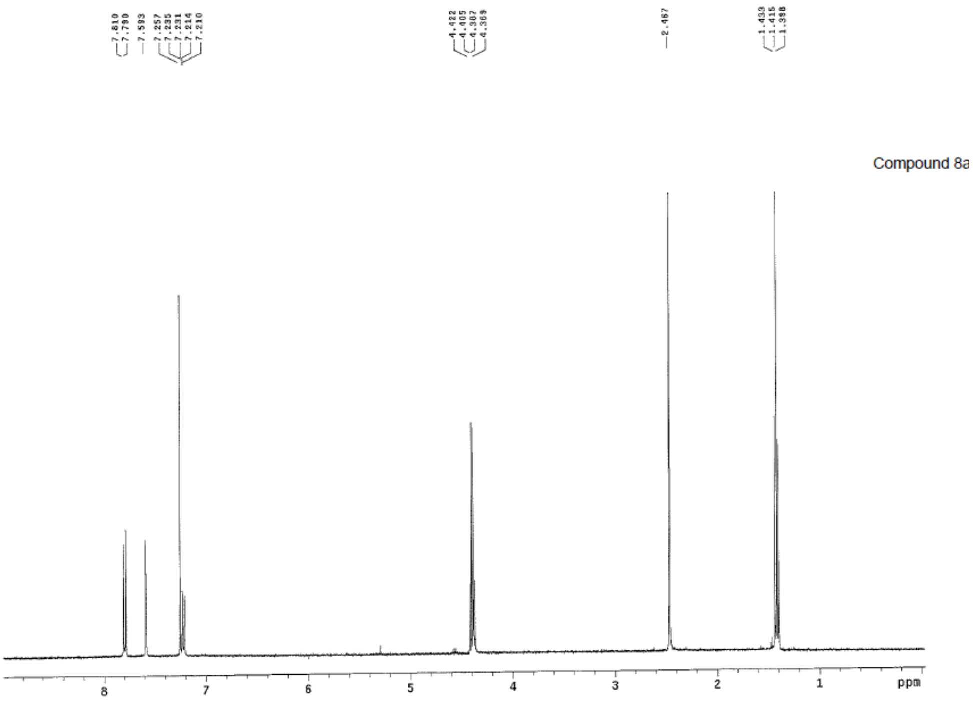
Compound 8a

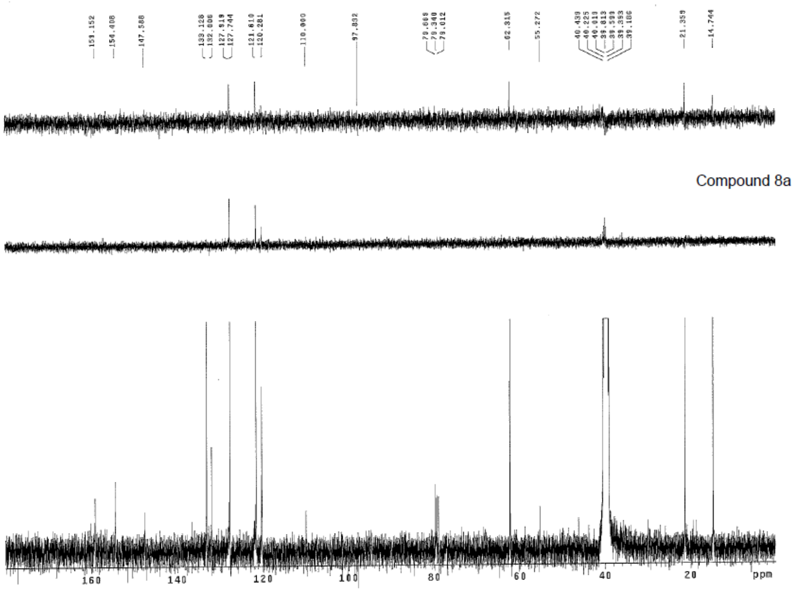
Compound 8a

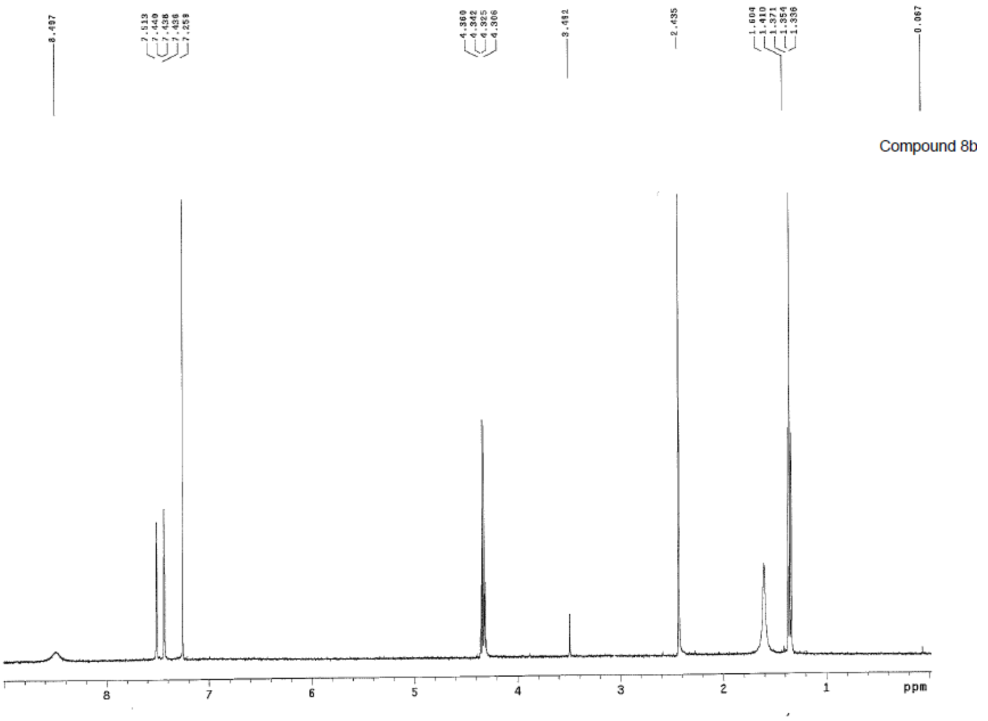
Compound 8b
ppm

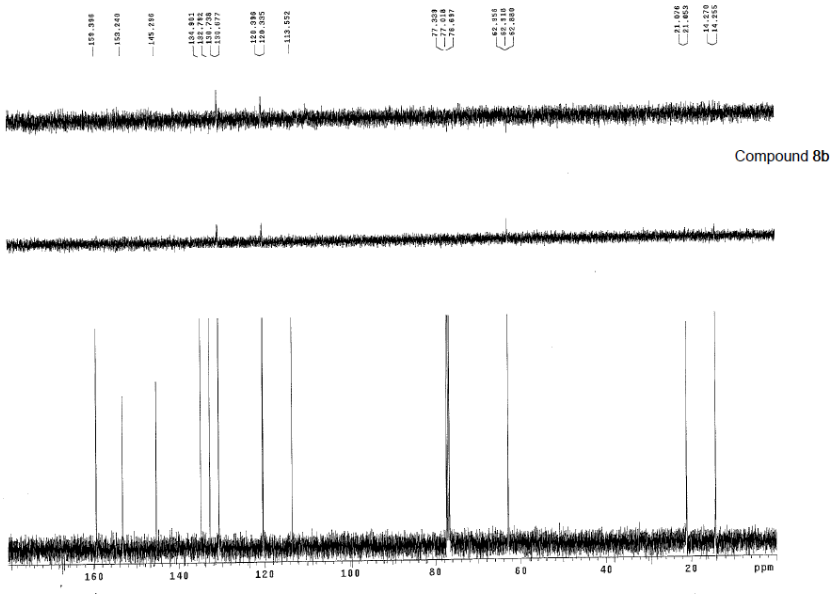
Compound 8b
ppm

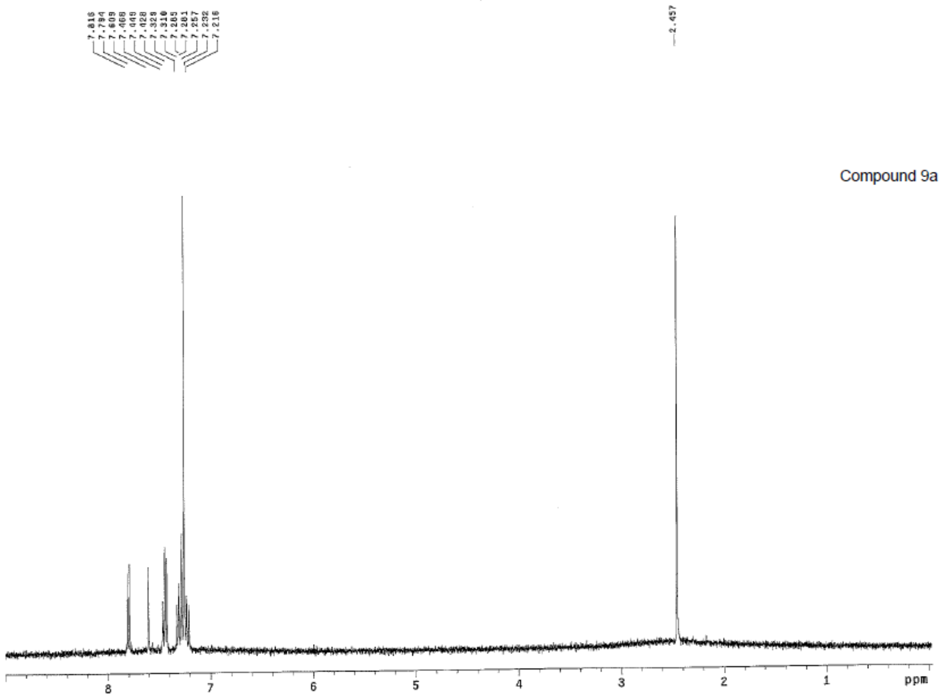
Compound 9a
ppm

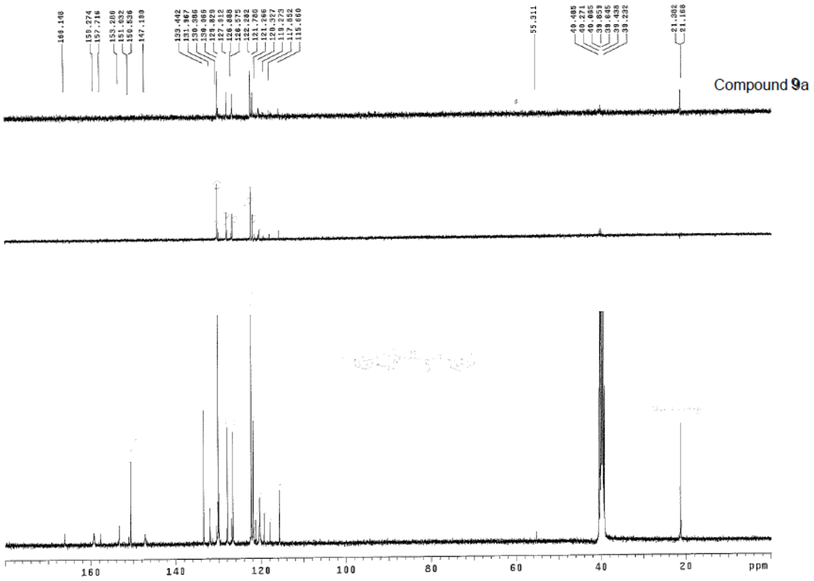
Compound 9a
ppm

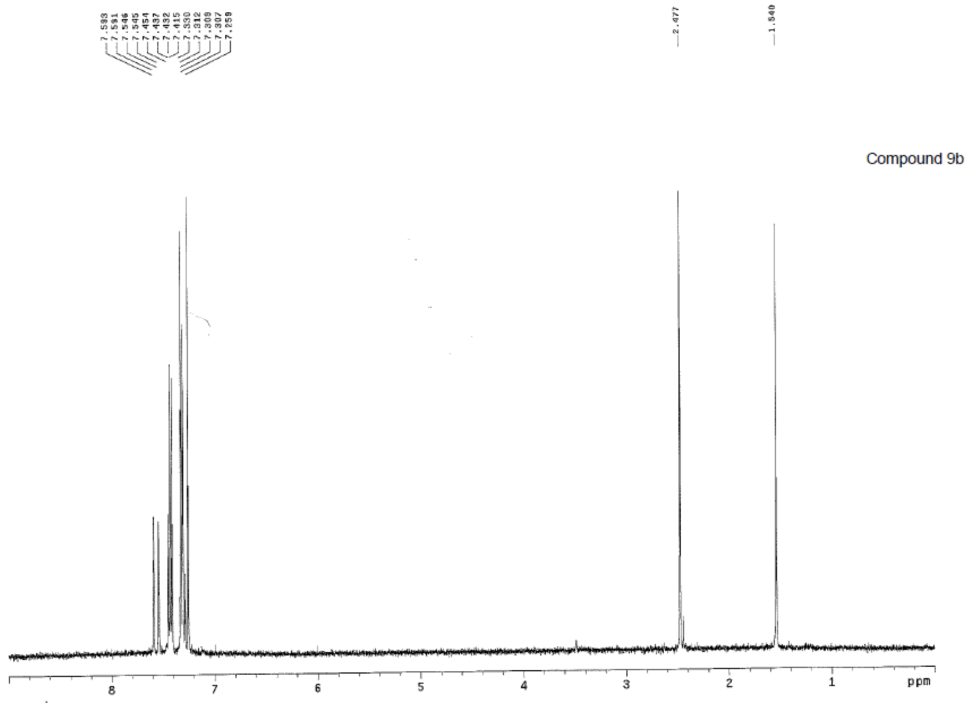
Compound 9b
ppm

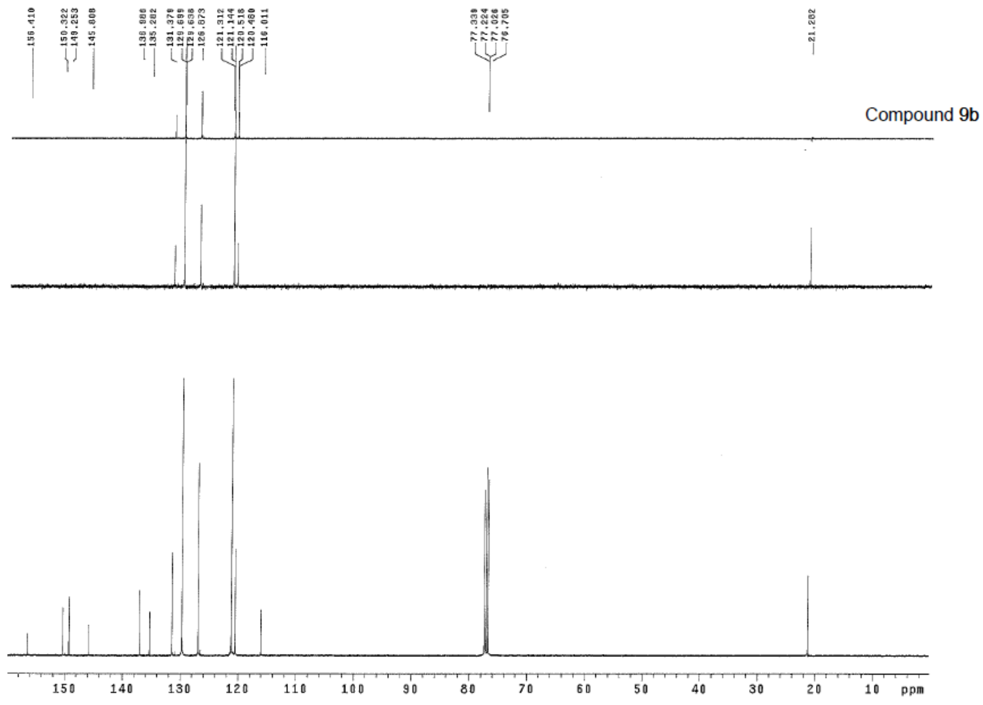
Compound 9b
ppm

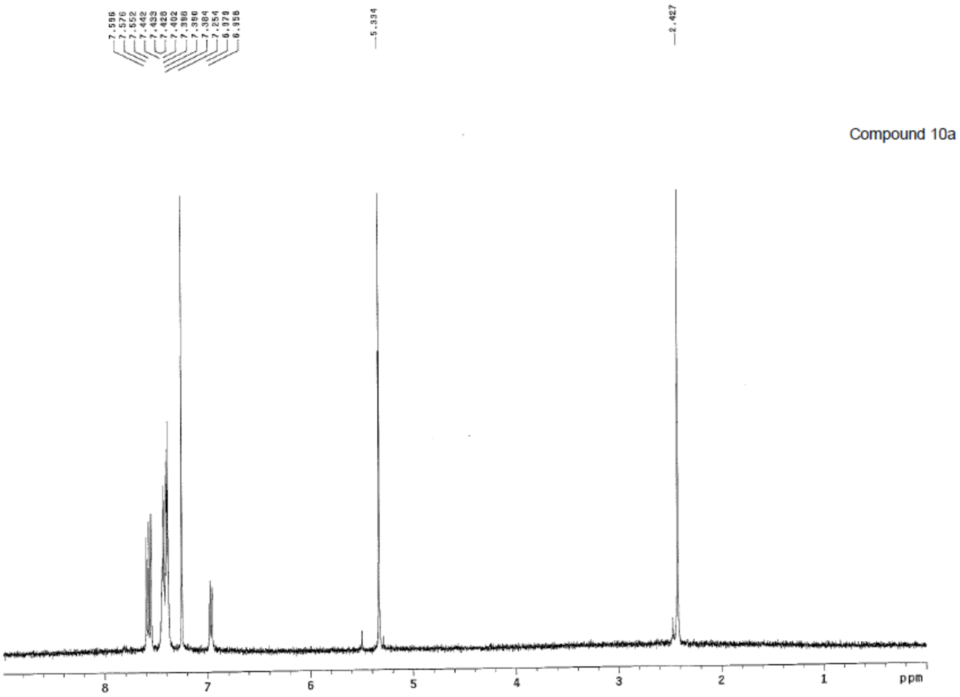
Compound 10a
8
7
6
5
4
3
2
1
ppm

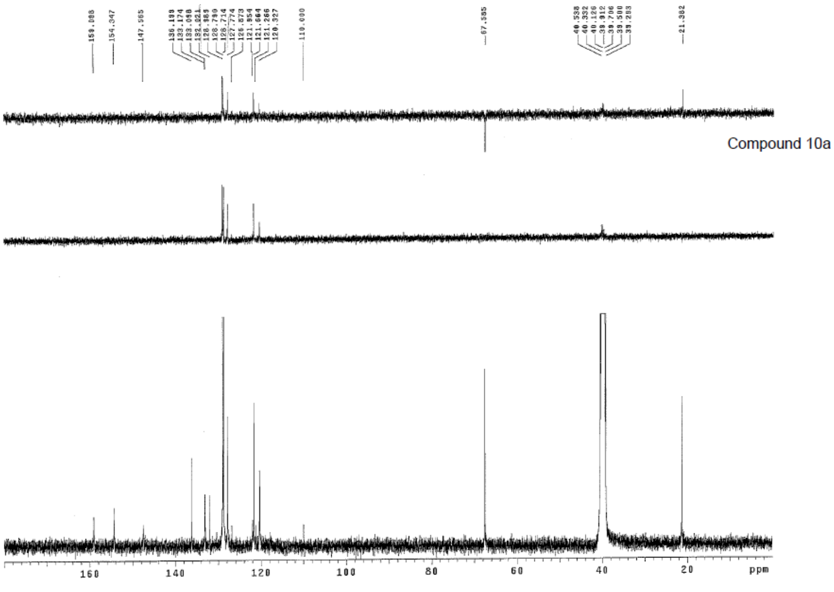
Compound 10a
160
140
120
100
80
60
40
20
ppm

1. de la Roche, N. M., T. Muhlethaler, R. M. C. Di Martino, J. A. Ortega, D. Gioia, B. Roy, A. E. Prota, M. O. Steinmetz and A. Cavalli. "Novel fragment-derived colchicine-site binders as microtubule-destabilizing agents." *Eur J Med Chem* 241 (2022): 114614. 10.1016/j.ejmech.2022.114614. https://www.ncbi.nlm.nih.gov/pubmed/35939994.
2. Wang, Y., H. Zhang, B. Gigant, Y. Yu, Y. Wu, X. Chen, Q. Lai, Z. Yang, Q. Chen and J. Yang. "Structures of a diverse set of colchicine binding site inhibitors in complex with tubulin provide a rationale for drug discovery." *FEBS J* 283 (2016): 102-11. 10.1111/febs.13555. https://www.ncbi.nlm.nih.gov/pubmed/26462166.
3. McNamara, D. E., S. Senese, T. O. Yeates and J. Z. Torres. "Structures of potent anticancer compounds bound to tubulin." *Protein Sci* 24 (2015): 1164-72. 10.1002/pro.2704. https://www.ncbi.nlm.nih.gov/pubmed/25970265 https://www.ncbi.nlm.nih.gov/pmc/articles/PMC4500315/pdf/pro0024-1164.pdf.
4. Barbier, P., A. Dorleans, F. Devred, L. Sanz, D. Allegro, C. Alfonso, M. Knossow, V. Peyrot and J. M. Andreu. "Stathmin and interfacial microtubule inhibitors recognize a naturally curved conformation of tubulin dimers." *J Biol Chem* 285 (2010): 31672-81. 10.1074/jbc.M110.141929. https://www.ncbi.nlm.nih.gov/pubmed/20675373 https://www.ncbi.nlm.nih.gov/pmc/articles/PMC2951239/pdf/zbc31672.pdf.
5. La Sala, G., N. Olieric, A. Sharma, F. Viti, F. de Asis Balaguer Perez, L. Huang, J. R. Tonra, G. K. Lloyd, S. Decherchi, J. F. Díaz, *et al.* "Structure, thermodynamics, and kinetics of plinabulin binding to two tubulin isotypes." *Chem* 5 (2019): 2969-86. 10.1016/j.chempr.2019.08.022. https://doi.org/10.1016/j.chempr.2019.08.022.
6. Bueno, O., J. Estevez Gallego, S. Martins, A. E. Prota, F. Gago, A. Gomez-SanJuan, M. J. Camarasa, I. Barasoain, M. O. Steinmetz, J. F. Diaz, *et al.* "High-affinity ligands of the colchicine domain in tubulin based on a structure-guided design." *Sci Rep* 8 (2018): 4242. 10.1038/s41598-018-22382-x. https://www.ncbi.nlm.nih.gov/pubmed/29523799 https://www.nature.com/articles/s41598-018-22382-x.pdf.
7. Sharma, A., G. Saez-Calvo, N. Olieric, F. de Asis Balaguer, I. Barasoain, C. Lamberth, J. F. Diaz and M. O. Steinmetz. "Quinolin-6-yloxyacetamides are microtubule destabilizing agents that bind to the colchicine site of tubulin." *Int J Mol Sci* 18 (2017): 10.3390/ijms18071336. https://www.ncbi.nlm.nih.gov/pubmed/28640209 https://digital.csic.es/bitstream/10261/251267/1/ijms-18-01336.pdf.
